# Preoperative Weight Loss in Patients With Excess Weight and Colorectal Cancer: The CARE Feasibility Randomized Clinical Trial

**DOI:** 10.1001/jamanetworkopen.2025.47126

**Published:** 2025-12-08

**Authors:** Dimitrios A. Koutoukidis, Susan A. Jebb, Sophie Reynolds, T. Martyn Hill, Claire Foster, Alison Horne, Pete Wheatstone, Mei-Mei Wright, Harsha Dissanayake, Joanna Snowball, Christopher Challand, Nicola Fearnhead, Robert Dennis, Beth Thompson, Richard Wilkin, Samson Tou, Sarah Hassan, Jake Foster, Marta Penna, Felix Achana, Amy Taylor, Simon J. A. Buczacki

**Affiliations:** 1 Nuffield Department of Primary Care Health Sciences, University of Oxford, Oxford, UK; 2 NIHR Oxford Biomedical Research Centre, Oxford, UK; 3 Surgical Intervention Trials Unit, Nuffield Department of Surgical Sciences, University of Oxford, Oxford, UK; 4 Centre for Psychosocial Research in Cancer, School of Health Sciences, University of Southampton, Southampton, UK; 5 Patient and Public Involvement Member, Leeds, UK; 6 Oxford University Hospitals NHS Foundation Trust, Oxford, UK; 7 Sheffield Teaching Hospitals NHS Foundation Trust, Sheffield, UK; 8 Cambridge University Hospitals NHS Foundation Trust, Cambridge, UK; 9 North West Anglia NHS Foundation Trust, Peterborough, UK; 10 Royal Devon and Exeter NHS Foundation Trust, Exeter, UK; 11 Worcestershire Acute Hospitals NHS Trust, Worcester, UK; 12 University Hospitals of Derby and Burton, NHS Foundation Trust, Derby, UK; 13 University Hospitals Dorset NHS Foundation Trust, Poole, UK; 14 Nuffield Department of Surgical Sciences, University of Oxford, Oxford, UK

## Abstract

**Importance:**

Excess weight increases morbidity risk after colorectal cancer surgery.

**Objective:**

To assess the feasibility of a preoperative weight loss intervention.

**Design, Setting, and Participants:**

CARE was an assessor-blinded randomized clinical trial performed between March 27, 2023, to August 13, 2024, with a 30-day postoperative follow-up in adults with a body mass index of 28 or greater who were awaiting curative elective colorectal cancer resection at 8 hospitals across England.

**Intervention:**

Dietetic support with a low-energy (800 kcal/d, 76 g/d of protein) total diet replacement program between decision to treat and surgery.

**Main Outcomes and Measures:**

Progression criteria for a definitive trial included recruitment (≥72 patients), engagement (≥75% of dietetic telephone calls attended), adherence (≥60% of intervention participants achieving ≥5% weight loss), and retention (≥85%). Secondary outcomes included morbidity, symptoms, and changes in weight and fat-free mass. Outcomes were modeled during a 30-year time horizon using cohort simulation.

**Results:**

Of the 150 potentially eligible participants approached, 71 eligible randomized participants (mean [SD] age, 64 [8.7] years; 43 [61%] male; mean [SD] body mass index, 35.4 [5.4]) undergoing surgery (36 in the intervention group and 35 in the usual care group) were included in the analysis. Participants were accrued at a rate of 0.57 participants per site per month compared with the anticipated rate of 0.75. Participants attended 85% of their dietetic calls. The median (IQR) period from randomization to surgery was 33 (25-43) days, during which 22 (61%) of the intervention participants and 3 (9%) of the usual care participants lost 5% or more of their weight (odds ratio, 16.8; 95% CI, 4.3-65.3). Intervention participants lost a mean (SD) of 6.1 (3.0) kg before surgery, which was 4.3 kg (95% CI, 2.7 to 5.8 kg) more than the usual care participants. Between-group adjusted change in fat-free mass was 0.1 kg (95% CI, −3.9 to 4.0 kg). There was no evidence of a difference between the 2 group in the proportion of patients with any complications (14 [39%] vs 14 [40%]) or with Clavien-Dindo grade I, II, or III complications. Fecal incontinence (−8.6 points [95% CI, −16.7 to −0.5 points]) and sore skin (−15.9 points (95% CI, −25.3 to −6.6 points]) improved postoperatively in the intervention compared with usual care group. There were no intervention-related serious adverse events. Retention was 100%. In an exploratory observational analysis, participants who lost 3.2% or more of their body weight (above the median loss) had a 50% relative reduction in complications (95% CI, 1%-78%). Modeling indicated that the intervention was cost-effective.

**Conclusions and Relevance:**

This randomized clinical trial’s intervention of low-energy total diet replacement before colorectal cancer resection was feasible and well tolerated, lacked safety concerns, and was likely cost-effective. The progression criteria were met, but some recruitment challenges need to be addressed.

**Trial Registration:**

ISRCTN.org Identifier: ISRCTN39207707

## Key Points

**Question:**

Is intensive weight loss before curative surgery in people with colorectal cancer and excess weight feasible and cost-effective?

**Findings:**

In this randomized clinical trial of 71 individuals with colorectal cancer and excess weight, participants following a low-energy total diet replacement program with dietetic support lost 4.3 kg more weight than the usual care group within 33 days until surgery with no evidence of between-group differences in fat-free mass and physical function. Modeling indicated the intervention was cost-effective.

**Meaning:**

These data provide reassurance for both patients and clinicians regarding the safety, feasibility, and likely cost-effectiveness of intentional weight loss before colorectal cancer surgery.

## Teaser

This randomized clinical trial of adults with colorectal cancer and excess weight assesses the feasibility and safety of total diet replacement for intensive weight loss before colorectal cancer resection and models the likely cost-effectiveness of such an intervention.

## Introduction

Colorectal cancer is the fourth most common cancer in Europe and the US. Surgical resection is part of the standard treatment for most patients but carries a higher risk of postoperative morbidity than many other major abdominal operatioms.^1^ Morbidity worsens patients’ quality of life^2^ and increases health care spending.^3^ Approximately two-thirds of patients with colorectal cancer carry excess weight, and half of those have obesity.^4^ Excess weight increases the risk of postoperative morbidity, independent of demographic characteristics.^5,6^ Reducing the risk of morbidity and finding effective preoperative treatments are identified research priorities.^7,8^

In bariatric surgery, preoperative weight loss is associated with better outcomes.^9^ However, the short interval between diagnosis and treatment in colorectal cancer necessitates an intensive weight loss intervention. A nutritionally complete, low-energy total diet replacement (TDR) program with behavioral support leads to approximately 7% weight loss in a month in noncancer settings.^10,11^ However, it is unclear whether people with cancer will enroll in and adhere to such a program during this unsettling period. There is little evidence from clinical trials on the benefits and risks of substantial intentional weight loss in this setting. In some observational studies,^12,13^ weight loss has been associated with worse outcomes, potentially due to muscle mass loss, leading to sarcopenia. Clinical trial evidence also suggests that mitigating weight loss might reduce morbidity.^14^ We aimed to assess the feasibility and gather evidence about the safety of TDR before colorectal cancer resection and to model the likely cost-effectiveness of such an intervention.

## Methods

### Design

CARE was a prospectively registered, multicenter, external, feasibility, parallel, individually randomized clinical trial. The study protocol has been previously published^15^ and is available in Supplement 1. There were no substantial changes to the protocol, other than minor changes to the exclusion criteria detailed in eAppendix 1 in Supplement 2. The study was approved by the South Central Oxford B Research Ethics Committee. The study followed the Consolidated Standards of Reporting Trials (CONSORT) reporting guideline.

### Setting and Participants

Participants were recruited from 9 academic and community hospitals across England (eFigure 1 in Supplement 2). Eligible participants were adults with a body mass index (BMI; calculated as weight in kilograms divided by height in meters squared) of 28 or greater (adjusted to BMI ≥25 for minority ethnic groups) and World Health Organization performance status of 0 to 2 who were listed for curative resection for colorectal cancer. Neoadjuvant treatment, if indicated, should have been completed before enrollment. Participants self-reported their ethnicity using the standard Office of National Statistics categories because the BMI criterion for the inclusion was ethnicity specific, as recommended by national obesity guidance. Participants were excluded if they reported 10% or greater weight loss in the 6 months before the screening visit, if the window between the screening visit and the surgery was less than 20 days, or if they had major comorbidities or contraindications to the intervention. Participants provided written informed consent.

### Intervention and Usual Care

In addition to their local standard care pathway, participants were asked to replace all their foods with 4 nutritionally complete meal replacement products per day (Habitual Health Ltd) until their surgery. These meals provided approximately 800 kcal/d, including 76 g/d of protein with the nutritional composition aligned with regulatory requirements. Participants had a 45-minute introductory telephone consultation with a dietitian, weekly 20-minute follow-up calls, and a 10-minute exit call. The support aimed to maintain motivation during the adjustment of meal replacement products and problem-solving issues. Participants in the control group followed their local standard care pathway.

### Primary Outcomes

We had 5 predefined feasibility aspects to assess progression to a definitive trial: recruitment (rate of patients per site per month, number of sites open, and total number of participants recruited), engagement (proportion of dietetic telephone calls answered), adherence (proportion of intervention participants with 5% or more weight loss from baseline to the day of surgery), retention (proportion at 30 days postoperatively), and safety profile (eTable 1 in Supplement 2).

### Secondary Outcomes

To assess postoperative morbidity, 2 colorectal cancer surgeons who were also independent of the local clinical teams blindly coded complications at discharge and at the 30-day postoperative follow-up using the Clavien-Dindo classification independently. Disagreements were resolved by discussion. We explored the presence of any morbidity by Clavien-Dindo grade, the highest graded morbidity, and the type of morbidity. We also converted post hoc the Clavien-Dindo grading to the Comprehensive Complication Index, which is a more sensitive end point for clinical trials.^16^ Predefined oncologic and operative outcomes were extracted from the hospital records. The participants evaluated the intervention using the Theoretical Framework of Acceptability questionnaire.^17^

### Sample Size and Randomization

We needed 72 patients (36 per arm) to have 90% power (85% collective power) at a 1-sided 5% level to detect whether the proportions for the engagement, adherence, and retention criteria in eTable 1 in Supplement 2 were above the upper red limit (>50% engagement, >35% adherence, and >65% follow-up) based on an alternative being in the green zone.^18^

Participants were individually randomized in a 1:1 ratio to one of the trial groups by a researcher at their local site using a central web-based system (REDCap-Minimization, version 1.2.2).^19^ The minimization algorithm included a 20% random element and was stratified by performance status (0 vs 1-2) and age (<70 years or ≥70 years). Allocation was concealed (eAppendix 2 in Supplement 2).

### Patient and Public Involvement

Patients with colorectal cancer, their relatives, and members of the public helped to develop and design this research. Patients with colorectal cancer codesigned participant-facing and dissemination materials and contributed to the study management and data interpretation.

### Cost-Utility Modeling

Using incremental cost-utility analysis, we modeled a cohort of patients undergoing colorectal cancer resection in the UK. Conservatively, we assumed a 20% relative reduction in the postoperative morbidity (eTable 2 in Supplement 2). We developed a hybrid model with a decision tree component for the first 30 days postoperatively and a multivariable probabilistic Markov component, after which patients transitioned between states for 30 yearly cycles (eAppendix 3 and eFigure 2 in Supplement 2).

### Statistical Analysis

Progression criteria are presented as means (SDs) or numbers (percentages), as appropriate. Secondary outcomes were analyzed using mixed-effects models or regression models as appropriate. The main analysis followed the intention-to-treat approach with a per-protocol analysis based on achieved weight loss (eAppendix 2 in Supplement 2). Missing data were not imputed. The level of statistical significance was *P* < .05 (1-sided for progression criteria and 2-sided for all other outcomes). Data were primarily analyzed using Stata software, version 15.1 (StataCorp).

## Results

### Recruitment and Retention

Targets of 2 of the 3 progression criteria for recruitment (number of open sites and number of participants recruited) were met with no need for further improvement. However, the recruitment rate was 0.57 participants per site per month, meaning that this criterion fell under progress with changes because it was lower than 0.75 participants per site per month (eAppendix 4 and eFigure 3 in Supplement 2). Retention was 100%, exceeding the progression criterion target.

### Baseline Characteristics and Surgical Procedure

**Figure 1.**
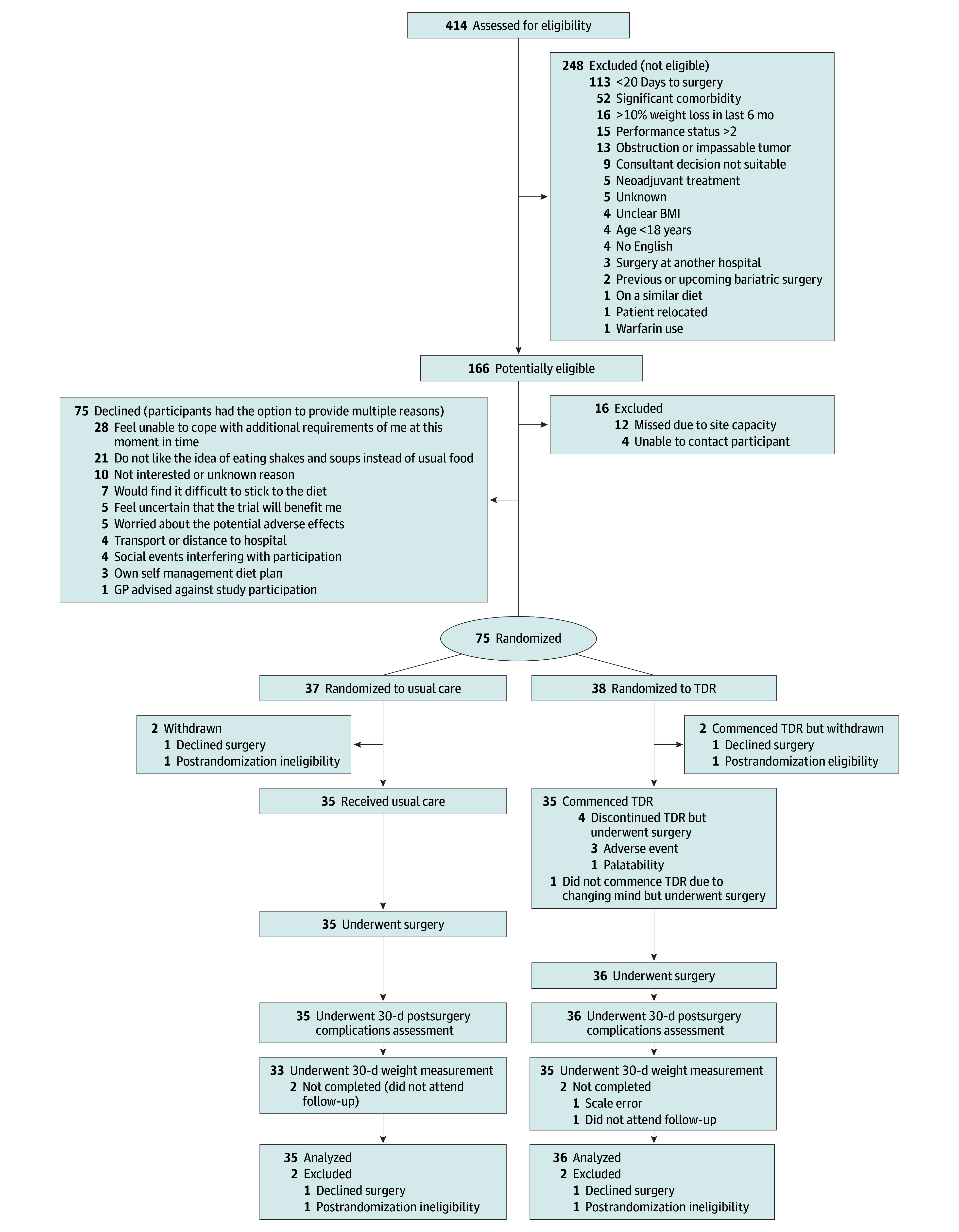
CONSORT Diagram of Participant Flow Through the Trial BMI indicates body mass index; CONSORT, Consolidated Standards of Reporting Trials; GP, general practitioner; TDR, total diet replacement.

Seventy-one eligible participants were randomized to either the usual care (n = 35) or intervention (n = 36) groups (Figure 1). Participants had a mean (SD) age of 64 (8.7) years, 43 (61%) were male and 28 (39%) were female, 1 (3%) was Asian, 1 (3%) was Black, 68 (96%) were White, and 1 (3%) was other race (no additional information available for this category), and the mean (SD) baseline BMI was 35.4 (5.4). Additional baseline characteristics are given in eTable 3 in Supplement 2. Among all participants, 21 (30%) could not perform all normal activities without restriction (scored 1-2), and 62 (87%) had mild or severe systemic disease (American Society of Anesthesiologists scores of 2-3). The median (IQR) times from the first multidisciplinary team meeting discussion to randomization and from randomization to surgery were 17 (27.5) and 33 (17.5) days, respectively. The most common surgical approaches were laparoscopic (39 [55%]) and robotic (19 [27%]), with anterior resection (37 [52%]) and right hemicolectomy (16 [23%]) being the most common procedures. Nineteen patients (27%) had stomas. The staging and additional clinical characteristics are presented in eTable 4 in Supplement 2.

### Intervention Engagement

Of the 36 participants in the intervention arm who were included in the analysis, 35 (97%) started the intervention and 32 (89%) completed it. Among all 36 participants, the mean (SD) proportion of expected dietetic calls attended per participant was 85% (24%). A total of 21 participants (58%) attended all calls. Adverse events in the presence of long preoperative pathways were the main reason for discontinuation as detailed in eAppendix 5 in Supplement 2.

### Intervention Adherence

Compared with the usual care group, participants in the intervention group lost an additional 4.3 kg (95% CI, 2.7-5.8 kg) before surgery and maintained that between-group difference at 30 days postoperatively. Only 3 participants (8%) in the intervention group were classed as nonadherent (ie, <2% weight loss). The trial exceeded the progression target for adherence with 22 (61%) and 3 (9%) individuals in the intervention and usual care groups, respectively, losing 5% or more of their body weight between randomization and surgery (odds ratio, 16.8; 95% CI, 4.3-65.3). There was a positive association between weight change and length of time from randomization to surgery in the intervention group (*r* = 0.38; *P* = .02; n = 35). Most usual care group participants (27 [82%]) reported changing their diet and/or physical activity in some way before surgery, but none attended a formal weight loss program as detailed in eAppendix 6 in Supplement 2.

### Intervention Evaluation

The intervention met or exceeded the expectations of 21 (79%) of the intervention participants providing feedback (2 with missing data) as detailed in eAppendix 7 and eFigure 4 in Supplement 2. Participants were more likely to report that it took no or a little effort vs a lot or a huge effort to follow the diet (64% vs 33%; odds of reporting less effort, 1.9), and they also were more likely or extremely likely to recommend the program to someone else with excess weight awaiting bowel cancer surgery than not (79% vs 9%; odds of recommending, 9).

### Oncologic and Operative Outcomes

None of the participants had involved resection margins. There was no evidence of between-group difference in circumferential resection margins, operative time, conversion to open surgery, length of initial hospital stay, reoperation rates, readmission rates, or 30 days alive and out of the hospital (eTable 5 in Supplement 2). During the first hospital stay, 2 patients in each group were admitted to the intensive care unit. Three participants were admitted to the high dependency unit in the intervention group and 1 in the control group.

### Postoperative Morbidity

**Figure 2.**
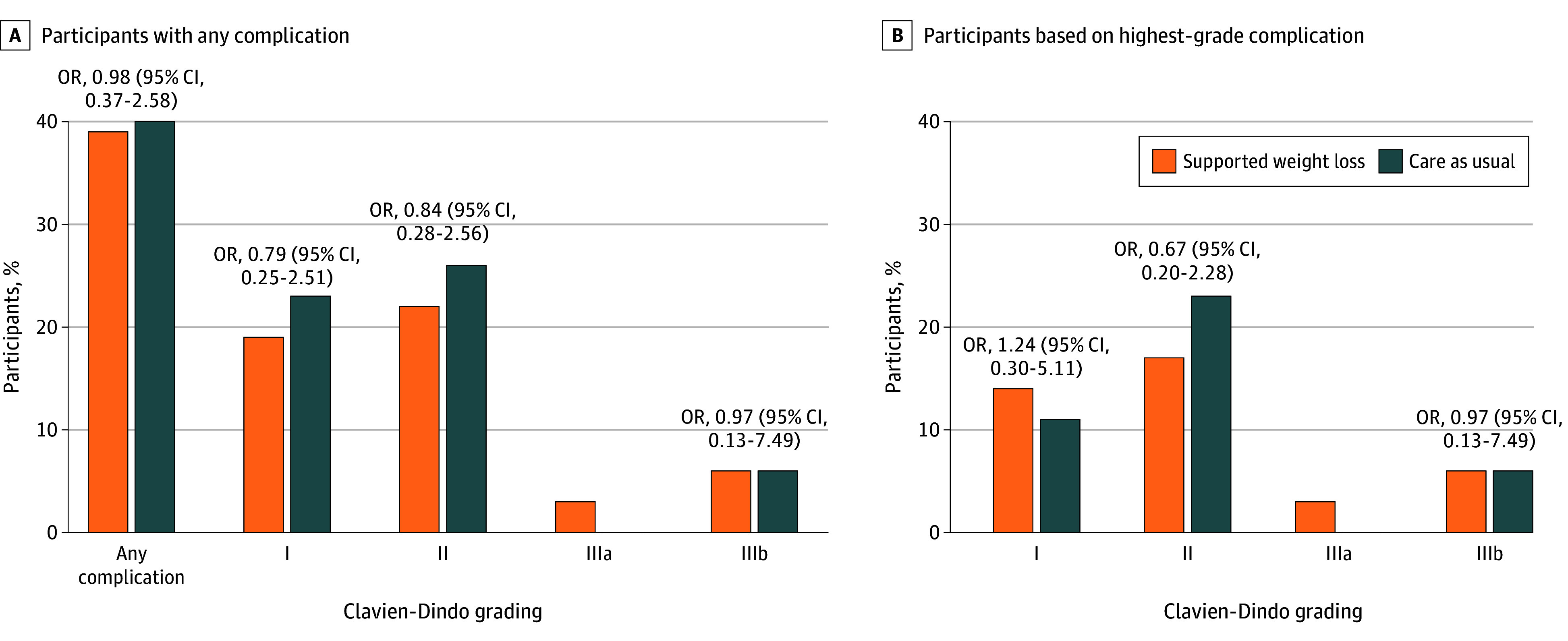
Proportion of Participants With Complications Up to 30 Days Postoperatively by Group Based on the Clavien-Dindo Classification A, The proportion of participants with at least one complication on each grade. B, The proportion of participants based on the highest-graded complication they experienced. OR indicates odds ratio.

**Figure 3.**
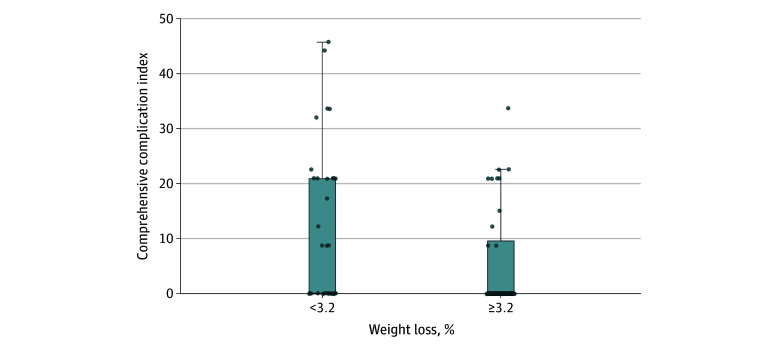
Median Comprehensive Complication Index Scores by Median Weight Loss This box-and-whisker plot shows the distribution of Comprehensive Complication Index scores by median weight loss. Dots represent the Comprehensive Complication Indexes of individual participants. Whiskers indicate IQRs. The top of the box presents the third quartile. The first quartile is 0, and the median value is also 0. The top of the black line represents the highest values, and the individual point beyond that black line in the second box is an outlier.

Fourteen patients (39%) in the intervention group and 14 (40%) in the control group experienced at least 1 complication by 30 days postoperatively (Figure 2). Overall, 22 and 29 complications were reported in the intervention and usual care groups, respectively, with no evidence that the number of complications per patient differed between groups among those with at least 1 complication (between-group difference, −0.4 points; 95% CI, −1.6 to 0.7 points). Most complications were graded as I or II according to the Clavien-Dindo classification. There was no evidence that the proportion of participants with grade I, II, or III complications differed between groups. The between-group difference in the Comprehensive Complication Index was −0.5 points (95% CI, −6.4 to 5.5 points). The most common type of complications was gastrointestinal in both groups (eTable 6 in Supplement 2). No grade IV complications or deaths were observed. In an exploratory observational analysis, participants who lost 3.2% or more of their weight (ie, above the median weight loss) in the whole cohort had a 50% relative reduction in their Comprehensive Complication Index (95% CI, 1%-78%) (Figure 3) compared with those who lost less. A similar estimate was observed for the presence of any complications (odds ratio, 0.38; 95% CI, 0.13-1.06).

### Body Composition and Physical Function

**Figure 4.**
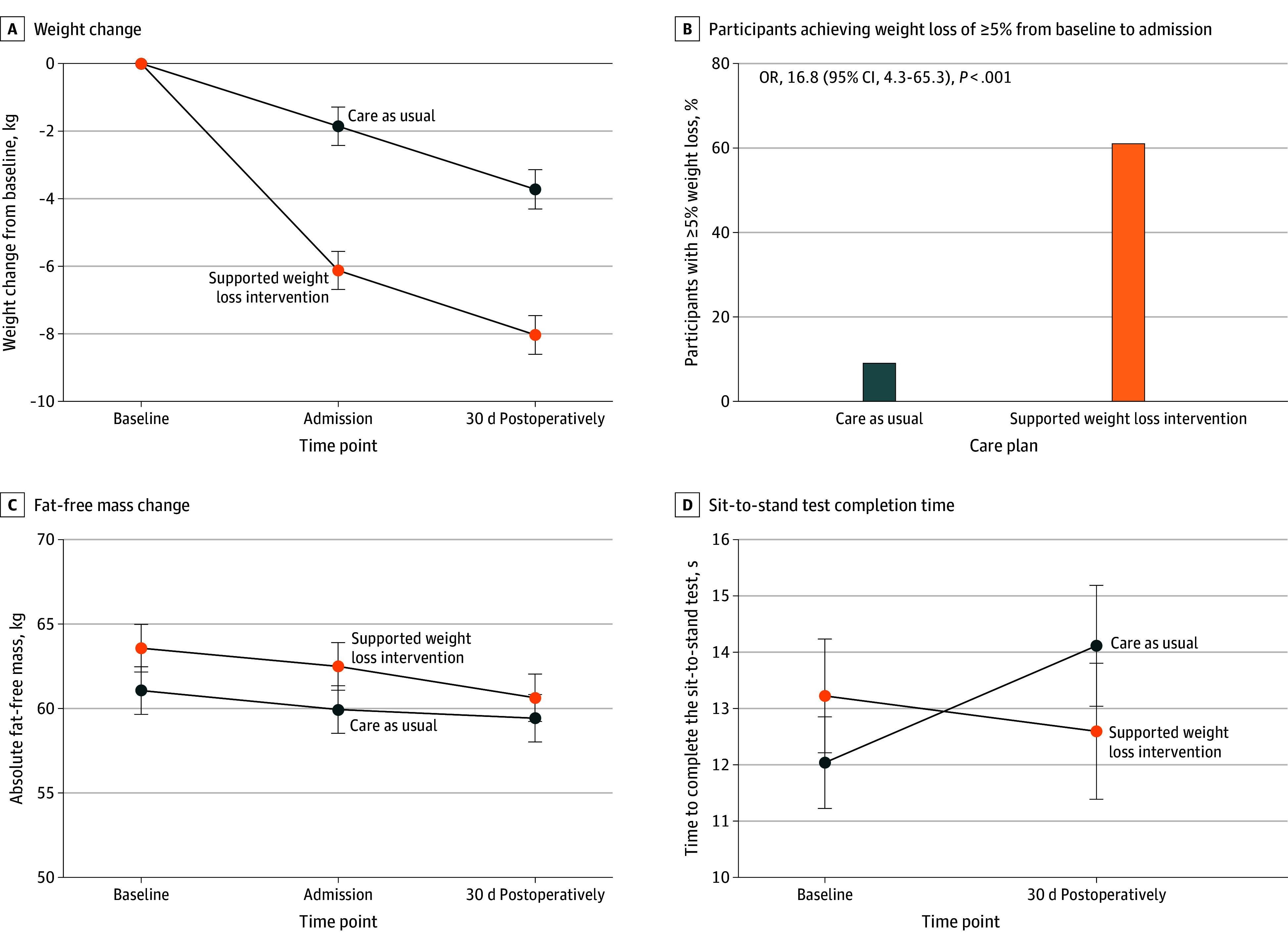
Mean Changes in Weight and Fat-Free Mass and of Time to Complete the Sit-to-Stand Test and Proportion of Participants in Each Group Achieving a Weight Loss of 5% or Greater From Baseline to Admission Error bars indicate SEs. OR indicates odds ratio.

Compared with usual care, intervention participants preserved both their absolute fat-free mass (between-group difference, 0.1 kg; 95% CI, −3.9 to 4.0 kg) and relative fat-free mass (percentage of total weight, 2.3%; 95% CI, −1.1%-5.8%). There was no significant difference in the time to complete the sit-to-stand test between the groups at 30 days postoperatively (between-group difference, −1.5 seconds; 95% CI, −4.4 to 1.3 seconds) (Figure 4; eTable 7 in Supplement 2).

### Health-Related Quality of Life, Anxiety, and Depression

There was no evidence of a difference in the change in anxiety or depression between groups (eTable 7 in Supplement 2). There was no evidence that the proportion of participants with changes (increases or decreases) in anxiety or depression of at least the minimal clinically important difference by the time of surgery differed between the groups (eTable 8 in Supplement 2). There was no evidence of changes in quality of life based on the 5-level EQ-5D version descriptive system and visual analog scale. At 30 days postoperatively, the intervention participants reported better body image and less worry about weight. Among those without a stoma, intervention participants reported less fecal incontinence (−8.6 points [95% CI, −16.7 to −0.5 points]) and sore skin (−15.9 points [95% CI, −25.3 to −6.6 points]) than those in usual care. There was no evidence of differences in the remaining European Organisation for Research and Treatment of Cancer scales and symptoms (Table).

**Table.** European Organisation for Research and Treatment of Cancer–Quality of Life Questionnaire–C29 Scores at 30 Days Postoperatively^a^

Measure	Mean (SD) score		Adjusted difference (95% CI)
	Intervention group	Usual care group	
Functional scales			
Anxiety	67.6 (22.1)	63.6 (26.8)	4.9 (−6.6 to 16.4)
Weight	77.1 (31.1)	61.6 (26.5)	15.8 (1.6 to 29.9)
Body image	91.7 (12.5)	76.4 (23.7)	15.6 (6.8 to 24.4)
Sexual interest	27.5 (32.3)	17.2 (23.8)	8.3 (−4.9 to 21.4)
Symptoms			
Urinary frequency	43.1 (25.0)	47.0 (23.7)	−4.0 (−16.1 to 8.1)
Urinary incontinence	5.9 (12.9)	15.2 (25.1)	−8.6 (−17.8 to 0.6)
Dysuria	5.9 (12.9)	11.1 (19.8)	−5.1 (−13.0 to 2.8)
Abdominal pain	24.8 (23.4)	26.3 (26.0)	−1.9 (−14.0 to 10.1)
Buttock pain	20.0 (27.1)	21.2 (27.4)	−1.5 (−14.9 to 11.8)
Bloating	16.2 (20.4)	20.2 (23.5)	−4.2 (−15.1 to 6.7)
Blood and mucus in stool	2.4 (7.2)	5.1 (8.8)	−2.7 (−6.6 to 1.2)
Dry mouth	21.9 (30.2)	25.3 (31.2)	−3.0 (−18.1 to 12.1)
Hair loss	2.9 (12.5)	2.0 (8.1)	1.0 (−4.4 to 6.1)
Taste	9.5 (15.3)	17.2 (32.4)	−7.8 (−19.8 to 4.3)
Flatulence (no stoma)	23.6 (25.0)	22.7 (23.0)	0.8 (−12.7 to 14.4)
Flatulence (stoma)	30.3 (27.7)	37.5 (37.5)	−17.0 (−48.7 to 14.7)
Fecal incontinence (no stoma)	1.4 (6.8)	9.3 (18.1)	−8.6 (−16.7 to −0.5)
Fecal incontinence (stoma)	15.2 (17.4)	29.2 (27.8)	−14.3 (−33.7 to 5.1)
Sore skin (no stoma)	1.4 (6.8)	16.0 (21.8)	−15.9 (−25.3 to −6.6)
Sore skin (stoma)	33.3 (25.8)	41.7 (23.6)	−5.4 (−34.2 to 23.3)
Stool frequency (stoma)	10.6 (11.2)	18.8 (10.7)	−11.2 (−22.6 to 0.1)
Stool frequency (no stoma)	25.0 (20.3)	24.0 (21.0)	−0.6 (−11.4 to 10.3)
Embarrassment (no stoma)	4.2 (15.0)	9.3 (18.1)	−5.0 (−14.8 to 4.8)
Embarrassment (stoma)	15.2 (17.4)	29.2 (37.5)	−11.6 (−40.8 to 17.7)
Stoma care problems (stoma)	9.1 (15.6)	25.0 (34.5)	−8.2 (−33.3 to 17.0)
Impotence (men)	24.2 (31.2)	19.6 (29.0)	6.9 (−13.8 to 27.6)
Dyspareunia (women)	0.0 (0.0)	2.1 (8.3)	−2.4 (−7.7 to 3.0)

^a^ The range for all scores is 0 to 100. High scores for the functional scales and items represent a high level of functioning. High scores for the symptom scales and items represent a high level of symptoms or problems.

### Adverse Events 

The adverse events are detailed in eAppendix 8 in Supplement 2. Serious intervention-related adverse events were not observed.

### Per-Protocol Analysis

Participants who adhered to the intervention (ie, lost ≥5%; n = 22) lost a mean (SD) of −7.8 (2.2) kg before surgery. The between-group difference in weight change from baseline to surgery was more profound than in the intention-to-treat analysis at −6.4 kg (95% CI, −8.1 to −4.7 kg). There was no evidence of between-group differences in complications or changes in absolute fat-free mass, percentage fat-free mass, sit-to-stand test, quality of life, anxiety, or depression (eFigure 5 and eTables 9 and 10 in Supplement 2).

### Sensitivity and Subgroup Analysis

Analysis by age and performance status suggested that the intervention improved the quality of life of patients older than 70 years while awaiting surgery (eFigure 6 in the Supplement 2). There was no evidence that age or performance status moderated the effect estimates for other outcomes (eTable 11 in the Supplement 2). Analysis by colon or rectum did not show evidence of difference in complications, operative time, or length of hospital stay (eTable 12 in the Supplement 2).

### Cost-Effectiveness Analysis

The incremental cost-effectiveness ratio was estimated to be £7623 per quality-adjusted life-year (eFigure 7 in Supplement 2), and the probability that the intervention would be cost-effective at the conventional willingness-to-pay threshold of £20 000 was 61% (eFigure 8 in Supplement 2). The results were consistent across deterministic and probabilistic analyses with no material changes in the sensitivity analyses (eTable 13 in Supplement 2).

## Discussion

In this randomized clinical trial, an intensive weight loss intervention was feasible and safe for patients with excess weight awaiting colorectal cancer resection. Compared with usual care, intervention participants lost weight without compromising their fat-free mass and had fewer symptoms affecting life after surgery. Participants rated the intervention very positively. Adverse events were mainly mild or moderate. There was preliminary evidence of a reduction in postoperative morbidity; people who lost more than the median percentage of weight (≥3.2%) had a 50% relative reduction in complications compared with those who lost less. These data provide the necessary platform to progress to a definitive trial to formally test whether the intervention reduces postoperative complications.

Although the proportion of eligible people enrolling (50%) was similar to those of prehabilitation trials of other behavior change interventions,^20,21,22^ the trial did not fully meet one of its recruitment targets (rate of randomized patients per site per month). There are several reasons for this finding. We found variable clinical pathways, with a combination of longer than anticipated periods between diagnosis and decision to treat, shorter periods between decision to treat and surgery, unwillingness to accommodate a minimum of a 20-day period from randomization to surgery due to clinical pressures, and preference of some clinicians and researchers to arrange an additional consultation for the trial after the consultation during which they discussed surgery due to the already long and overwhelming nature of the latter. Recruitment was hindered by limited research capacity at sites, other trials competing for patients and staff time, and buy-in to the study from some consultant surgeons. Some of these hurdles could be addressed in future trials, including online-only trial assessments that reduce the need and resources for additional in-person consultations and minimize delays to randomization and communication of the acceptability of the intervention during the feasibility trial.

The weight loss achieved is consistent with that observed in trials of similar TDR interventions in other settings.^10,23,24^ This finding indicates that for those enrolled in the trial, there do not seem to be additional barriers to adherence due to the added burden of a cancer diagnosis and prospect of a major operation. Some clinicians at the start of the trial expressed concerns that weight loss may increase the risk to patients owing to reductions in muscle mass. Our data can reassure clinicians because there was no evidence that fat-free mass or physical function changed and most of the lost weight was due to loss of fat mass. The preservation of fat-free mass and physical function is consistent with other populations with excess weight who are typically considered at high risk of sarcopenia and frailty and enroll in similar weight loss programs.^25^ Additionally, intervention participants experienced fewer symptoms postoperatively, including fecal incontinence and sore skin. Fecal incontinence has been recognized as a key patient-reported outcome in colorectal cancer surgery trials.^26^ The overall direction of change across symptoms indicated that intervention participants had fewer of most symptoms, but the wide CIs precluded firm conclusions.

The proportion and type of complications as well as the Comprehensive Complication Index were broadly similar to other prehabilitation intervention trials in this population.^20,21^ Although limited by sample size, as ours is, these trials have collectively shown encouraging signs of reduced complications.^27,28,29,30^ In our study, the direction of effect indicating no evidence of worsening complications combined with the signal in the exploratory observational analysis of reduced complications with greater weight loss suggest that a large-scale definitive trial is warranted. If the effect observed in other prehabilitation trials holds true here,^27^ our modeling suggests that the weight loss intervention is likely to be cost-effective to the health care system at the conventional willingness-to-pay threshold.

### Strengths and Limitations

Strengths of the study include the randomized design, recruitment from geographically and socioeconomically diverse areas, recruitment of a cohort that resembles the deprivation index of the recruitment areas, high adherence and retention, low rates of missing data, and blinded clinical outcome assessment. Limitations include the lack of power to detect or rule out observed mean differences in secondary and exploratory outcomes with precision. Confirmation of these results would require a definitive trial. Future trials should consider the Comprehensive Complication Index as a more sensitive end point. Participants in the usual care group lost almost 2 kg within the month between randomization and surgery, and most reported conscious efforts to lose weight, which might not be representative of usual care but reflect a halo effect of their engagement in a weight loss trial. This finding suggests that even a brief conversation with their surgeon about weight can motivate patients to lose some weight. Performance status was somewhat higher than in population-based cohorts,^31^ suggesting that future studies should actively aim to include more frail patients because the benefits could be greater in this group.^32,33^ The weekly contact with the study dietitian before surgery may have contributed to the reporting of more adverse events in the intervention group.

## Conclusions

An intensive preoperative weight loss intervention was safe, feasible, and likely cost-effective as part of prehabilitation before colorectal cancer surgery with evidence of improvements in key symptoms. Some challenges to recruitment need to be addressed before a definitive trial assesses perioperative and longer-term outcomes.

## Notes

Supplement 1.
Trial Protocol and Statistical Analysis Plan

Supplement 2. **eAppendix 1.** Changes to inclusion/exclusion criteria **eFigure 1**. Location of recruiting hospitals and annual case load per NHS Trust of elective major colorectal cancer resections without distant metastasis **eTable 1**. Pre-defined progression criteria to a definitive trial **eAppendix 2.** Allocation concealment, blinding, and statistical analysis **eAppendix 3.** Cost-utility modelling methods **eTable 2**. Justification of model parameters **eFigure 2**. Cost-effectiveness model of the intervention **eAppendix 4.** Recruitment **eFigure 3**. Planned vs actual recruitment rate (excluding participants who were withdrawn and replaced) (1 site closed early due to lack of recruitment) **eTable 3**. Demographic and operative characteristics **eTable 4**. Staging and clinical characteristics **eAppendix 5.** Reasons for intervention discontinuation **eAppendix 6.** Usual care group changes in relevant diet and physical activity behaviours **eAppendix 7.** Intervention evaluation **eFigure 4**. Intervention evaluation **eTable 5**. Oncologic and operative outcomes **eTable 6**. Complications by type (intention-to-treat) **eTable 7**. Weight, body composition, physical function, and quality of life **eTable 8. **Sensitivity analysis of the number and proportion of participants with minimally clinically important increase and reduction in anxiety and depression (by ≥1.7 points) between baseline and admission by group **eAppendix 8.** Adverse events **eFigure 5**. Complications by group based on the per protocol analysis **eTable 9**. Complications by type and group based in the per-protocol analysis **eTable 10**. Per-protocol analysis of anthropometric and quality of life outcomes **eFigure 6**. Interaction of quality of life (EQ-5D visual analogue scale), timepoint, and age **eTable 11. **P-values of interaction terms from sensitivity analysis stratified by age and performance status **eTable 12**. Subgroup analysis by colon (n=55) or rectum (n=16) in the intention-to-treat population **eFigure 7**. Incremental cost-effectiveness plane **eFigure 8.** Cost-effectiveness acceptability curve for the reference case analysis **eTable 13**. Incremental cost-effectiveness ratio (ICER) based on the reference case and sensitivity analyses
eReferences

Supplement 3.
Data Sharing Statement
