# Supplementary material for: Preoperative Weight Loss in Patients With Excess Weight and Colorectal Cancer: The CARE Feasibility Randomized Clinical Trial
Source: JAMA Netw Open. 2025 Dec 8;8(12):e2547126. doi: 10.1001/jamanetworkopen.2025.47126 (PMC12687096; doi:10.1001/jamanetworkopen.2025.47126)
Supplement: Supplement 1. — Trial Protocol and Statistical Analysis Plan [file jamanetwopen-e2547126-s001.pdf]

Study Title: Pre-operative intentional weight loss to support post-operative recovery in patients with overweight and colorectal cancer: the CARE feasibility randomised controlled trial

Short title: Could supported weight loss reduce bowel cancer surgery complications?

Ethics Ref: 22/SC/0465

IRAS Project ID: 320173

Date and Version No: 19 June 2024, V6.0

**Chief Investigator:**	Dr Dimitrios Koutoukidis, Senior Research Fellow, Nuffield Dept. of Primary Care Health Sciences, University of Oxford
**Co-chief investigator:**	Prof Simon Buczacki, Richard Blackwell Pharsalia Professor of Colorectal Surgery, Nuffield Dept. of Surgical Sciences, University of Oxford
**Investigators:**	Prof Susan Jebb, Professor of Diet and Population Health, Nuffield Dept. of Primary Care Health Sciences, University of Oxford
	Prof Claire Foster, Professor of Psychosocial Oncology, Centre for Psychosocial Research in Cancer, University of Southampton
	Dr Felix Achana, Senior Researcher in Health Economics, University of Oxford
	Peter Wheatstone, Patient and public representative
**Sponsor:**	University of Oxford
	Joint Research Office, Churchill Drive, Headington, Oxford OX3 7GB
**Funder:**	National Institute for Health Research

Chief Investigator Signature:

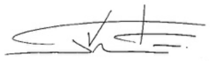

Statistician Signature: 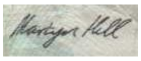

Conflicts of interest: DK and SAJ report being investigators in two publicly-funded (NIHR) trial where the weight loss intervention was donated by Nestle Health Science and Oviva to the University of Oxford outside the submitted work. No other conflicts of interest are reported.

Confidentiality Statement: This document contains confidential information that must not be disclosed to anyone other than the Sponsor, the Investigator Team, HRA, host organisation, and members of the Research Ethics Committee, unless authorised to do so.

TABLE OF CONTENTS

1. KEY CONTACTS 5
2. LAY SUMMARY 6
3. SYNOPSIS 7
4. ABBREVIATIONS 10
5. BACKGROUND AND RATIONALE 11
6. OBJECTIVES AND OUTCOME MEASURES 13
7. STUDY DESIGN 13
8. PARTICIPANT IDENTIFICATION 13
   8.1. Study Participants 13
   8.2. Inclusion Criteria 13
   8.3. Exclusion Criteria 13
9. PROTOCOL PROCEDURES 14
   9.1. Recruitment 14
   9.2. Informed Consent 16
   9.3. Screening and Eligibility Assessment 16
   9.4. Randomisation 16
   9.5. Blinding and code-breaking 17
   9.6. Description of study intervention(s), comparators and study procedures (clinical) 17
      9.6.1. Description of study intervention(s) 17
      9.6.2. Description of comparator 19
      9.6.3. Cancer waiting times 19
      9.6.4. Description of study procedure(s) 19
      9.6.5. QuinteT evaluation and optimisation of the recruitment process 23
      QuinteT Step 1: Understand recruitment as it happens 23
      QuinteT Step 2: Feedback and script piloting 24
      QuinteT Step 3: Optimising recruitment 24
      QuinteT Step 4: Facilitating enrolment 24
   9.7. Baseline Assessments 25
   9.8. Subsequent Assessments 25
   9.9. Sample Handling 26
   9.10. Early Discontinuation/Withdrawal of Participants 26
   9.11. Definition of End of Study 28

10. SAFETY REPORTING 28
10.1. Definition of Serious Adverse Events 28
10.2. Reporting Procedures for Serious Adverse Events 29
11. STATISTICS AND ANALYSIS 29
11.1. Statistical Analysis Plan (SAP) 29
11.2. Description of the Statistical Methods 29
11.3. Sample Size Determination 30
11.4. Analysis populations 30
11.5. Decision points 31
11.6. Stopping rules 31
11.7. The Level of Statistical Significance 31
11.8. Procedure for Accounting for Missing, Unused, and Spurious Data. 31
11.9. Procedures for Reporting any Deviation(s) from the Original Statistical Plan 31
11.10. Qualitative Analysis 31
11.11. Health Economic analysis 31
12. DATA MANAGEMENT 32
12.1. Source Data 32
12.2. Access to Data 32
12.3. Data Recording and Record Keeping 32
13. QUALITY ASSURANCE PROCEDURES 33
13.1. Risk assessment 33
13.2. Study monitoring 33
13.3. Study Committees 34
14. PROTOCOL DEVIATIONS 34
15. SERIOUS BREACHES 34
16. ETHICAL AND REGULATORY CONSIDERATIONS 35
16.1. Declaration of Helsinki 35
16.2. Guidelines for Good Clinical Practice 35
16.3. Approvals 35
16.4. Other Ethical Considerations 35
16.5. Reporting 35
16.6. Transparency in Research 36
16.7. Participant Confidentiality 36
16.8. Expenses and Benefits 36

17. FINANCE AND INSURANCE 36
17.1. Funding 36
17.2. Insurance 36
17.3. Contractual arrangements 36
18. PUBLICATION POLICY 36
19. DEVELOPMENT OF A NEW PRODUCT/ PROCESS OR THE GENERATION OF INTELLECTUAL PROPERTY 37
19. ARCHIVING 37
20. REFERENCES 37
21. APPENDIX A: STUDY FLOW CHART 43
22. APPENDIX B: SCHEDULE OF STUDY PROCEDURES 44
23. APPENDIX C: QUINTET CONSENT PROCESS 45
24. APPENDIX D: GUIDE FOR THE QUINTET QUALITATIVE INTERVIEW WITH PARTICIPANTS POST-RANDOMISATION 46
25. APPENDIX E: GUIDE FOR QUALITATIVE INTERVIEW WITH INTERVENTION PARTICIPANTS 47
26. APPENDIX F: GUIDE FOR QUALITATIVE INTERVIEW WITH STAFF 49
27. APPENDIX G: AMENDMENT HISTORY 50

1. KEY CONTACTS

**Chief Investigator**	Dr Dimitrios Koutoukidis<br><br>Nuffield Department of Primary Care Health Sciences, University of Oxford, Radcliffe Observatory Quarter, Woodstock Road, Oxford, OX2 6GG<br><br>T: +44 (0)1865 617767 E: dimitrios.koutoukidis@phc.ox.ac.uk
**Sponsor**	University of Oxford<br><br>RGEA, Joint Research Office, Boundary Brook House, Churchill Drive, Headington, Oxford OX3 7GB<br><br>T: +44 (0)1865 616480 E: rgea.sponsor@admin.ox.ac.uk
**Funder(s)**	National Institute for Health Research<br><br>Elisabeth Kugelberg, Research for Patient Benefit Programme Manager<br><br>T: +44 020 3692 7941 E: elisabeth.kugelberg@nihr.ac.uk
**Clinical Trials Unit**	Surgical Intervention Trials Unit, University of Oxford<br><br>T: 01865 223491, E: situ@nds.ox.ac.uk
**Statistician**	Martyn Hill, MSc<br><br>E: martyn.hill@nds.ox.ac.uk
**Committees**	Trial Steering Committee Chair: Mr James Hernon, Consultant Colorectal Surgeon, Norfolk and Norwich University Hospitals NHS Foundation Trust, T: 01603 287688, E: james.hernon@nnuh.nhs.uk<br><br>Trial Management Committee Chair: Dr Dimitrios Koutoukidis, T: +44 (0)1865 617767 E: dimitrios.koutoukidis@phc.ox.ac.uk

2. LAY SUMMARY

In the UK 42,000 people each year are diagnosed with bowel cancer. It is the fourth most common cancer. Surgery to remove the cancer is the best treatment. However, it has a risk of complications, which is doubled for people with overweight/obesity. Patients experiencing complications recover more slowly, stay in hospital longer, and need more care. This isn't good for patients or the NHS.

Physical fitness and well-controlled blood sugar are linked with fewer complications from surgery. For people with overweight, weight loss improves both of these factors, so it may reduce complications. One reliable way to lose a meaningful amount of weight in the short period before surgery (3-4 weeks) is through a low-calorie diet programme: eating only special nutritious soups and shakes (800 calories/day) that have all the necessary vitamins. With weekly support from a dietitian, most people succeed. Typically, people lose 5% of their weight within 20 days. The NHS uses a version of this programme to treat type 2 diabetes.

In small-scale studies, patients with cancer and overweight have been willing and able to take part in less intensive weight management programmes before surgery, but lose little weight. However, the period before bowel cancer surgery is associated with feelings of uncertainty and anxiety, so it is unclear if patients can follow a more intensive programme.

To start to find out if this treatment is in the best interests of patients' physical and mental health, we will recruit 72 patients with overweight awaiting bowel cancer surgery. Half will be randomly allocated to continue with their usual care and half will be offered the weight loss programme. We will see whether enough patients are willing to take part, lose weight, and return for follow-up visits. We will monitor complications for 30 days after surgery and any reduction in muscle mass as a result of the weight loss. We will interview patients about their experience.

This information will tell us if a full trial is worthwhile to test whether this programme can reduce complications from surgery, improve outcomes for people with bowel cancer, and if the financial costs are likely to be worth the benefits. It will also help us refine the treatment plans according to patient feedback.

We discussed the study with 7 patient and public representatives. They thought it was an important study. We included their suggestions for making it easier for patients to join the trial, stick to the programme, and attend visits. We will work with them throughout the trial.

We will publish results in scientific journals and talk to clinicians and to patients with cancer supported by professional groups and charities (e.g., Macmillan). Our patient group will help us to explain the results clearly.

3. SYNOPSIS

Study Title	Pre-operative intentional weight loss to support post-operative recovery in patients with overweight and colorectal cancer: the CARE feasibility parallel randomised controlled trial
Short title	Could supported weight loss reduce bowel cancer surgery complications?
Study registration	ISRCTN39207707
Sponsor	University of Oxford<br>RGEA, Joint Research Office, Churchill Drive, Headington, Oxford OX3 7GB<br>T: +44 (0)1865 616480 E: rgea.sponsor@admin.ox.ac.uk
Funder	National Institute for Health Research for Patient Benefit
Study Design	Multi-centre feasibility parallel randomised controlled trial with embedded evaluation and optimisation of the recruitment process
Study Participants	Adults with overweight awaiting colorectal cancer surgery
Sample Size	72 (36 per arm)
Planned Study Period	Total trial length: 5.5 years<br>Individual participant's involvement: approx. 2-3 months<br>Long-term follow-up via medical records: up to 3 years
Planned Recruitment period	March 2023 to November 2024

Outcomes	Objectives	Outcome Measures	Timepoint(s)
Primary	1. To assess whether progression to a definitive RCT is justified	i. Recruitment rate<br>ii. Engagement rate<br>iii. Adherence rate<br>iv. Retention rate<br>v. Safety profile	i. Screening<br>ii. Throughout the intervention<br>iii. Throughout the intervention<br>iv. Pre-operative assessments and 30 days post-operatively<br>v. Throughout the trial
Secondary	To report between-group differences in<br>2. Morbidity<br>3. Oncological outcomes	i. Any morbidity<br>ii. Morbidity by grade (I, II, IIIa, IIIb, IVa, IVb)<br>iii. Survival (grade V)	i.-ii. Discharge and 30-days post-operatively<br>iii: Discharge, 30-days post-operatively, 3 years

Outcomes	Objectives	Outcome Measures	Timepoint(s)
		iv. Resection margins<br>v. Recurrence<br>vi. New primary/ secondary cancer	iv. Discharge<br>v-vi: 3 years
	4. Operative outcomes	vii. Intraoperative blood loss<br>viii. Operative time<br>ix. Conversion to open surgery	vii-ix: Discharge
		x. Surgical site infection<br>xi. Stoma rates & complications<br>xii. Radiologically - defined anastomotic leaks<br>xiii. Time in intensive care unit and high dependency unit	x-xiii: Discharge and 30-days post-operatively
		xiv. Re-operation rates<br>xv. Re-admission rates	xiv-xv: 30-days post-operatively and 3 years
	5. Hospital stay	xvi. Length of hospital stay (fitness to discharge)	xvi: Discharge
		xvii. Days alive and out of hospital	xvii: 30-days post-operatively
	6. Anthropometry	xviii. Weight<br>xix. Fat-free mass	xviii-xix: Baseline, pre-operative assessment 4, and 30 days post-operatively
	7. Fitness	xx. Time for sit-to-stand test	xx: Baseline, 30 days post-operatively
	8. Health-related quality of life (HRQoL)	xxi. EQ-5D-5L<br>xxii. HADS	xxi-xxii: Baseline, pre-operative assessment 3 and 30 days post-operatively
		xxiii. EORTC-QLQ-CR29	xxiii: 30 days post-operatively
	9. Costs and resource use	xxiv. Intervention costs	xxiv: End of intervention

Outcomes	Objectives	Outcome Measures	Timepoint(s)
	10. Adverse events	xxv. Healthcare resource use<br>xxvi. QALYs<br>xxvii. Adverse events	xxv-xxvi: Baseline, 30 days post-operatively<br>xxvii: Baseline, pre-operative assessment and 30 days post-operatively
Process	To examine the<br>1. Experience of the intervention<br>2. Experience of the trial<br>3. Control group contamination<br>4. Fidelity of delivery<br>5. Barriers to trial enrolment	i. Analysis of qualitative interviews with intervention participants<br>ii. Feedback pre-operatively<br>iii. Feedback post-operatively<br>iv. Feedback post-operatively<br>v. Analysis of QuinteT qualitative interviews with participants and staff<br>vi. Analysis of audio-recorded recruitment appointments<br>vii. Feedback post-operatively<br>viii. Observation of consultations<br>ix. Reasons for declining participation	i. Pre-operative assessment 2<br>ii. Pre-operative assessment 3<br>iii. 30-days post-operatively<br>iv. 30 days post-operatively<br>v: Pre-operative assessment 1 and throughout the trial<br>vi. Screening<br>vii: 30 days post-operatively<br>viii: Throughout the intervention<br>ix. Screening
Intervention(s)	Low-energy total diet replacement programme with behavioural support		
Comparator	Care as usual		

4. ABBREVIATIONS

ASA	American Society of Anaesthesiologists
BMI	Body mass index
CI	Chief Investigator
CRC	Colorectal cancer
eCRF	Electronic Case Report Form
eGFR	Estimated glomerular filtration rate
EORTC-QLQ-C30	European Organisation for Research and Treatment of Cancer Core Quality of Life Questionnaire
EORTC-QLQ-CR29	European Organisation for Research and Treatment of Cancer Quality of Life Questionnaire – Colorectal cancer module
EQ-5D-5L	EuroQoL-5D 5-level version
GCP	Good Clinical Practice
GP	General Practitioner
HADS	Hospital Anxiety and Depression Scale
HRA	Health Research Authority
HRQoL	Health-related quality of life
ICF	Informed Consent Form
NHS	National Health Service
NIHR	National Institute for Health and Care Research
RES	Research Ethics Service
PI	Principal Investigator
PIS	Participant Information Sheet
RCT	Randomised controlled trial
R&D	NHS Trust R&D Department
RGEA	Research Governance, Ethics & Assurance Team
REC	Research Ethics Committee
SAP	Statistical Analysis Plan
SOP	Standard Operating Procedure
QALYs	Quality-adjusted life years

5. BACKGROUND AND RATIONALE

Colorectal cancer (CRC) is the fourth most common cancer in the UK. More than 42,000 people are diagnosed annually. Surgery is the standard treatment for ~70% of patients (n=29,000) but leads to significant post-operative morbidity. This morbidity increases the psychological and health burden of patients by a factor of 10.[1] It also typically doubles healthcare spending.[2]

Concomitant overweight, and especially obesity, independently doubles the morbidity risk following CRC surgery (43% vs. 21% without obesity).[3] Two third of patients with CRC have overweight (with a third of patients with CRC having obesity) at diagnosis.[4] Systematic reviews with meta-analyses show that obesity (compared with no obesity) is associated with an additional day of hospital stay, a 20-minute longer operation,[3] serious post-operative complications (21% vs. 15%),[5] anastomotic leaks (RR: 3)[6] and double rates of conversion to open surgery regardless of demographic characteristics.[7]

Linked to the rise in overweight and obesity, CRC cases are projected to increase by 30% between 2020 and 2040, further increasing the burden for patients and the NHS.[8, 9]

Guidelines for any pre-operative treatment are weak due to low-quality evidence.[10, 11] In two James Lind Alliance priority setting partnerships (for peri-operative care and for people living with cancer), finding effective pre-operative treatments and preventing surgical complications were among the most important research questions.[12, 13]

Pre-operative intentional weight loss in patients with overweight awaiting CRC surgery could reduce post-operative morbidity by improving physical function, cardiovascular fitness, systemic inflammation, and glucose regulation.[14-19] The amount of weight loss needed to improve morbidity outcomes in other conditions follows a dose-response pattern. Evidence drawn from bariatric surgery studies consistently shows that 5-9% and ≥10% pre-operative weight loss is independently associated with 31% and 42% lower 30-day mortality, respectively.[20]

In the CRC setting, weight loss needs to be achieved within the typical 4-week window between decision to treat and surgery. The most reliable and scalable way to achieve this is through a nutritionally-replete, high-protein, low-energy total diet replacement programme with behavioural support (TDR). TDR reliably leads to a mean 7% (SD: 1.8kg) weight loss within 4 weeks in diverse populations with obesity-related diseases, including in older adults with moderate frailty and with atrial fibrillation, and implemented in pragmatic settings.[21-30] A longer version of the programme is currently being tested nationally in the NHS with the aim of remission of type 2 diabetes.[31]

Intentional weight loss is strongly linked with intervention adherence.[32] However, the period around cancer diagnosis is associated with feelings of uncertainty and anxiety.[33, 34] In this context, it is unclear if people with cancer will enrol and adhere to this intensive intervention to the same extent as in less uncertain chronic disease settings. On the other hand, the structured nature of a nutritionally replete dietary intervention may give people a sense of control and empowerment.[35, 36] Patients report their cancer diagnosis being a stimulus for healthier dietary change,[37, 38] but also report making only marginal changes on their own.[39] This highlights the need for support. Small single-arm and randomised trials have shown high feasibility of recruitment (~50%), engagement (~90%), and retention (~85%) to less intensive pre-operative dietary weight loss interventions in breast, prostate, and gastric cancers. These programmes advised an energy-restricted healthy diet or provided partial meal

replacements.[40-43] Whilst these approaches support the feasibility of intervening in the pre-operative cancer setting, they achieved only small weight loss (average: 3kg) with high variability (SD: 4-5kg) that may be insufficient to improve surgical outcomes.

Contrary to this evidence of intentional weight loss in structured programmes, evidence from cohorts suggests that pre-operative weight loss is associated with worse post-operative and long-term outcomes. However, this is most likely to be unintentional weight loss and explained by selection bias due to advanced stage disease.[44]

There are also theoretical concerns about muscle mass loss. However, the amount of body fat is positively associated with the amount of muscle mass.[45, 46] During *intentional* weight loss, muscle mass reductions are small (~1%),[47, 48] likely not clinically meaningful,[49, 50] and, in older adults, weight loss significantly improves physical and cardio-metabolic fitness.[14, 51] Another trial of very low energy diet in older adults aged 65-85 years showed improvements in physical function without adverse outcomes despite small reductions in lean mass.[29] We have also replicated these changes in body composition in our ongoing trial of TDR in patients with obesity and advanced liver disease, who are moderately frail, with no adverse outcomes.[26] Unlike some weight loss programmes, TDR, being micronutrient-rich, can improve general nutritional status,[29, 52] which could contribute to beneficial outcomes.[53, 54]

Accordingly, pre-operative TDR may improve outcomes in this population but this hypothesis needs formal testing. A pilot trial with a nested qualitative component is required to estimate recruitment, engagement, adherence, and retention before a trial testing the intervention's effectiveness and cost-effectiveness can be realised.

Adoption of effective interventions into practice depends critically on cost-effectiveness. Typically, prehabilitation before cancer surgery has involved logistically complex and resource-intensive interventions, such as inclusion of multi-disciplinary services, multiple face-to-face appointments, need for space and equipment in hospitals, and significant travelling to hospitals by patients. From a health services and social services perspective, such interventions would need to be very effective in reducing post-operative complications to be cost-effective. Currently, no such intervention has met these criteria.

The relatively low cost of a 4-week TDR programme [total approx. £440/patient comprising £240 for dietetic support, £200 for food products based on previous cost-effectiveness analyses][55, 56] compared with the average cost of post-operative morbidity in CRC (£7.5k/patient) might make this intervention cost-effective. We will estimate potential costs and benefits during the pilot and determine the necessary resources (e.g., intensity of behavioural support) to guide the future definitive trial.

For the potential of the pilot trial to be realised, recruitment to target is key. Complex logistics and heavy participant burden have been recruitment challenges in some previous RCTs on prehabilitation. Although these will be reduced by testing an intervention delivered remotely, some may remain. For example, some clinicians and recruiting staff may have preconceptions about this intervention, such as potential mistrust of rapid weight loss diets in general or lack of confidence in weight loss as a suitable treatment.[57, 58] This may influence whether and how they present the trial to the patients. The way patients respond when presented with the offer of a weight loss programme is also crucial and hard to predict. Their decision may be complicated by an awareness that weight loss is a symptom of advanced cancer and not perceived as an established treatment, or patients may perceive

the control arm as "no treatment" and refuse randomisation. The recruitment process will require excellent coordination and good communications between doctors, research and clinical nurses, dietitians, and patients. To mitigate these potential challenges, we will use the established QuinteT process.[59] This iterative and cumulative process will allow us to understand the recruitment as it happens and iteratively develop and test ways to address identified challenges. The QuinteT process has increased recruitment in RCTs with similar challenges.[60]

6. OBJECTIVES AND OUTCOME MEASURES

Please refer to objectives and outcomes in section 3.

7. STUDY DESIGN

This is a prospective randomised controlled trial (RCT) with an embedded evaluation and optimisation of the recruitment process (QuinteT) to assess the feasibility of progression to a definitive RCT. Participants will be recruited from hospitals across England.

Participants are expected to be involved in the study for approximately 2-3 months. They will be asked to attend hospital visits for screening, pre-operatively (on the day of surgery), and 30 days post-operatively. They will also remotely complete questionnaires at 1-3 days pre-operatively and have a semi-structured qualitative interview over the phone. The intervention will be delivered over the pre-operative period. Appendix A shows the study flow-chart.

8. PARTICIPANT IDENTIFICATION

8.1. Study Participants

Participants with BMI ≥ 28 kg/m^2 listed for colorectal cancer surgery aged ≥ 18 years.

8.2. Inclusion Criteria

- Participant is willing and able to give informed consent for participation in the study.
- Able to communicate in English or has a relative/friend/carer acting as interpreter.
- Aged 18 years or above.
- BMI ≥ 28 kg/m^2 (or BMI ≥ 25 kg/m^2 for people of Black, Asian, or minority ethnic origin).
- Listed for curative elective colorectal resection for cancer.
- If neoadjuvant treatment is indicated, it must have been completed.
- Performance status 0-2.

8.3. Exclusion Criteria

The participant may not enter the study if ANY of the following apply:

- ≥10% self-reported weight loss in the 6 months before the screening visit

- <20 days from the screening visit until surgery.
- Having allergy to soy.
- Documented stage 4-5 kidney disease.
- Documented severe heart failure (defined as New York Heart Association grade 3 or 4).
- Previous bariatric surgery.
- Type 1 diabetes.
- Currently on warfarin.
- Currently on insulin with a previous episode of diabetic ketoacidosis.
- Radiological suspicion of imminent intestinal obstruction or endoscopic evidence of an impassable tumour.
- Pregnancy, breastfeeding, or planning pregnancy during the course of the trial.
- Any other significant disease or disorder which, in the opinion of the Investigator or healthcare professional, may either put the participants at risk because of participation in the trial, or may influence the result of the trial, or the participant's ability to participate in the trial.
- Currently taking part in other interventional clinical trials unless agreed in advance by all trial teams (participation in observational studies is allowed).

A list of trials that co-enrolment has been agreed by all trial teams and a list of trials that co-enrolment has been agreed to not be allowed will be regularly updated and provided to trial sites.

9. PROTOCOL PROCEDURES

A schedule of procedures is available in Appendix B.

9.1. Recruitment

Participants will be recruited from NHS Trusts across England. We will aim to select sites to cover multiple geographical areas. Sites need to meet the following criteria:

- Assign a named healthcare professional (surgeon, anaesthetist, nurse, or dietitian) as local Principal Investigator
- Train staff to the trial procedures
- Have adequate staff and resources to
  - recruit participants, conduct study assessments, and provide relevant research data (including a blinded researcher for outcome assessment)
  - liaise regularly and as appropriate with the Surgical Intervention Trials Unit and central study team, and
  - commit to a minimum target recruitment rate of 3 patients every 4 months (0.75 patients per month).

The recruitment pathway will be flexible within and across recruitment sites to allow for differences in cancer diagnostic and treatment pathways across patients and hospitals. In all cases, a

member of the clinical team will be the first person who will raise the option of the research study to a potentially eligible participant before that participant is formally approached by a member of the research team to discuss entering the trial.

Patients may be approached about the trial before or after the final decision to have surgery has been made to allow for as much time as possible for potential participants to consider participation. However, they will only enrol after they have been (provisionally) listed for surgery. For example, when cancer is suspected during routine checks, a member of the clinical team (e.g., the cancer nurse specialist or doctor) will inform the patient about the possibility of a cancer diagnosis and the likelihood of surgery. At this time, they will make patients aware of the trial and ask them for their verbal consent to pass their contact details to the research team. The member of the clinical or research team will provide the potential participant with a participant information sheet (PIS). A research nurse (or other member of the research team) will contact the participant to discuss the study and pre-screen potentially eligible participants with a brief phone interview to check the eligibility criteria and concomitant medication. Once the CRC diagnosis is confirmed, a research nurse (or member of the clinical or research team) will contact the potential participant for a second discussion about the trial and to book them in for a screening visit. In other cases, the first contact of a potential participant with the research nurse might be after the CRC diagnosis has been confirmed by the clinical team or after potential participants have discussed the surgical treatment options with a member of the clinical team and a decision to treat has been made.

In hospitals where participants are notified in advance that research studies are taking place and their personal information may be accessed by the research team, a research nurse (or member of the research team) may also screen relevant clinic lists and/or observe the multidisciplinary team meeting where surgical cases are discussed to flag potentially eligible participants to the clinical team.

We will create a video covering the key points of the PIS, upload it online, and direct participants to the video link to facilitate accessibility.

For colon cancers, the screening visit should be booked within the next working day (and up to 3 days) following communication to patient they have been (provisionally) listed for surgery.

For rectal cancers (<20cm from the anal verge), the decision to book the screening visit will be made following the multidisciplinary team meeting. If neoadjuvant treatment is indicated, the participant should be booked for a screening visit only following completion of neoadjuvant treatment. If neoadjuvant treatment is not indicated, the participant should be booked for a screening visit within the next working day (and up to 3 days) following communication to patient they have been (provisionally) listed for surgery.

Participants who decline to participate will be asked to provide the reasons for declining to take part by choosing all possible reasons from the following pre-defined list. This information is likely to be provided over the phone to the member of the clinical or research team and will be retained for the study without any personal identifiers (anonymously).

1. I would find it difficult to stick to the diet
2. I do not like the idea of eating shakes and soups instead of usual food
3. I am worried about potential side effects from the diet
4. Transport / distance to hospital

5. I feel unable to cope with additional requirements of me at this moment in time
6. I do not like the idea of randomisation
7. I feel uncertain that the trial will benefit me
8. I was not given adequate information
9. Other

9.2. Informed Consent

The participant enrolling on the trial must personally sign and date the latest approved version of the Informed Consent form before any study specific procedures are performed.

Written and verbal versions of the participant information and informed consent will be presented to the participants detailing no less than: the exact nature of the study; what it will involve for the participant; the implications and constraints of the protocol; the known side effects and any risks involved in taking part. It will be clearly stated that the participant is free to withdraw from the study at any time for any reason without prejudice to future care, without affecting their legal rights, and with no obligation to give the reason for withdrawal.

The participant will be allowed as much time as wished to consider the information, and the opportunity to question the Investigator, their GP or other independent parties to decide whether they will participate in the study. Electronic written informed consent will then be obtained by means of electronic participant-dated signature and dated signature of the person who presented and obtained the informed consent on the eCRF. The person who obtained the consent must be suitably qualified and experienced and have been authorised to do so by the Chief/Principal Investigator. A copy of the signed informed consent will be given to the participant (via email or printed physical copy). The original signed form will be retained in the eCRF at the study site.

For consent to audio-recordings of the recruitment interactions, please refer to section 9.6.5.b and to Appendix C.

9.3. Screening and Eligibility Assessment

During the screening visit, participants will provide written informed e-consent. Following informed consent, their weight and height will be measured to ensure they meet the BMI criterion. The concomitant medication will be reviewed. Each participant must satisfy all the approved inclusion and exclusion criteria of the protocol. If a participant does not meet all of the criteria, they will be recorded as a screen failure. Participants will also complete all baseline assessments.

9.4. Randomisation

The randomisation software will be programmed by the clinical trials unit. There will be no paper-based back up randomisation procedure in case of emergencies.

At the end of the screening visit, participants who meet all the eligibility criteria and are keen to proceed with the study should be randomised. Queries on eligibility must be resolved before randomisation and participants who do not meet all the eligibility criteria must not be randomised. The research nurse or delegate will randomly allocate participants using a web-based central minimisation software. Eligible participants will be individually randomised with a 1:1 allocation ratio to receive either

the intervention or care as usual through minimisation with a 20% random element. The two stratified variables will be performance status (0 vs 1-2) and medPerian age at diagnosis (($</\geq$70 years).

The research nurse will enter the participant details to the randomisation system (initials, participant ID and research site code, confirmation of eligibility, confirmation of completion of baseline assessments, date of informed consent, and stratification factors). The system will allocate the participant and instantly inform the researcher of the allocation. The researcher will then inform the participant of their allocation.

The maximum duration between completion of the screening visit and randomisation will be 24 hours (e.g., to allow for query resolution). In such case, the local research team will contact the participants to notify them about their allocation.

If randomisation has not occurred within 24 hours but participants are still keen to continue with the study, they will have to be re-screened by checking that the time limit until surgery has not elapsed but participants will not be required to complete again the baseline assessments.

Allocation concealment is achieved as randomisation occurs after the baseline visit, the randomisation algorithm is unmodifiable and concealed from investigators and the local research teams, and the local research teams have no access to the total number of participants randomised to each group.

Following randomisation, the research team will send a letter to the participant’s GP informing them about trial participation and group allocation.

9.5. Blinding and code-breaking

It is impossible to blind the participants and research nurses due to the nature of the intervention. Therefore, procedures for breaking the allocation code are not applicable. However, the assessors of the future primary outcome (research nurses conducting the post-operative follow-up visit) will be blinded.

9.6. Description of study intervention(s), comparators and study procedures (clinical)

9.6.1. Description of study intervention(s)

The intervention is a low-energy total diet replacement programme with behavioural support.

Participants will replace all their foods with a nutritionally complete package of 4 formula products per day (such as soups and shakes (~800kcal/day)). The composition will follow regulatory guidelines.[61]

They will be advised to drink >2.5L/day of energy-free fluids (e.g., water, tea, coffee, diet soft drinks) with a provision of up to 100ml of skimmed milk for tea/coffee per day but no energy-dense drinks (e.g., alcohol).

Participants will have a 45-min introductory phone call with a dietitian to provide behavioural support and then weekly 20-minute follow-up calls. Participants will be offered the option of having the

support over video (MS Teams) if they prefer. The support aims to maintain motivation during the adjustment to formula foods and problem-solve issues that arise. This is a structured programme using weekly progress review, feedback on changes, checks on product tolerance, practical tips for mixing shakes and adding flavour, problem solving any barriers, managing social situations, coping with hunger, encouragement, action planning, avoiding and managing lapses, and behaviour change techniques to maintain motivation. In previous studies the support has been valued highly by participants.[36, 62]

The intervention will start on the day post-randomisation. Following randomisation, research nurses will provide participants a few products, so that they can start the programme the next day. The introductory phone call with the dietitian should ideally be completed on the day of or the day following randomisation. Participants will receive the rest of the products by post.

The intervention will finish approximately two days pre-surgery (depending on local guidance for standard pre-operative preparation) or when 15% weight loss has been achieved (if unexpected lengthy delays to schedule the surgery occur), whichever is earlier.

One in 5 people experience an adverse, mostly mild, event due to the intervention.[21] Constipation (1 in 7), fatigue (1 in 12), headache (1 in 17), and dizziness (1 in 22) are the most common Adverse events (AEs) albeit mild (only 11% were moderate or severe), reduce in intensity over time and disappear as soon as the intervention is discontinued.[21, 22] Here, the risk of constipation will be proactively managed with advice for high energy-free fluid intake (>2.5 litres/day) and a fibre supplement.

Participants will receive the intervention on top of the local standard care pathway that may include advice and support on pre-habilitation.

Medication adjustment

Type 2 diabetes: As per the NHS England and NHS Improvement guidance for this intervention, participants who are managing their diabetes with sulfonylureas (gliclazide, glibenclamide, glimepiride), meglitinides (Repaglinide, Nateglinide), or SGLT2 inhibitors (dapagliflozin, canagliflozin, empagliflozin, ertugliflozin) will be instructed to stop them on the first day of the intervention due to safety risks, including the risk of hypoglycaemia (sulfonylureas, meglitinides) or risk of ketoacidosis (SGLT2 inhibitors). If participants do not stop these medications, they must not start the intervention. Participants will be required to continue attending their diabetes review appointments /monitoring at their GP as usual and to notify their GP if they disengage from the intervention (as medication may need to be restarted).

Hypertension: If the blood pressure at the screening visit is uncontrolled (If blood pressure is considered uncontrolled at time of referral (systolic ≥ 140mmHg OR diastolic ≥ 90mmHg), no changes to blood pressure lowering medications will be made.

If the blood pressure at the screening visit is controlled (both systolic <140mmHg AND diastolic <90mmHg), one blood pressure medication should be adjusted on the first day of the intervention. Medication being used specifically and solely for managing blood pressure, in a particular participant, are the priority for adjustment. The agent that has been added last according to current NICE (National Institute for Health and Care Excellence) guidance should be stopped. If not being used for other indications, this would be (in order of stopping first):

a. Spironolactone or alpha blocker or beta blocker
b. Thiazide diuretic (or calcium channel blocker)
c. Calcium channel blocker (or thiazide diuretic)
d. Angiotensin-converting enzyme inhibitor or Angiotensin receptor blocker

If the patient is taking medications which affect blood pressure but ALL are being used for other indications (i.e., none are being used solely to manage blood pressure), clinical judgement and shared decision making should be used taking into account the blood pressure reading. In this case, the dose should be cautiously reduced (e.g., to the next lower dose) instead of stopped.

Self-monitoring of blood pressure and blood glucose

Intervention participants taking insulin or medication for hypertension will be asked to self-monitor their blood glucose or blood pressure, respectively, and report out of range values to the dietitian who will communicate this to the participant's GP within 3 working days (or earlier if deemed necessary). Participants will be counselled about symptoms of hypoglycaemia and postural hypotension and advised of when and how to seek appropriate support. They will receive blood pressure/glucose monitors if they do not own one.

9.6.2. Description of comparator

Participants will follow the local standard care pathway that may include advice and support on pre-prehabilitation.

9.6.3. Cancer waiting times

NHS Cancer Waiting Times Monitoring Dataset Guidance gives strict requirements for time to first definitive treatment date for curative colorectal surgery. As per the latest guidance and another national pre-habilitation trial in the field, the date of consent given by a participant to be entered into the trial will be the first definitive treatment date for this purpose in line with the above guidance [63, 64]. The pre-operative intervention part of this study has been designed to fit within the targets of cancer waiting times guidance. Surgery should not be unduly delayed as a result of entry into the study.

9.6.4. Description of study procedure(s)

Demographic questionnaire – 2 mins

Basic demographic characteristics including date of birth, sex, and ethnicity will be captured using standardised questions. Contact details (home address, contact number(s), GP practice address, next of kin) will be extracted from medical records and verified by the patient.

EuroQoL-5D 5-level version (EQ-5D-5L) questionnaire – 5 mins

Widely used and validated general HRQoL instrument assessing mobility, self-care, usual activities, pain/discomfort and anxiety/depression as well as overall self-rated health.

European Organisation for Research and Treatment of Cancer Quality of Life Questionnaire – Colorectal cancer module (EORTC-QLQ-CR29) – 10 mins

Widely used and validated CRC-specific HRQoL instrument assessing CRC-specific functional status and symptoms post-operatively.

Hospital Anxiety and Depression Scale (HADS) – 5 mins

Widely used and validated anxiety and depression questionnaire.

Resource use questionnaire – 5 mins

A modified Client Service Receipt Inventory [65] will be used to collect data on use of primary care services, social care services, and other health care professional, as well as time off work.

Concomitant medication (5 min)

Participants will be asked to bring their prescription or a list of their medication at the screening visit for these to be recorded.

Weight and body composition – 3 mins

Weight will be measured barefoot and with light clothing to the nearest 0.1kg using a calibrated digital scale which will also estimate body composition using bioelectrical impedance.

Height – 2 mins

Height without shoes will be measured to the nearest 0.1cm using a stadiometer.

5 times sit to stand test – 3 mins

Participants sit in a standard straight-backed stable chair (without wheels) positioned by a wall. They then stand fully and sit back down, without using the hands, five times, as quickly as possible.

Post-operative complications

Presence and type of any morbidity. This will be based on medical records for complications during the hospital stay and on participants reporting it using a standard proforma for complications following discharge. They will be graded (as I-V) independently by two researchers blinded to treatment allocation with the Clavien-Dindo classification of post-operative complications, the most widely used and validated measure.[66]

Table 1: Clavien-Dindo classification of post-operative complications

	Morbidity grade definition
I	Any deviation from the normal postoperative course without the need for pharmacological treatment or surgical, endoscopic, and radiological interventions.<br>Allowed therapeutic regimens are: drugs as antiemetics, antipyretics, analgesics, diuretics, electrolytes, and physiotherapy.<br>Grade I also includes wound infection opened at the bedside.
II	Requiring pharmacological treatment with drugs other than those allowed for grade I complications.<br>Grade II also includes blood transfusions and total parenteral nutrition.

III	Requiring surgical, endoscopic, or radiological intervention.
IIIa	- Not under general anaesthesia
IIIb	- Under general anaesthesia
IV	Life-threatening complication (including central nervous system complications) requiring intensive care unit management
IVa	- Single organ dysfunction
IVb	- Multiorgan dysfunction
V	Death

Adverse events (AEs)

Patient will self-report potential adverse events through a pre-defined list of expected adverse events based on previous trials of this type of intervention as well as free-text for any additional adverse event.

Fitness to discharge

This will be extracted from medical records and be used to calculate the length of hospital stay and, together with survival, the days alive and out of hospital.

Operative outcomes

These will be extracted from medical records.

At discharge:

- *Intraoperative blood loss, in mL*
- *Operative time, in minutes*
- *Conversion to open surgery (yes/no)*

At discharge and 30 days post-operatively:

- *Surgical site infection*
- *Rate of stomas and stoma-specific complications*
- *Presence of radiologically-defined anastomotic leaks*
- *Time in intensive care unit and high dependency unit*

At 30 days post-operatively and at 3 years:

- *Re-operation rate*
- *Re-admission rate*

Descriptive data on the initial operation approach, operation type, and grade of surgeon performing the operation, will also be extracted.

Oncological outcomes

These will be extracted from medical records

At discharge:

- *Resection margins, in millimetres*

At discharge, 30 days post-operatively, and 3 years:

- *Survival*

At 3 years:

- *Recurrence*
- *New primary/secondary cancer*

Descriptive data on tumour histological staging and standardised data for the National Bowel Cancer Audit will also be extracted.

Feedback questionnaire pre-surgery by intervention participants – 2 mins

This 8-item questionnaire will assess intervention acceptability using adapted questions from the Theoretical Framework of Acceptability questionnaire.[67]

Feedback questionnaire post-surgery – 5 mins

This 11-item study-specific questionnaire will assess satisfaction with trial processes, potential contamination of the usual care group, and satisfaction with the intervention for the intervention participants.

QuinteT qualitative interviews to evaluate and optimise recruitment – optional – ~20min

To explore experience of being approached, factors influencing trial participation, understanding and acceptability of the presence of chance in random allocation, comparison of the two naturally different trial groups, and perceived acceptability, benefits, and risks from being randomised to each group. All participants who consent to the main trial will be invited for this optional phone interview regardless of whether they accept or reject their allocation (e.g., do not start the intervention). This interview should occur where possible within 4 days of providing informed consent for the trial. The topic guide (Appendix D) may be refined as the study progresses, as per best practice. Interviews will be conducted over the phone/MS Teams, will be audio-recorded, and transcribed verbatim. For details on the QuinteT process, see 9.6.1.

Qualitative interviews with intervention participants – ~45min

Interviews will invite views on (a) the delivery of the programme, (b) barriers to engagement, (c) barriers to adherence, (d) facilitators to adherence while providing flexibility to participants to discuss their views in their own way highlighting aspects of experience that were particularly important to them. They will also capture participants' views on changes in physical activity. The sample size should allow saturation to be reached.[68] Interviews will also cover gastrointestinal symptoms relevant to both CRC and the intervention (e.g., constipation). The topic guide (Appendix E) may be refined as the study progresses, as per best practice. Interviews will be conducted over the phone/MS Teams, will be audio-recorded, and transcribed verbatim.

Qualitative interviews with research and clinical staff – ~45min

These interviews will aim to explore understanding of trial rationale, design, equipoise, perceptions of the intervention, whether staff would (or not) discuss the trial based on criteria beyond the

inclusion/exclusion criteria, concerns, perception of patients' reactions to the trial, recruitment procedures, and relevance of results to their own practice. The topic guide (Appendix F) may be refined as the study progresses, as per best practice. Interviews will be conducted over the phone/MS Teams, will be audio-recorded, and transcribed verbatim.

Fidelity of delivery

A researcher not delivering the intervention will observe a random 10% subsample of the initial dietetic consultations and a 10% subsample of the subsequent dietetic consultations (i.e., join the phone/video call) as they occur and code them with pre-specified criteria to assess fidelity of delivery.

9.6.5. QuinteT evaluation and optimisation of the recruitment process

QuinteT Step 1: Understand recruitment as it happens

Design and aim: Qualitative analysis to understand recruitment challenges (e.g., how to present the trial and address patient concerns) to inform how to optimise recruitment processes.

a. Semi-structured telephone interviews with patients (~20min, optional) and local study staff (~45min).

Participants: Participants who consent to the trial will be offered the optional QuinteT interview as per 9.6.4.

Study staff: Local study staff [CRC surgeons (n~=6), cancer nurse specialists (n~=6), research nurses (n~=8)] will also be provided with interview-specific participant information sheets by the central study team. Informed consent will be in line with section 9.2. The objectives will be in line with 9.6.4.

b. Audio-recording of recruitment interactions about the trial (as per 9.1) between patients and local study staff (n~=20).

The aim of analysing these data is to enrich the interview data above and reduce potential social desirability and recall bias. They will be used to explore study information provision, recruitment techniques, patient concerns, and randomisation decisions to identify recruitment difficulties and improve information provision.

As per 9.1, patients will be referred to a member of the clinical or research team (e.g., research nurse) to discuss the trial. These interactions may take place face to face or over the phone. Any such interaction in which the trial is discussed with potential participants up until their study participation decision (this includes the screening (first study) visit and interactions prior to this) will, with verbal permission, be audio-recorded.

At the completion of the interaction, the patient will be booked in for a screening visit or may decline to take part. Those coming for a screening visit will be asked to provide informed consent for the analysis and storage of the audio-recording of their face-to-face screening visit. If they decline to take part in the trial, the patient will be asked whether they are happy for the audio-recorded interaction to be kept and analysed or whether they prefer that it gets deleted. If they decide that they don't want the audio-recording kept, this will be deleted. If they are happy for the audio-recording to be kept and analysed, participants will need to provide informed consent only for this aspect of the study using the "Consent form only for audio-recording conversations about the CARE study."

- If the interaction occurs over the phone, the member of the research team (e.g., research nurse) will read through the consent form statements to the participant and complete an electronic form on behalf of the participant. The researcher will then electronically sign the form and send it to the participant (via email or by post), so that the participant can keep it for their records.
- If the interaction occurs face to face, the participant will sign the consent as per 9.2.

Audio-recordings will only be transferred to the central study team after informed consent has been obtained.

c. Assessment of screening logs

The local study teams will provide fully anonymised screening logs to the central study team. These logs will be assessed using the SEAR (screening, eligibility, approach, and randomisation) framework for identification of screen failures and dropouts.[69]

Script development: The analysis of the above will culminate in developing a "script", aiming to change problematic terms, so that equipoise communication is improved and common concerns are addressed.

QuinteT Step 2: Feedback and script piloting

Design and aim: Interviews and audio-recordings of the recruitment interactions to determine how the trial is communicated when the script is used and identify necessary iterations.

Process: Recruiters (research nurse or member of the clinical/research team) will receive the script and feedback. The recruiter will audio-record the recruitment interaction with the patients while explaining the trial using the script. As per Step 1, patients and local study staff will also be invited for an interview.

Sampling, recruitment, and consent: Interviews with a convenience sample of patients (n~=10) and local study staff (n~=6) at 3 sites (but purposively selecting sites with lower initial recruitment rates) to allow for rapid improvements. Recruitment and consent will be in line with Step 1.

Objectives and analysis of interviews: As per step 1. For staff, interviews will also explore how comfortable they feel using the script.

Objectives and analysis of audio-recordings of the recruitment interactions (n~=10): Following the above methods, this analysis will explore how well recruiters follow the script, communication challenges, and clear words and phrases to explain equipoise without inadvertently showing preferences. Overall, together with examination of screening logs, this will aim to identify whether the script improves recruitment rates in a pre-post analysis.

Script iteration: Based on the above findings, the script will be revised and recirculated with feedback.

QuinteT Step 3: Optimising recruitment

Design and aim: Analysis of audio-recordings of the recruitment interactions in all sites during months 9-16 to fully optimise recruitment and develop a recruitment strategy for the main RCT.

Sampling, recruitment, consent, and analysis: As per steps 1-2 with a convenience sample of n~=6 audio-recorded recruitment interactions from each site (to explore differences by site) aiming for analysing n~=35 interactions.

QuinteT Step 4: Facilitating enrolment

Design and aim: Semi-structured qualitative interviews (~20min) to identify challenges in starting the intervention in time together with inspection of logs recording time period between randomisation and intervention commencement.

Interviewees: Research nurses at each site (n=6) and the dietitians (n=2) delivering the intervention.

Objectives: To explore individual and structural factors influencing rapid implementation of the baseline visit, randomisation, and intervention commencement.

Sampling, recruitment, consent, and analysis: As per step 1.

9.7. Baseline Assessments

The baseline face-to-face assessment will be conducted during the screening visit at each site. It will last approximately 1.5 hours and it will include:

- Demographic questionnaire
- Concomitant medication
- EQ-5D-5L questionnaire
- HADS questionnaire
- Resource use questionnaire
- Height
- Weight and fat-free mass
- 5 times sit to stand test

9.8. Subsequent Assessments

The study schedule is available in Appendix B. The window periods will be:

- 5 days for the optional pre-operative follow-up assessment 1 (0 to 4 days post-randomisation)
- 7 days for the pre-operative follow-up assessment 2 (3 days before the time the interview is expected to occur to 3 days after that)
- 4 days for the pre-operative follow-up assessment 3 (From the day of the assessment is expected to occur (4 days pre-operatively) to the day of surgery
- Between admission and surgery for the pre-operative follow-up assessment 4
- 27-37 days post-operative for the post-operative follow-up assessment scheduled 30 days post-operative
- 3-year follow-up from medical records: 3 years ± 9 months post-operatively

<u>Pre-operative follow -up assessment 1 – all participants (optional)</u>

Optional QuinteT qualitative interview with all patients who consent to the main trial.

<u>Pre-operative follow-up assessment 2 – only for participants in the intervention group</u>

Qualitative interviews with intervention participants. Interviews will occur approximately halfway through the programme, so that participants are well into the intervention but not too close to surgery to better capture the whole pre-operative experience.

Pre-operative follow-up assessment 3 – all participants

This will include the EQ-5D-5L and HADS questionnaires for all participants. In addition, it will include the feedback pre-surgery questionnaire for intervention participants only. Participants will choose to receive either a link to fill in the questionnaires electronically or a paper questionnaire that they can post or bring in clinic on the day of surgery. Participants will receive email and/or phone reminders to facilitate questionnaire completion

Pre-operative follow-up assessment 4 – all participants

This will include weight and fat-free mass.

Post-operative follow-up assessment – all participants

Where possible, this face-to-face assessment will occur jointly with the standard clinical follow-up appointment to minimise travel. It will include:

- Post-operative complications reporting
- EQ-5D-5L questionnaire
- EORTC-QLQ-C29 questionnaire (only completed post-operatively as many questions are not relevant pre-operatively)
- HADS questionnaire
- Resource use questionnaire
- Weight and fat-free mass
- 5 times sit to stand test
- Adverse events
- Post-surgery feedback questionnaire

Staff assessment

A purposive subsample of research and clinical staff will be invited for a 20-minute qualitative interview. Staff will be given an information sheet and provide informed consent prior to interview.

Long-term follow-up through the hospital medical records

We will extract relevant data collected as part of routine care at each study site for up to 3 years after each participant completes the study. The type and timing of relevant data collected will depend on data availability based on each participant's routine clinical care. This is likely to include weight, morbidity, mortality, disease progression, new diagnoses, medication, re-admission, re-operation, and healthcare resource use.

9.9. Sample Handling

No samples will be taken.

9.10. Early Discontinuation/Withdrawal of Participants

During the course of the study a participant may choose to withdraw early from the intervention at any time. This may happen for several reasons, including but not limited to:

- The occurrence of what the participant perceives as an intolerable AE.
- Inability to comply with study procedures
- Participant decision

Participants in the care as usual group: They may opt out of study assessments but may remain on study follow-up.

Participants in the intervention group: They may stop the intervention and/or study assessments but may remain on study follow-up and will be encouraged to do so.

All participants may also withdraw their consent, meaning that they wish to withdraw from the study completely. In the case of withdrawal from both treatment (i.e., intervention) and active follow up, the following options for a tiered withdrawal from the study will be given to participants and explained in the participant information sheet.

According to the design of the study, option 1 below will be the default. Alternatively, participants can explicitly opt for option 2.

1) Participants may withdraw from active follow-up and further communication but allow the study team to continue to access their medical records and any relevant hospital data that is recorded as part of routine standard of care; i.e., CT-Scans, blood results and disease progression data etc.

2) Participants can withdraw from the study but permit data obtained up until the point of withdrawal to be retained for use in the study analysis. No further data would be collected after withdrawal.

In addition, the Investigator may discontinue a participant from the intervention at any time if the Investigator considers it necessary for any reason including, but not limited to:

- Participant declining surgery
- Pregnancy
- Ineligibility (either arising during the study or retrospectively having been overlooked at screening)
- Significant protocol deviation
- Significant non-adherence with the intervention or trial requirements
- Clinical decision

The follow-up of participants that have withdrawn from the intervention but not from active follow-up will continue with the standard follow-up assessments of the study.

Data from randomised participants who undergo curative surgery for colorectal cancer will be analysed. Post-randomisation withdrawal from the study will not result in exclusion of the data for that participant from the analysis. However, participants will be replaced as per section 9.3 if

- they have been randomised but have not undergone curative surgery for colorectal cancer
- they have been randomised but deemed ineligible during the study or retrospectively or
- they have withdrawn before randomisation.

The type of withdrawal and reason for withdrawal will be recorded in the eCRF.

If the participant is withdrawn due to an AE, the investigator will arrange for frequent telephone calls as agreed with the participant until the AE has resolved or stabilised (also see sections 9.7, 10, and 13.3.2) and up to the point the participant completes the study. If a participant is withdrawn from treatment due to pregnancy the pregnancy will be followed-up to outcome. See the Safety Reporting section below.

The type of withdrawal and reason for withdrawal will be recorded in the eCRF.

9.11. Definition of End of Study

The end of study is the point at which all the study data has been entered and queries resolved.

10. SAFETY REPORTING

Potential AEs will be recorded at pre-operative follow-up assessment 1 and the post-operative assessment. Participants may contact the research team (e.g., over the phone) at any time point during the course of the study to report potential AEs. AEs will be recorded as part of the eCRF of the subsequent assessment (e.g., an AE occurring between pre-operative assessment 1 and 2 will be recorded as part of the pre-operative assessment 2.

One previous trial reported no SAEs due to the TDR, whereas another reported two SAEs potentially related to the TDR in the same participant.[21, 22] There was evidence that for every five people one would experience an adverse, mostly mild, event because of the TDR programme.[21] Constipation, headache, fatigue, and dizziness are the most common AEs albeit occurring in a minority of participants (<8% each) and disappeared over time.[21, 22] Less common side effects include dry mouth, abdominal pain, bad breath, diarrhoea, hair loss, dry skin, mood changes, and feeling cold.

The safety reporting window begins from the first day of the intervention for participants in the intervention group and from the day of randomisation for participants in the control group. It finishes for both groups when the participant completes the study. The limit of investigator follow-up of SAEs will be until the participant completes the study. This requirement will be the same for all SAEs.

Operative and post-operative complications or the responses to questionnaires (e.g., change in anxiety levels) will not be reported as AEs, as they will be extracted as part of the study outcomes.

10.1. Definition of Serious Adverse Events

A serious adverse event is any untoward medical occurrence that:

- results in death
- is life-threatening
- requires inpatient hospitalisation or prolongation of existing hospitalisation
- results in persistent or significant disability/incapacity
- consists of a congenital anomaly or birth defect.

Other ‘important medical events’ may also be considered a serious adverse event when, based upon appropriate medical judgement, the event may jeopardise the participant and may require medical or surgical intervention to prevent one of the outcomes listed above.

NOTE: The term "life-threatening" in the definition of "serious" refers to an event in which the participant was at risk of death at the time of the event; it does not refer to an event which hypothetically might have caused death if it were more severe.

10.2. Reporting Procedures for Serious Adverse Events

A serious adverse event (SAE) occurring to a participant should be reported to the REC that gave a favourable opinion of the study where in the opinion of the Chief Investigator the event was ‘related’ (resulted from administration of any of the research procedures) and ‘unexpected’ in relation to those procedures. Reports of related and unexpected SAEs should be submitted within 15 working days of the Chief Investigator becoming aware of the event, using the HRA report of serious adverse event form (see HRA website). Mortality is expected to be <4% within this setting but the Trial Steering Committee (TSC) may advise reporting to REC if the frequency of deaths is significantly higher than that expected in this population. As per 10, operative and post-operative complications that could meet the definition of SAEs will not be reported as SAEs, as they will be reported as part of the study outcomes, but the TSC will monitor the frequency of complications and may advise reporting at their discretion.

11. STATISTICS AND ANALYSIS

A Statistical Analysis Plan (SAP) is to be produced separately.

11.1. Statistical Analysis Plan (SAP)

The statistical aspects of the study are summarised here with details fully described in a SAP that will be written and finalised before the time that the first participant is recruited. The SAP will be reviewed prior to final database lock, and, if an amendment is deemed necessary, then this will be documented.

11.2. Description of the Statistical Methods

A table will present the baseline demographic and clinical characteristics. Continuous variables will be summarised using means, standard deviations, and 95% confidence intervals. Medians with interquartile ranges will be presented where appropriate. Categorical variables will be summarised using counts and percentages. Exploratory between-group comparisons will be reported where appropriate. Data will be analysed using appropriate statistical software.

Progression criteria (as defined in Table 2) will be summarised descriptively for all participants [and by trial group, trial site, and neoadjuvant treatment (yes/no) as appropriate]. Uncertainty in the progression criteria will be expressed with 95% confidence intervals.

Table 2: Progression criteria

Sufficient levels of		Criterion Decision	**Green** **Progress**	**Amber** **Progress with changes**		**Red** **Stop**
Recruitment	1a	Rate (n of patients per site per month)	≥0.75	0.46-0.74	Progress by adding sites.	≤0.45
	1b	Number of sites open	≥6 sites	3-5		≤2

Sufficient levels of		Criterion<br>Decision	**Green**<br>**Progress**	**Amber**<br>**Progress with changes**		**Red**<br>**Stop**
	1c	Total N participants recruited	72	44-71		≤43
Engagement	2	Proportion of phone calls answered	≥75%	51-74%	Progress if process evaluation can recommend improvements.	≤50%
Adherence	3	Proportion of intervention participants with ≥5% weight loss from baseline to the day of surgery[2]	≥60%	36-59%		≤35%
Retention	4	% at final follow-up	≥85%	66-84%		≤65%
Safety	5	Safety profile	Based on adverse and serious adverse reactions. Adjudicated by the Data Monitoring and Ethics Committee.			

[1] **Adherence:** Non-adherence will be defined as <2% weight loss from baseline to the day of surgery. Participants will also rate in their weekly phone call their adherence to the intervention on a 0-100 scale.

All other outcomes will be summarised descriptively by trial arm. Where appropriate, the effect size and 95% confidence intervals will be estimated with regression models adjusting for treatment group, baseline value (where applicable), and stratification variables. Both absolute and relative effect sizes will be reported. No subgroup analyses are planned.

Complications will be summarised using:

- count/percentage of participants with any complication
- count/percentage of participants with any complication by grade
- count/percentage of participants with the highest grade of complication reported
- count/percentage of participants with any type of complication
- count of total complications.

11.3. Sample Size Determination

With 72 patients (n=36 per arm), the trial will be 90% powered at one-sided 5% level based on the normal approximation approach to detect whether the proportions for the engagement, adherence, and retention criteria in Table 2 are truly above the upper limit of the red zone (>50% engagement, >35% adherence, >65% follow-up) based on an alternative being in the green zone.[70] The collective power for all three criteria is 85% at 5% level, without multiple testing adjustment. Recalculating the sample size on a binomial approach (sensitivity analysis) provided almost identical estimates.[70]

11.4. Analysis populations

All randomised and eligible participants that underwent surgery will be included in the main analysis on an intention-to-treat principle regardless of withdrawal or non-adherence. A per protocol analysis will include the subsample of intervention participants who achieved ≥5% weight loss from baseline to the day of surgery. The adverse event analysis will include the participants in the control group and the participants commencing the intervention in the intervention group.

11.5. Decision points

No interim analysis is planned.

11.6. Stopping rules

The TSC may formally recommend early termination if needed in line with the TSC charter.

11.7. The Level of Statistical Significance

P-values will not be reported given the feasibility nature of the trial.[71] There will be no adjustment for multiple testing.[72] The 95% confidence intervals will be presented but regarded nominal and descriptive.

11.8. Procedure for Accounting for Missing, Unused, and Spurious Data.

Missing data will be imputed using appropriate methodology, as detailed in the SAP.

11.9. Procedures for Reporting any Deviation(s) from the Original Statistical Plan

Deviations from the original SAP will be reported and justified at publication following the CONSORT guidelines.

11.10. Qualitative Analysis

Qualitative data will be transcribed verbatim, de-identified, and analysed in NVivo.

Analysis of QuinteT qualitative interviews: Data will be analysed using thematic and inductive analysis with constant comparison techniques as per Glaser and Strauss.[73] These techniques, adapted from conversation analysis, are standard in the QuinteT process.[59] Analysis will be iterative and alongside data collection, so that the sample size for each interviewee subgroup will be guided by data saturation. Given the necessary speed of analysis and implementation, an experienced second coder will check the coding and this will be refined on discussion.

Analysis of qualitative interviews with intervention participants: Data will be analysed using thematic analysis while data collection is ongoing.[74] This will aim to identify themes at an explicit level using a realist approach. A second coder will re-code a random 10% sub-sample to validate the coding.

11.11. Health Economic analysis

Appropriate health economic evaluation will be conducted and will be detailed in a separate health economic analysis plan. Data on health economic aspects (intervention costs, healthcare resource use, and quality-adjusted life years) will be collected (see 9.6.3) and described to allow for planning of the health economics analysis in a future definitive trial.

Intervention costs will include training for intervention delivery, intervention delivery, administrative time, and meal replacement products.

Healthcare resource use will be quantified base on self-reported questions as per 9.6.3 and hospital records. The hospital records will be used to quantify secondary care resource use, as it is anticipated that all secondary care for each patient will be received at the recruitment hospital. Secondary care

resource use will include complications, fitness to discharge, operative outcomes, and oncological outcomes as per 9.6.3. Healthcare resource use will be valued using unit costs from UK NHS reference costs and the most recent Personal Social Services Research Unit.

Quality-adjusted life years will be estimated based on the collected EQ-5D-5L data using UK utility values.

12. DATA MANAGEMENT

The plan for the data management of the study is outlined below. There is not a separate Data Management document in use for the study.

12.1. Source Data

Source documents are where data are first recorded, and from which participants' eCRF data are obtained. These include, but are not limited to, hospital records, clinical and office charts, laboratory and pharmacy records, diaries, microfiches, radiographs, and correspondence.

eCRF entries will be considered source data if the eCRF is the site of the original recording (e.g., there is no other written or electronic record of data). All documents will be stored safely in confidential conditions. On all study-specific documents, other than the signed consent, the participant will be referred to by the study participant number/code, not by name.

Audio recordings will be collected using an encrypted audio-recorder (or recorded via an alternative secure device/mechanism, including Sponsor/NHS-approved tools).

12.2. Access to Data

Direct access will be granted to authorised representatives from the Sponsor and host institution for monitoring and/or audit of the study to ensure compliance with regulations.

Names and contact details (address and phone number) of participants receiving the intervention will be shared with the company providing the meal replacement products and the delivery company for the sole purpose of selecting and delivering the products to the participants. Participants will also be informed that their audio recordings are transferred to a professional transcription company for transcription purposes. These transfers will be through an appropriately secure communications procedure in line with the University of Oxford's Information Security Handling Rules.

12.3. Data Recording and Record Keeping

This study will be run using the Surgical Intervention Trials Unit standard operating procedures (SOPs) for guidance.

All trial data, except the audio recording files, will be entered on REDCap, which will host the eCRF. The validation process with be in line with the SOPs and include naming variables, eCRF design, data verification and validation (range and logic tests), test data entry, and data export verification. Each site will be provided with a tablet, so that participants can consent and directly answer questionnaires during study visits on REDCap rather than paper-based copies.

Identifiable, personal data will be retained centrally on REDCap (i.e. by the sponsoring organisation). The participants will be identified by a unique trial specific number and/or code in any database for analysis outside REDCap (i.e., they will be excluded from any data exports for data analysis). Research documents with personal information, such as consent forms, will be held securely at the University of Oxford until the end of the study to allow for the long-term follow-up through medical records to occur. They will then be securely deleted.

Following review to ensure participant anonymity is safeguarded and subject to any reasonable and necessary delay, anonymised research data will be securely archived to a repository following publication of the results where they will be stored indefinitely. These data may be used in future research, here or abroad, and may involve commercial organisations.

In line with the departmental Data Transfer Policy, transfer of the audio recordings from the NHS sites to the University and from the University to University-approved transcription companies will be done securely through the University-approved Nexus365 OneDrive or equivalent. The audio recording files will be securely stored in OneDrive. Copies held by the NHS sites will be deleted following transfer to the University. Copies held by the transcription company will be deleted following receipt of the de-identified transcript by the University (i.e., through upload to OneDrive or equivalent). This will follow the departmental Data Transfer Policy. The audio recordings will be linked to the rest of the data using the unique trial specific ID number in the file name. The copy of the audio recordings held at the University will be retained until completion of relevant analysis and they will then be securely deleted. The de-identified transcripts will be retained in line with the rest of the de-identified research data.

Prior to database lock, the database will be reviewed to ensure all queries have been resolved and the dataset is complete.

The Data Management will be compliant with the University of Oxford's policy (https://researchsupport.admin.ox.ac.uk/policy/data).

13. QUALITY ASSURANCE PROCEDURES

The study may be monitored, or audited in accordance with the current approved protocol, GCP, relevant regulations and SOPs.

13.1. Risk assessment

A risk assessment and monitoring plan will be prepared before the study opens and will be reviewed as necessary over the course of the study to reflect significant changes to the protocol or outcomes of monitoring activities.

13.2. Study monitoring

Regular monitoring will be performed according the Surgical Intervention Trials Unit SOPs. Data will be evaluated for compliance with the protocol and accuracy in relation to source documents.

Data will be monitored for quality and completeness by the Surgical Intervention Trials Unit, using established verification, validation and checking processes. Missing data will be chased until they are received, confirmed as not available, or when the trial is at analysis. Reminders will be sent to participants if

questionnaires are not completed within a specified period (section 9), and researchers will also contact participants by telephone to facilitate data completion where appropriate.

The Surgical Intervention Trials Unit/Sponsor reserve the right to intermittently conduct source data verification exercises on a sample of participants, which will be carried out by staff from the Surgical Intervention Trials Unit/Sponsor. Source data verification will involve direct access to patient notes at the participating NHS Sites and the ongoing central collection of copies of consent forms and other relevant investigation reports.

Following written SOPs, the monitors will verify that the clinical study is conducted and data are generated, documented and reported in compliance with the protocol, GCP and the applicable regulatory requirements.

13.3. Study Committees

1.1.1. Trial Management Committee

The trial management committee will comprise of all named investigators, the trial manager, relevant staff from the Surgical Intervention Trials Unit, patient and public involvement representatives, and other key personnel involved in the trial. It will be responsible for the design of the trial and the day-to-day management in line with the committee's Charter. The Committee will initially meet monthly and the frequency of the meetings will be adjusted depending on progress.

1.1.2. Independent Trial Steering Committee

As this is an unblinded trial (with blinded outcome assessment), a separate Data Monitoring and Ethics Committee is not required. The TSC will also assume the role of the Data Monitoring and Ethics Committee. It will comprise of an independent Chair (academic colorectal surgeon), an independent academic, independent statistician, a patient and public representative.

14. PROTOCOL DEVIATIONS

A study related deviation is a departure from the ethically approved study protocol or other study document or process (e.g. consent process or administration of study intervention) or from Good Clinical Practice (GCP) or any applicable regulatory requirements. Any deviations from the protocol will be documented in a protocol deviation form and filed in the study electronic master file.

The Surgical Intervention Trials Unit's SOPs will be followed for identifying non-compliances, escalation to the central team, and assessment of whether a non-compliance/deviation may be a potential Serious Breach.

15. SERIOUS BREACHES

A "serious breach" is a breach of the protocol or of the conditions or principles of Good Clinical Practice which is likely to affect to a significant degree –

(a) the safety or physical or mental integrity of the trial subjects; or

(b) the scientific value of the research.

In the event that a serious breach is suspected the Sponsor must be contacted within 1 working day. In collaboration with the C.I., the serious breach will be reviewed by the Sponsor and, if appropriate, the Sponsor will report it to the approving REC committee and the relevant NHS host organisation within seven calendar days.

16. ETHICAL AND REGULATORY CONSIDERATIONS

16.1. Declaration of Helsinki

The Investigator will ensure that this study is conducted in accordance with the principles of the Declaration of Helsinki.

16.2. Guidelines for Good Clinical Practice

The Investigator will ensure that this study is conducted in accordance with relevant regulations and with Good Clinical Practice.

16.3. Approvals

Following Sponsor approval, the protocol, informed consent form, PIS and any proposed advertising material will be submitted to an appropriate Research Ethics Committee (REC), and HRA (where required) and host institutions for written approval.

The Investigator will submit and, where necessary, obtain approval from the above parties for all substantial amendments to the original approved documents.

16.4. Other Ethical Considerations

<u>Clinical equipoise</u>

There is some promising evidence that weight loss improves outcomes following other types of surgery, but there is a lack of evidence on whether intentional weight loss improves recovery following colorectal cancer surgery. This leads to clinical equipoise of whether this treatment should be offered and justifies the need for randomisation. Additionally, whether the definitive trial is feasible remains unclear justifying the need for this feasibility trial.

<u>Adverse events</u>

AEs during the intervention period have previously shown to be infrequent and disappear over time (see section 10). The dietitian will support the participants during the frequent consultations in managing potential AEs based on current clinical guidance. The research team will handle the AEs when reported in a sensitive manner and in line with section 10.

16.5. Reporting

The CI shall submit once a year throughout the study, or on request, an Annual Progress report to the REC Committee, HRA (where required) host organisation, Sponsor and funder (where required). In addition, an End of Study notification and final report will be submitted to the same parties.

16.6. Transparency in Research

Prior to the recruitment of the first participant, the trial will have been registered on ISRCTN, a publicly accessible database.

The trial information will be kept up to date during the trial, and the CI or their delegate will upload results within 12 months of the end of the trial declaration.

16.7. Participant Confidentiality

The study will comply with the United Kingdom General Data Protection Regulation (UK GDPR) and Data Protection Act 2018, which require data to be de-identified as soon as it is practical to do so. The processing of the personal data of participants will be minimised by making use of a unique participant study number only on all study documents and any electronic database(s), with the exception of the secure and encrypted eCRF, where participant identifiable information (e.g. names, contact details, consent form) will be stored. All documents will be stored securely and only accessible by study staff and authorised personnel. The study staff will safeguard the privacy of participants' personal data.

16.8. Expenses and Benefits

We will offer participants a voucher following the baseline assessment and another voucher of the same value following the post-operative follow-up assessment as compensation for reasonable travel expenses occurred to attend study visits. The value of the voucher will be £15 or £30 and will depend on the length of travel and from the hospital. This approach will allow us to compensate participants more fairly than a voucher of a single value.

17. FINANCE AND INSURANCE

17.1. Funding

This study is funded by the NIHR (Grant Reference Number NIHR204051).

17.2. Insurance

The University has a specialist insurance policy in place which would operate in the event of any participant suffering harm as a result of their involvement in the research (Newline Underwriting Management Ltd, at Lloyd's of London). NHS indemnity operates in respect of the clinical treatment that is provided.

17.3. Contractual arrangements

Appropriate contractual arrangements will be put in place with all third parties.

18. PUBLICATION POLICY

The Investigators will be involved in reviewing drafts of the manuscripts, abstracts, press releases and any other publications arising from the study. Authors will acknowledge that the study was funded by the National Institute for Health and Care Research (NIHR) under its Research for Patient Benefit (RfPB)

Programme (Grant Reference Number NIHR204051). The views expressed will be those of the author(s) and not necessarily those of the NIHR or the Department of Health and Social Care. Authorship will be determined in accordance with the ICMJE guidelines and other contributors will be acknowledged.

19. DEVELOPMENT OF A NEW PRODUCT/ PROCESS OR THE GENERATION OF INTELLECTUAL PROPERTY

Ownership of IP generated by employees of the University vests in the University. The University will ensure appropriate arrangements are in place as regards any new IP arising from the trial.

19. ARCHIVING

This will be in line with section 12.3. Anonymised data will be indefinitely archived in a repository. Identifiable data will be stored within the secure network of the University of Oxford for 5 years and then securely deleted.

20. REFERENCES

- [1] T. Anthony, J. Long, L.S. Hynan, G.A. Sarosi, Jr., F. Nwariaku, J. Huth, C. Jones, B.J. Parker, R. Rege, Surgical complications exert a lasting effect on disease-specific health-related quality of life for patients with colorectal cancer, Surgery 134(2) (2003) 119-25.
- [2] NHS, 2019/20 National Cost Collection Data Publication, 2021. https://www.england.nhs.uk/publication/2019-20-national-cost-collection-data-publication/. (Accessed 29 Sept 2021.
- [3] H. Cakir, C. Heus, T.J. van der Ploeg, A.P. Houdijk, Visceral obesity determined by CT scan and outcomes after colorectal surgery; a systematic review and meta-analysis, Int J Colorectal Dis 30(7) (2015) 875-82.
- [4] F. Shahjehan, A. Merchea, J.J. Cochuyt, Z. Li, D.T. Colibaseanu, P.M. Kasi, Body Mass Index and Long-Term Outcomes in Patients With Colorectal Cancer, Frontiers in oncology 8 (2018) 620.
- [5] D.E. Flynn, D. Mao, S.T. Yerkovich, R. Franz, H. Iswariah, A. Hughes, I.M. Shaw, D.P.L. Tam, M.D. Chandrasegaram, The impact of comorbidities on post-operative complications following colorectal cancer surgery, PLoS One 15(12) (2020) e0243995.
- [6] F.D. McDermott, A. Heeney, M.E. Kelly, R.J. Steele, G.L. Carlson, D.C. Winter, Systematic review of preoperative, intraoperative and postoperative risk factors for colorectal anastomotic leaks, Br J Surg 102(5) (2015) 462-79.
- [7] Y. He, J. Wang, H. Bian, X. Deng, Z. Wang, BMI as a Predictor for Perioperative Outcome of Laparoscopic Colorectal Surgery: a Pooled Analysis of Comparative Studies, Dis Colon Rectum 60(4) (2017) 433-445.
- [8] Y. Xi, P. Xu, Global colorectal cancer burden in 2020 and projections to 2040, Transl Oncol 14(10) (2021) 101174.
- [9] L. Keaver, B. Xu, A. Jaccard, L. Webber, Morbid obesity in the UK: A modelling projection study to 2035, Scand J Public Health 48(4) (2020) 422-427.
- [10] A. Weimann, M. Braga, F. Carli, T. Higashiguchi, M. Hubner, S. Klek, A. Laviano, O. Ljungqvist, D.N. Lobo, R. Martindale, D.L. Waitzberg, S.C. Bischoff, P. Singer, ESPEN guideline: Clinical nutrition in surgery, Clin Nutr 36(3) (2017) 623-650.

- [11] Macmillan, Prehabilitation evidence and insight review, 2017. https://www.macmillan.org.uk/_images/prehabilitation-evidence-and-insight-review_tcm9-335025.pdf. (Accessed 2 November 2021.
- [12] O. Boney, M. Bell, N. Bell, A. Conquest, M. Cumbers, S. Drake, M. Galsworthy, J. Gath, M.P. Grocott, E. Harris, S. Howell, A. Ingold, M.H. Nathanson, T. Pinkney, L. Metcalf, Identifying research priorities in anaesthesia and perioperative care: final report of the joint National Institute of Academic Anaesthesia/James Lind Alliance Research Priority Setting Partnership, BMJ Open 5(12) (2015) e010006.
- [13] JLA, Living With and Beyond Cancer Top 10, 2018. https://www.jla.nihr.ac.uk/priority-setting-partnerships/living-with-and-beyond-cancer/top-10-priorities.htm. (Accessed 27 Sept 2021.
- [14] D.T. Villareal, S. Chode, N. Parimi, D.R. Sinacore, T. Hilton, R. Armamento-Villareal, N. Napoli, C. Qualls, K. Shah, Weight loss, exercise, or both and physical function in obese older adults, N Engl J Med 364(13) (2011) 1218-29.
- [15] C.P. Snowden, J. Prentis, B. Jacques, H. Anderson, D. Manas, D. Jones, M. Trenell, Cardiorespiratory fitness predicts mortality and hospital length of stay after major elective surgery in older people, Annals of surgery 257(6) (2013) 999-1004.
- [16] A. Heldens, B.C. Bongers, A.F. Lenssen, L.P.S. Stassen, W.F. Buhre, N.L.U. van Meeteren, The association between performance parameters of physical fitness and postoperative outcomes in patients undergoing colorectal surgery: An evaluation of care data, Eur J Surg Oncol 43(11) (2017) 2084-2092.
- [17] M. Kotagal, R.G. Symons, I.B. Hirsch, G.E. Umpierrez, E.P. Dellinger, E.T. Farrokhi, D.R. Flum, S.-C. Collaborative, Perioperative hyperglycemia and risk of adverse events among patients with and without diabetes, Annals of surgery 261(1) (2015) 97-103.
- [18] E. Selvin, N.P. Paynter, T.P. Erlinger, The effect of weight loss on C-reactive protein: a systematic review, Arch Intern Med 167(1) (2007) 31-9.
- [19] J.H. Park, D.G. Watt, C.S. Roxburgh, P.G. Horgan, D.C. McMillan, Colorectal Cancer, Systemic Inflammation, and Outcome: Staging the Tumor and Staging the Host, Annals of surgery 263(2) (2016) 326-36.
- [20] Y. Sun, B. Liu, J.K. Smith, M.L.G. Correia, D.L. Jones, Z. Zhu, A. Taiwo, L.L. Morselli, K. Robinson, A.A. Hart, L.G. Snetselaar, W. Bao, Association of Preoperative Body Weight and Weight Loss With Risk of Death After Bariatric Surgery, JAMA Netw Open 3(5) (2020) e204803.
- [21] N.M. Astbury, P. Aveyard, A. Nickless, K. Hood, K. Corfield, R. Lowe, S.A. Jebb, Doctor Referral of Overweight People to Low Energy total diet replacement Treatment (DROPLET): pragmatic randomised controlled trial, BMJ 362 (2018) k3760.
- [22] M.E. Lean, W.S. Leslie, A.C. Barnes, N. Brosnahan, G. Thom, L. McCombie, C. Peters, S. Zhyzhneuskaya, A. Al-Mrabeh, K.G. Hollingsworth, A.M. Rodrigues, L. Rehackova, A.J. Adamson, F.F. Sniehotta, J.C. Mathers, H.M. Ross, Y. McIlvenna, R. Stefanetti, M. Trenell, P. Welsh, S. Kean, I. Ford, A. McConnachie, N. Sattar, R. Taylor, Primary care-led weight management for remission of type 2 diabetes (DiRECT): an open-label, cluster-randomised trial, Lancet 391(10120) (2018) 541-551.
- [23] R.V. Seimon, A.L. Wild-Taylor, S.E. Keating, S. McClintock, C. Harper, A.A. Gibson, N.A. Johnson, H.A. Fernando, T.P. Markovic, J.R. Center, J. Franklin, P.Y. Liu, S.M. Grieve, J. Lagopoulos, I.D. Caterson, N.M. Byrne, A. Sainsbury, Effect of Weight Loss via Severe vs Moderate Energy Restriction on Lean Mass and Body Composition Among Postmenopausal Women With Obesity: The TEMPO Diet Randomized Clinical Trial, JAMA Netw Open 2(10) (2019) e1913733.
- [24] S. Taheri, H. Zaghloul, O. Chagoury, S. Elhadad, S.H. Ahmed, N. El Khatib, R.A. Amona, K. El Nahas, N. Suleiman, A. Alnaama, A. Al-Hamaq, M. Charlson, M.T. Wells, S. Al-Abdulla, A.B. Abou-Samra, Effect of intensive lifestyle intervention on bodyweight and glycaemia in early type 2 diabetes (DIADEM-I): an open-label, parallel-group, randomised controlled trial, Lancet Diabetes Endocrinol 8(6) (2020) 477-489.
- [25] J.D. Ard, K.H. Lewis, A. Rothberg, A. Auriemma, S.L. Coburn, S.S. Cohen, J. Loper, L. Matarese, W.J. Pories, S. Periman, Effectiveness of a Total Meal Replacement Program (OPTIFAST Program) on Weight Loss: Results from the OPTIWIN Study, Obesity (Silver Spring) 27(1) (2019) 22-29.

- [26] D.A. Koutoukidis, S.A. Jebb, F. Mozes, J.W. Tomlinson, M. Pavlides, P. Aveyard, J. Cobbold, Changes in liver fibrosis and inflammation estimated non-invasively after treatment with a low-energy total diet replacement programme in people with non-alcoholic steatohepatitis: a single-arm trial, Preliminary results.
- [27] S.A.L. Price, P. Sumithran, A.J. Nankervis, M. Permezel, L.A. Prendergast, J. Proietto, Impact of preconception weight loss on fasting glucose and pregnancy outcomes in women with obesity: A randomized trial, Obesity (Silver Spring) 29(9) (2021) 1445-1457.
- [28] P. Christensen, M. Henriksen, E.M. Bartels, A.R. Leeds, T. Meinert Larsen, H. Gudbergsen, B.F. Riecke, A. Astrup, B.L. Heitmann, M. Boesen, R. Christensen, H. Bliddal, Long-term weight-loss maintenance in obese patients with knee osteoarthritis: a randomized trial, Am J Clin Nutr 106(3) (2017) 755-763.
- [29] C.J. Haywood, L.A. Prendergast, K. Purcell, L. Le Fevre, W.K. Lim, M. Galea, J. Proietto, Very Low Calorie Diets for Weight Loss in Obese Older Adults-A Randomized Trial, J Gerontol A Biol Sci Med Sci 73(1) (2017) 59-65.
- [30] R. Wijesurendra, M. Spartera, S. Neubauer, S.A. Jebb, D.A. Koutoukidis, P. Aveyard, C. Antoniades, J. Smith, B. Casadei, Weight Loss in Elderly Patients with Atrial Fibrillation (LOSE-AF): A Randomised Controlled Trial, Preliminary results.
- [31] NHS, Low calorie diets to treat obesity and Type 2 diabetes, 2020. https://www.england.nhs.uk/diabetes/treatment-care/low-calorie-diets/. (Accessed 15 October 2021.
- [32] N.M. Astbury, K. Tudor, P. Aveyard, S.A. Jebb, Heterogeneity in the uptake, attendance, and outcomes in a clinical trial of a total diet replacement weight loss programme, BMC Med 18(1) (2020) 86.
- [33] A. Downing, E.J. Morris, M. Richards, J. Corner, P. Wright, D. Sebag-Montefiore, P. Finan, P. Kind, C. Wood, S. Lawton, R. Feltbower, R. Wagland, S. Vernon, J. Thomas, A.W. Glaser, Health-related quality of life after colorectal cancer in England: a patient-reported outcomes study of individuals 12 to 36 months after diagnosis, Journal of clinical oncology : official journal of the American Society of Clinical Oncology 33(6) (2015) 616-24.
- [34] J.S. Abelson, A. Chait, M.J. Shen, M. Charlson, A. Dickerman, H.L. Yeo, Sources of distress among patients undergoing surgery for colorectal cancer: a qualitative study, J Surg Res 226 (2018) 140-149.
- [35] A. Beck, H.V. Thaysen, C.H. Soegaard, J. Blaakaer, L. Seibaek, Investigating the experiences, thoughts, and feelings underlying and influencing prehabilitation among cancer patients: a qualitative perspective on the what, when, where, who, and why, Disability and rehabilitation (2020) 1-8.
- [36] N.M. Astbury, C. Albury, R. Nourse, S.A. Jebb, Participant experiences of a low-energy total diet replacement programme: A descriptive qualitative study, PLoS One 15(9) (2020) e0238645.
- [37] S.M. Bluethmann, K. Basen-Engquist, S.W. Vernon, M. Cox, K.P. Gabriel, S.A. Stansberry, C.L. Carmack, J.A. Blalock, W. Demark-Wahnefried, Grasping the 'teachable moment': time since diagnosis, symptom burden and health behaviors in breast, colorectal and prostate cancer survivors, Psycho-oncology 24(10) (2015) 1250-1257.
- [38] R.J. Beeken, K. Williams, J. Wardle, H. Croker, "What about diet?" A qualitative study of cancer survivors' views on diet and cancer and their sources of information, European journal of cancer care 25(5) (2016) 774-83.
- [39] M. van Zutphen, H.C. Boshuizen, D.E. Kok, H. van Baar, A. Geijsen, E. Wesselink, R.M. Winkels, H.K. van Halteren, J.H.W. de Wilt, E. Kampman, F.J.B. van Duijnhoven, Colorectal cancer survivors only marginally change their overall lifestyle in the first 2 years following diagnosis, Journal of cancer survivorship : research and practice 13(6) (2019) 956-967.
- [40] W. Demark-Wahnefried, L.Q. Rogers, J.T. Gibson, S. Harada, A.D. Fruge, R.A. Oster, W.E. Grizzle, L.A. Norian, E.S. Yang, D. Della Manna, L.W. Jones, M. Azrad, H. Krontiras, Randomized trial of weight loss in primary breast cancer: Impact on body composition, circulating biomarkers and tumor characteristics, International journal of cancer. Journal international du cancer 146(10) (2020) 2784-2796.

- [41] W. Demark-Wahnefried, J.W. Nix, G.R. Hunter, S. Rais-Bahrami, R.A. Desmond, B. Chacko, C.D. Morrow, M. Azrad, A.D. Fruge, Y. Tsuruta, T. Ptacek, S.A. Tully, R. Segal, W.E. Grizzle, Feasibility outcomes of a presurgical randomized controlled trial exploring the impact of caloric restriction and increased physical activity versus a wait-list control on tumor characteristics and circulating biomarkers in men electing prostatectomy for prostate cancer, BMC cancer 16 (2016) 61.
- [42] A.D. Fruge, J.A. Dasher, D. Bryan, S. Rais-Bahrami, W. Demark-Wahnefried, G.R. Hunter, Physiological Effort in Submaximal Fitness Tests Predicts Weight Loss in Overweight and Obese Men with Prostate Cancer in a Weight Loss Trial, Int J Cancer Clin Res 4(2) (2017).
- [43] K. Inoue, S. Yoshiuchi, M. Yoshida, N. Nakamura, S. Nakajima, A. Kitamura, K. Mouri, T. Michiura, H. Mukaide, T. Ozaki, H. Miki, H. Yanagimoto, S. Satoi, M. Kaibori, M. Hamada, Y. Kimura, M. Kon, Preoperative weight loss program involving a 20-day very low-calorie diet for obesity before laparoscopic gastrectomy for gastric cancer, Asian J Endosc Surg 12(1) (2019) 43-50.
- [44] C. Hu, Q. Zhang, X. Jin, L. Zhang, Y. Zhang, Q. Zhu, M. Tang, G. Lyv, H. Shi, A paradox between preoperative overweight/obesity and change in weight during postoperative chemotherapy and its relationship to survival in stage and colorectal cancer patients, Clin Nutr 40(4) (2021) 2410-2419.
- [45] S. Temraz, H. Tamim, A. Mailhac, F. Nassar, N. Moukalled, F. Jamali, A. Taher, Could Preoperative Unintended Weight Loss Predispose to Postoperative Thrombosis in Patients Undergoing Colorectal Cancer Surgery? An Analysis of the NSQIP Data, J Am Coll Nutr 40(2) (2021) 141-147.
- [46] J. Xiao, B.J. Caan, E.M. Cespedes Feliciano, J.A. Meyerhardt, C.H. Kroenke, V.E. Baracos, E. Weltzien, M.L. Kwan, S.E. Alexeeff, A.L. Castillo, C.M. Prado, The association of medical and demographic characteristics with sarcopenia and low muscle radiodensity in patients with nonmetastatic colorectal cancer, Am J Clin Nutr 109(3) (2019) 615-625.
- [47] E. Cava, N.C. Yeat, B. Mittendorfer, Preserving Healthy Muscle during Weight Loss, Adv Nutr 8(3) (2017) 511-519.
- [48] A. Bosy-Westphal, E. Kossel, K. Goele, W. Later, B. Hitze, U. Settler, M. Heller, C.C. Gluer, S.B. Heymsfield, M.J. Muller, Contribution of individual organ mass loss to weight loss-associated decline in resting energy expenditure, Am J Clin Nutr 90(4) (2009) 993-1001.
- [49] K.M. Beavers, M.E. Miller, W.J. Rejeski, B.J. Nicklas, S.B. Kritchevsky, Fat mass loss predicts gain in physical function with intentional weight loss in older adults, J Gerontol A Biol Sci Med Sci 68(1) (2013) 80-6.
- [50] H.C. Felix, D.S. West, Effectiveness of weight loss interventions for obese older adults, American journal of health promotion : AJHP 27(3) (2013) 191-9.
- [51] D.W. Kitzman, P. Brubaker, T. Morgan, M. Haykowsky, G. Hundley, W.E. Kraus, J. Eggebeen, B.J. Nicklas, Effect of Caloric Restriction or Aerobic Exercise Training on Peak Oxygen Consumption and Quality of Life in Obese Older Patients With Heart Failure With Preserved Ejection Fraction: A Randomized Clinical Trial, JAMA 315(1) (2016) 36-46.
- [52] P. Christensen, R. Frederiksen, H. Bliddal, B.F. Riecke, E.M. Bartels, M. Henriksen, S.R.T. Juul, H. Gudbergsen, K. Winther, A. Astrup, R. Christensen, Comparison of three weight maintenance programs on cardiovascular risk, bone and vitamins in sedentary older adults, Obesity (Silver Spring) 21(10) (2013) 1982-90.
- [53] P.G. Vaughan-Shaw, L. Zgaga, L.Y. Ooi, E. Theodoratou, M. Timofeeva, V. Svinti, M. Walker, F. O'Sullivan, A. Ewing, S. Johnston, F.V.N. Din, H. Campbell, S.M. Farrington, M.G. Dunlop, Low plasma vitamin D is associated with adverse colorectal cancer survival after surgical resection, independent of systemic inflammatory response, Gut 69(1) (2020) 103-111.
- [54] M.A. Guinter, M.L. McCullough, S.M. Gapstur, P.T. Campbell, Associations of Pre- and Postdiagnosis Diet Quality With Risk of Mortality Among Men and Women With Colorectal Cancer, Journal of clinical oncology : official journal of the American Society of Clinical Oncology (2018) JCO1800714.
- [55] S. Kent, P. Aveyard, N. Astbury, B. Mihaylova, S.A. Jebb, Is Doctor Referral to a Low-Energy Total Diet Replacement Program Cost-Effective for the Routine Treatment of Obesity?, Obesity (Silver Spring) 27(3) (2019) 391-398.

- [56] Y. Xin, A. Davies, L. McCombie, A. Briggs, C.M. Messow, E. Grieve, W.S. Leslie, R. Taylor, M.E.J. Lean, Within-trial cost and 1-year cost-effectiveness of the DiRECT/Counterweight-Plus weight-management programme to achieve remission of type 2 diabetes, Lancet Diabetes Endocrinol 7(3) (2019) 169-172.
- [57] G. Maston, J. Franklin, A.A. Gibson, E. Manson, S. Hocking, A. Sainsbury, T.P. Markovic, Attitudes and Approaches to Use of Meal Replacement Products among Healthcare Professionals in Management of Excess Weight, Behav Sci (Basel) 10(9) (2020).
- [58] A.S. Anderson, S. Caswell, M. Wells, R.J. Steele, Obesity and lifestyle advice in colorectal cancer survivors - how well are clinicians prepared?, Colorectal Dis 15(8) (2013) 949-57.
- [59] J.L. Donovan, L. Rooshenas, M. Jepson, D. Elliott, J. Wade, K. Avery, N. Mills, C. Wilson, S. Paramasivan, J.M. Blazeby, Optimising recruitment and informed consent in randomised controlled trials: the development and implementation of the Quintet Recruitment Intervention (QRI), Trials 17(1) (2016) 283.
- [60] L. Rooshenas, L.J. Scott, J.M. Blazeby, C.A. Rogers, K.M. Tilling, S. Husbands, C. Conefrey, N. Mills, R.C. Stein, C. Metcalfe, A.J. Carr, D.J. Beard, T. Davis, S. Paramasivan, M. Jepson, K. Avery, D. Elliott, C. Wilson, J.L. Donovan, g. By-Band-Sleeve study, C.s. group, H.-s. group, g. Optima prelim study, g. Romio feasibility study, The QuinteT Recruitment Intervention supported five randomized trials to recruit to target: a mixed-methods evaluation, Journal of clinical epidemiology 106 (2019) 108-120.
- [61] N.a.A.N. EFSA Panel on Dietetic Products, Scientific Opinion on the essential composition of total diet replacements for weight control, EFSA Journal 13 (2015) 3957.
- [62] C. Harper, J. Maher, A. Grunseit, R.V. Seimon, A. Sainsbury, Experiences of using very low energy diets for weight loss by people with overweight or obesity: a review of qualitative research, Obesity reviews : an official journal of the International Association for the Study of Obesity 19(10) (2018) 1412-1423.
- [63] NHS, National Cancer Waiting Times Monitoring Dataset v12, 2022. https://www.england.nhs.uk/wp-content/uploads/2022/03/B1368-national-cancer-waiting-times-monitoring-dataset-guidance-version-12..0-march-2022-2.pdf. (Accessed 27 October 2022.
- [64] P.-A.T. Collaborative, SupPoRtive Exercise Programmes for Accelerating REcovery after major ABdominal Cancer surgery trial (PREPARE-ABC): Pilot phase of a multicentre randomised controlled trial, Colorectal Dis (2021).
- [65] PSSRU, Client Service Receipt Inventory (CSRI), 2022. https://www.pssru.ac.uk/csri/client-service-receipt-inventory/. (Accessed 14 August 2022.
- [66] P.A. Clavien, J. Barkun, M.L. de Oliveira, J.N. Vauthey, D. Dindo, R.D. Schulick, E. de Santibanes, J. Pekolj, K. Slankamenac, C. Bassi, R. Graf, R. Vonlanthen, R. Padbury, J.L. Cameron, M. Makuuchi, The Clavien-Dindo classification of surgical complications: five-year experience, Annals of surgery 250(2) (2009) 187-96.
- [67] M. Sekhon, M. Cartwright, J.J. Francis, Development of a theory-informed questionnaire to assess the acceptability of healthcare interventions, BMC health services research 22(1) (2022) 279.
- [68] G. Guest, A. Bunce, L. Johnson, How Many Interviews Are Enough?:An Experiment with Data Saturation and Variability, Field Methods 18(1) (2006) 59-82.
- [69] C. Wilson, L. Rooshenas, S. Paramasivan, D. Elliott, M. Jepson, S. Strong, A. Birtle, D.J. Beard, A. Halliday, F.C. Hamdy, R. Lewis, C. Metcalfe, C.A. Rogers, R.C. Stein, J.M. Blazeby, J.L. Donovan, Development of a framework to improve the process of recruitment to randomised controlled trials (RCTs): the SEAR (Screened, Eligible, Approached, Randomised) framework, Trials 19(1) (2018) 50.
- [70] M. Lewis, K. Bromley, C.J. Sutton, G. McCray, H.L. Myers, G.A. Lancaster, Determining sample size for progression criteria for pragmatic pilot RCTs: the hypothesis test strikes back!, Pilot Feasibility Stud 7(1) (2021) 40.
- [71] S.M. Eldridge, C.L. Chan, M.J. Campbell, C.M. Bond, S. Hopewell, L. Thabane, G.A. Lancaster, P.c. group, CONSORT 2010 statement: extension to randomised pilot and feasibility trials, Pilot Feasibility Stud 2 (2016) 64.

- [72] G. Li, M. Taljaard, E.R. Van den Heuvel, M.A. Levine, D.J. Cook, G.A. Wells, P.J. Devereaux, L. Thabane, An introduction to multiplicity issues in clinical trials: the what, why, when and how, Int J Epidemiol 46(2) (2017) 746-755.
- [73] B. Glaser, A. Strauss, The Discovery of Grounded Theory, Aldine Publishing Company, Hawthorne, New York, 1967.
- [74] V. Braun, V. Clarke, Using thematic analysis in psychology, Qualit Res Psychol 3 (2006) 77-101.

21. APPENDIX A: STUDY FLOW CHART

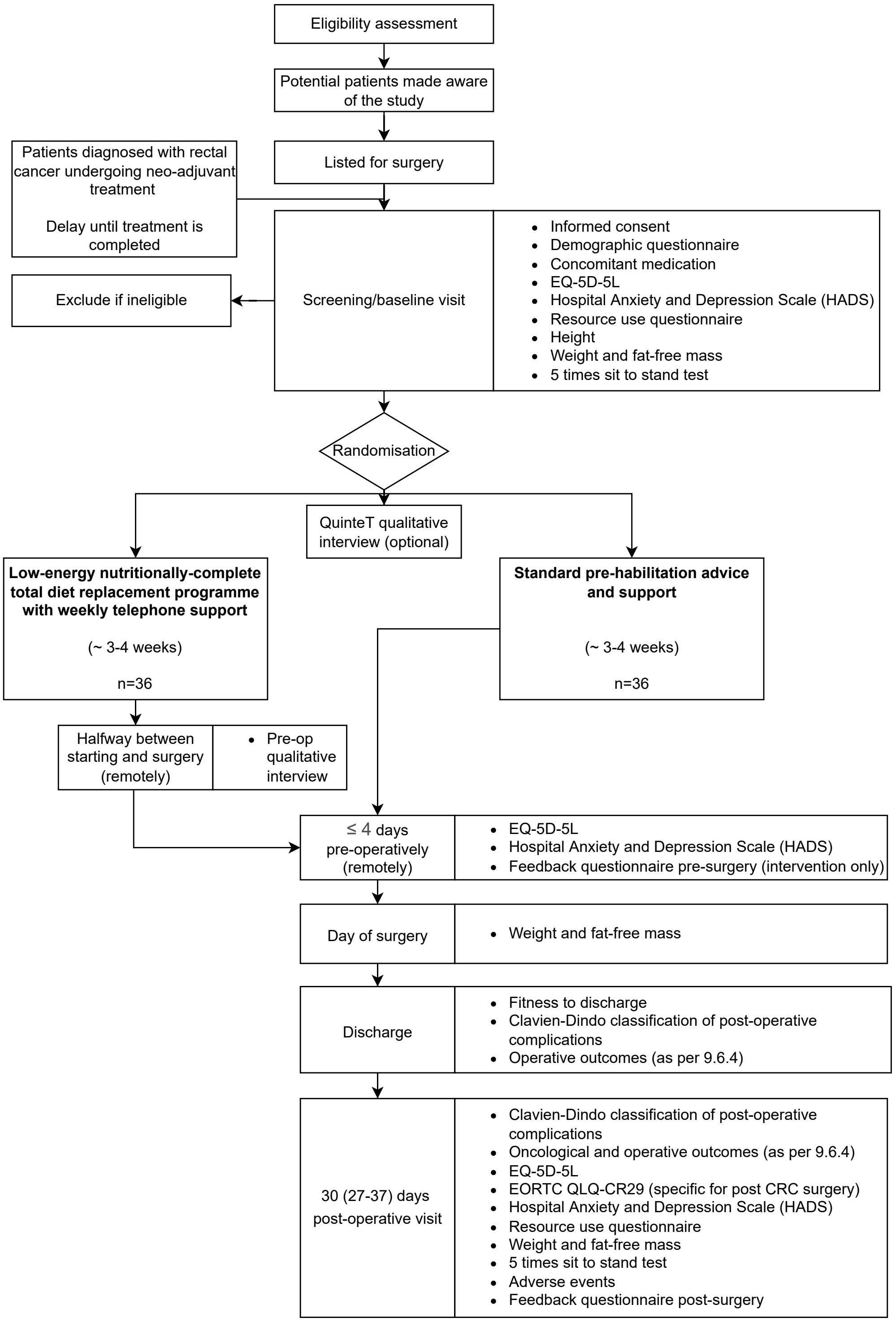

Throughout: qualitative interviews with clinical and research staff

22. APPENDIX B: SCHEDULE OF STUDY PROCEDURES

	**Assessments**								
	From suspicion of cancer to diagnosis	From diagnosis to 3 d post diagnosis	0-4 days post randomisation	Halfway from starting intervention to surgery (± 3 d)	4 d pre-op to day of surgery	Admission	Discharge (~5-7 days post-op)	30 d post-op (27-37 d)	3y (± 6m) post-op
**Procedures**	**Pre-screening**	**Screening / baseline**	**Pre-op 1**	**Pre-op 2**	**Pre-op 3**	**Pre-op 4**	**Discharge**	**Post-op**	**Long-term follow-up**
Informed consent		X							
Eligibility assessment	X	X							
Demographic q		X							
Concomitant medication		X							
Randomisation		X							
QuinteT qualitative interview			X - optional						
EQ-5D-5L q		X			X			X	
EORTC-QLQ-CR29 q								X	
HADS q		X			X			X	
Resource use q		X						X	
Pre-op qualitative interview*				X					
Feedback q pre-surgery*					X				
Feedback q post-surgery								X	
Height		X							
Weight & fat-free mass		X				X		X	
5 times sit to stand test		X						X	
Fitness of discharge assessment							X		
Complications (Clavien-Dindo)							X	X	
Operative outcomes							X	X	X
Oncological outcomes								X	X
Fidelity of intervention delivery				←	→				
Record AEs, as applicable				←	—	—	—	→ X	
Qualitative interviews with staff	Throughout the trial								
* Intervention group only. y: years, m: months, d: days, pre-op: pre-operatively, post-op: post-operatively, q: questionnaire, AEs: adverse events									

23. APPENDIX C: QUINTET CONSENT PROCESS

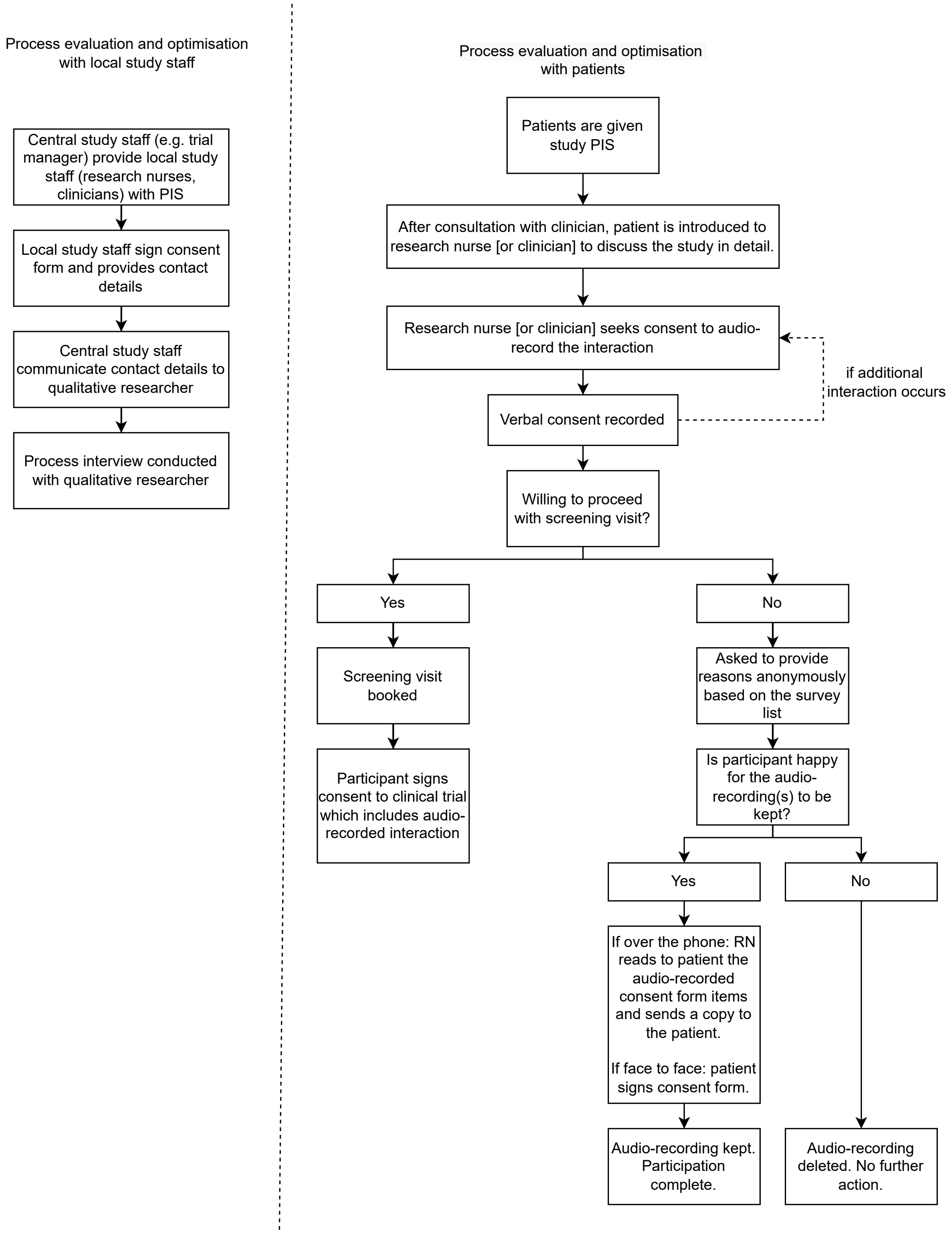

24. APPENDIX D: GUIDE FOR THE QUINTET QUALITATIVE INTERVIEW WITH PARTICIPANTS POST-RANDOMISATION

Topic	Question	Prompt
Introduction	Thank you for taking the time for the interview. The aim of the interview is to tell me your thoughts about how you found the study processes so far. We welcome all comments, positive and negative and indeed sometimes the negatives comments are the most helpful, so please feel free to express your views.	
	When did you first hear about this study?<br>- From whom?<br>- What were your initial thoughts?	- Cancer nurse specialist<br>- Surgeon<br>- Researcher/research nurse
	What do you understand about the study?	What is it trying to find out?
	Tell me about how you made the decision to join the study.	At what point did you decide? What/who helped you to decide?<br>- Improve recovery<br>- Prepare for surgery<br>- Clinician endorsement<br>- Feel in control<br>- Teachable moment
	What has helped you sign up?	
	Any aspects of the study that you were uncertain or concerned about?	
	How did you feel about finding out that the choice of having or not having the diet was done at random? Did it make sense to you why it had to be done that way?	
	How did you feel when you find out that you were randomised to the [weight loss/control] group?	
	In your opinion, what are the possible benefits and risks of the diet?	- Interfering with daily life<br>- Confidence following it<br>- Acceptability
	How have you found the study visit?	
Close down	Any further comments? Thank you for taking part in the interview.	

25. APPENDIX E: GUIDE FOR QUALITATIVE INTERVIEW WITH INTERVENTION PARTICIPANTS

Topic	Question	Prompt
Introduction	Thank you for taking the time for the interview. The aim of the interview is to tell me your thoughts about the diet. We welcome all comments, positive and negative and indeed sometimes the negatives comments are the most helpful, so please feel free to express your views.	
Overall view	How are you finding the diet?	
Delivery	How do you find your contact with the dietitian?	- Over the phone contact<br>- Length of calls<br>- Knowledge<br>- Advice and support
Engagement: barriers and facilitators	What has made it difficult to attend the phone calls?	- Work<br>- Family<br>- Other commitments<br>- Feeling unwell
	What has helped you to attend the phone calls?	- Improve recovery<br>- Support by dietitian
	How do you keep track of your diet?	- Log of shakes<br>- Log of weight<br>- Log of blood pressure/glucose
Adherence: barriers and facilitators	How much effort do you feel you need to put in to follow the recommended diet?	
	If someone was considering starting the diet, what would you say it's like to follow the diet?	What has made it easy to stick to the diet?<br>- Motivation to improve recovery<br>- Support by dietitian<br>- Weight loss<br>- Ease of use of shakes<br>- Being part of a trial
	If someone was considering starting the diet, what would you say they need to be aware of?	Any difficulties sticking to the diet?<br>- Temptations<br>- Flavour<br>- Social occasions<br>- Side effects
	How has your family and friends found it while you are on the diet?	- Approval/disapproval<br>- Supportive and reinforcing / not supportive

Topic	Question	Prompt
	How have you been feeling during the diet?	- Positive/negative comments/actions<br>- Unhappy, happy<br>- Different compared with before starting
	How confident are you that you can continue to follow the diet?<br>How confident are you that the diet will benefit you?	
Symptoms of both TDR and cancer	Have you had any constipation or diarrhoea? If yes, has this [constipation/diarrhoea] worried you?	
Physical activity	Have you changed your physical activity since diagnosis?	- Type<br>- Frequency<br>- Intensity<br>- Length
Future research questions	We want to see how we can improve this diet in the future and we would like to ask you your honest opinion on the following.<br>Losing more weight might improve recovery after surgery to a greater extent. However, that means delaying the surgery for a couple of weeks to lose this weight. If your surgery were to delayed by up to a couple of weeks to lose another 4 pounds/2kilos, would you have been as willing to join the trial?	
	We would like to test how this diet works together with physical activity. Now that you know how it feels to be on this diet, how would you feel if in addition to the diet, we had asked you to increase your physical activity?	If positive<br>- type of activity e.g., walking, resistance<br><br>If negative<br>- why not, hunger, asking “too much”, too tired
	How would you feel about re-starting the diet a couple of months after you recover from surgery to help you lose more weight and improve your long-term health?	
Close down	Any further comments? Thank you for taking part in the interview.	

26. APPENDIX F: GUIDE FOR QUALITATIVE INTERVIEW WITH STAFF

Topic	Question	Prompt
Warm-up	Thank you for taking the time for the interview. Let's start by telling me your thoughts about the trial overall.	
Need for the trial	Do you feel there is clinical equipoise?<br><br>What are your thoughts on the design of the trial?	Expand on yes/no answer
Attitudes	What do you think of the diet being tested?<br>What would be your concerns about the trial and the diet?<br>How do you think this intervention fits within the local pre-habilitation services?	
Recruitment	What are the challenges discussing this trial with patients?<br><br>What, if anything, has made you not discuss the trial with a patient meeting the inclusion/exclusion criteria?<br>How do you think patients reacted to the idea for the trial?<br>What has made the recruitment more difficult than expected?<br>What would make the recruitment easier?	
Trial processes	Based on your experience of the trial, what would improve the trial processes?	- Questionnaire completion<br>- Medical records extraction<br>- Blinding<br>- Communication with central team
Applicability and future	How relevant the results will be to your own practice?<br>How could it be embedded in usual care?<br>What do you think the next steps should be?	 <br>- Diet paired with physical activity<br>- Extension of diet pre-surgery<br>-Extension of diet post-surgery
Co-ordination	[For research nurses and dietitians only]: How have you found the communication with the [research nurse/dietitian]? What can be done to improve this?	
Close down	Any additional comments? Thank you for taking the time for this interview.	

27. APPENDIX G: AMENDMENT HISTORY

Amendment No.	Protocol Version No.	Date issued	Author(s) of changes	Details of Changes made
1.0	2.0	05/01/2023	Dimitrios Koutoukidis	1. Update of section 11.11 to allow for health economic evaluation
2.0	3.0	27/02/2023	Dimitrios Koutoukidis	2. Removal of bars and mousses as the new supplier of meal replacement products does not provide them. However, the new products adhere the same legislation and are interchangeable nutritionally to the original supplier.<br>3. Correction of a typo.
3.0	4.0	12/09/2023	Dimitrios Koutoukidis	1. Revisions to exclusion criteria on dietary requirements, insulin use, and risk of intestinal obstruction.<br>2. Update of administrative details.
4.0	5.0	07/06/2024	Sophie Reynolds	1. Update of study synopsis with extension to planned recruitment period.
5.0	6.0	19/06/2024	Sophie Reynolds	1. Correction of typo to protocol version/date number.

				2. Formatting numbering correction.

List details of all protocol amendments here whenever a new version of the protocol is produced.

Protocol amendments must be submitted to the Sponsor for approval prior to submission to the REC committee and HRA (where required).

CARE

Pre-operative intentional weight loss to support post-operative recovery in patients with overweight and colorectal cancer: the CARE feasibility randomised controlled trial

(Short title: Could supported weight loss reduce bowel cancer surgery complications?)

Statistical Analysis Plan

Version v2.0 07Oct2024

Linked to SAP - Data Definitions and Tables
Version v1.0_30Sep2023

Based on Protocol version v6.0 19Jun2024

Trial registration: IRAS Project ID: 320173

Surgical Intervention Trials Unit (SITU)

CONTENTS

1. INTRODUCTION 3
1.1 KEY PERSONNEL 3
1.2 CHANGES FROM PREVIOUS VERSION OF SAP 4
2. BACKGROUND AND OBJECTIVES 5
2.1 BRIEF SYNOPSIS 5
3. STUDY METHODS 5
3.1 TRIAL DESIGN/FRAMEWORK 5
3.2 RANDOMISATION AND BLINDING 5
3.3 SAMPLE SIZE, HYPOTHESIS TESTING, AND PROGRESSION DECISION 6
3.4 STATISTICAL INTERIM ANALYSIS, DATA REVIEW AND STOPPING GUIDELINES 6
3.4.1 Interim Analysis 6
3.4.2 Stopping rules 6
3.4.3 Trial Steering, Data Monitoring, and Ethics Committee (TSDMEC) 6
3.5 TIMING OF FINAL ANALYSIS 7
3.6 BLINDED ANALYSIS 7
3.7 STATISTICAL ANALYSIS OUTLINE 7
3.7.1 Primary outcomes (referred to as “trial progression criteria”) 7
3.7.2 Secondary and other outcomes 8
4. STATISTICAL PRINCIPLES 9
4.1 STATISTICAL SIGNIFICANCE AND MULTIPLE TESTING 9
4.2 DEFINITION OF ANALYSIS POPULATIONS 9
5. TRIAL POPULATION AND DESCRIPTIVE ANALYSES 9
5.1 REPRESENTATIVENESS OF STUDY SAMPLE AND PATIENT THROUGHPUT 9
5.2 WITHDRAWAL FROM TREATMENT AND/OR FOLLOW-UP 9
5.3 BASELINE CHARACTERISTICS 10
5.4 UNBLINDING 10
5.5 TREATMENT ADHERENCE WITH DETAILS OF INTERVENTION 10
5.6 RELIABILITY 10
6. ANALYSIS 11
6.1 OUTCOME DEFINITIONS 11
6.2 ANALYSIS METHODS 11
6.3 MISSING DATA 11
6.4 SENSITIVITY ANALYSIS 12
6.5 PRE-SPECIFIED SUBGROUP ANALYSIS 12
6.6 SUPPLEMENTARY/ADDITIONAL ANALYSES AND OUTCOMES 12
6.7 HARMS 12
6.8 HEALTH ECONOMICS AND COST EFFECTIVENESS (WHERE APPLICABLE) 12
7. VALIDATION OF THE PRIMARY ANALYSIS 12
8. SPECIFICATION OF STATISTICAL PACKAGES 13
9. PUBLICATION 13
10. REFERENCES 13
APPENDIX: GLOSSARY OF ABBREVIATIONS 14
APPENDIX: STUDY FLOWCHART 15

1. INTRODUCTION

This document details the proposed data presentation and analysis for the main paper(s) and final study reports from the National Institute for Health Research-funded study named "Pre-operative intentional weight loss to support post-operative recovery in patients with overweight and colorectal cancer: the CARE feasibility randomised controlled trial". The results reported in these papers should follow the strategy set out here. Subsequent analyses of a more exploratory nature will not be bound by this strategy, though they are expected to follow the broad principles laid down here. The principles are not intended to curtail exploratory analysis (for example, to decide cut-points for categorisation of continuous variables), nor to prohibit accepted practices (for example, data transformation prior to analysis), but they are intended to establish the rules that will be followed, as closely as possible, when analysing and reporting the trial. This document follows published guidelines regarding the content of statistical analysis plans for clinical trial (Gamble et al).

The analysis strategy will be available on request when the principal papers are submitted for publication in a journal. Suggestions for subsequent analyses by journal editors or referees, will be considered carefully, and carried out as far as possible in line with the principles of this analysis strategy. If reported, the analyses will be marked as post-hoc and the source of the suggestion will be acknowledged.

Any deviations from the statistical analysis plan will be described and justified in the final report of the trial. The analysis should be carried out by an identified, appropriately qualified and experienced statistician or researcher, who should ensure the integrity of the data during their processing. Examples of such procedures include quality control and evaluation procedures.

Integral to this Statistical Analysis Plan (SAP) is the SAP – Data Definitions and Tables document which will include full detailed descriptions of all key outcomes, including their definition, generation and how they will be reported at the end of the study. These two documents should be read in tandem.

1.1 Key personnel

Author(s)

- Martyn Hill (Trial Statistician since inception): martyn.hill@nds.ox.ac.uk

Reviewers

- Dr Dimitrios Koutoukidis (CI): dimitrios.koutoukidis@phc.ox.ac.uk
- Dr Richard Parker (TSC independent statistician): richard.parker@ed.ac.uk
- Others as required

Approver (Chief Investigator and others)

- Chief Investigator: Dr Dimitrios Koutoukidis
- Others as required, including Martyn Hill (Trial statistician).

1.2 Changes from previous version of SAP

This is a summary of key changes from earlier versions of SAP, with particular relevance to protocol changes that have an impact on the design, definition, sample size, data quality/collection and analysis of the outcomes will be provided. The protocol version number and date are included.

Version number Issue date	Author of this issue	Protocol Version & Issue date	Significant changes from previous version together with reasons
V0.1_08Dec2022	Martyn Hill	CARE protocol v1.0 181122	Not applicable as this is the 1st issue
v0.02_06Feb2023	Dimitrios Koutoukidis	CARE protocol v1.0 181122	First draft
v0.03_21Feb2023	MH & DK	CARE protocol v1.0 181122	Second draft
V1.0_14Mar2023	MH & DK	CARE protocol v3.0 270223	Clean version 1.0
V2.0_07Oct2024	MH & DK	CARE protocol v6.0 190724	Clean version 2.0. Changes since 1.0 are:<br>• Recruitment period end changed from June 2024 to November 2024<br>• "Definition of Analysis Populations" expanded to include patients who had their surgery less than 20 days since randomisation
			Add to or delete as required

2. BACKGROUND AND OBJECTIVES

2.1 Brief synopsis

This trial is a study of a pre-operative intentional weight loss intervention intended to support post-operative recovery in patients with overweight and colorectal cancer, aka "the CARE feasibility parallel randomised controlled trial". It is intended to examine the feasibility of progression to a definitive randomised controlled trial that will examine whether a supported weight loss intervention aimed at overweight adults awaiting colorectal cancer surgery can reduce complications.

3. STUDY METHODS

3.1 Trial Design/framework

See also the protocol section "Study Design", "Synopsis", "Description of the Statistical Methods"

This is a prospective randomised controlled trial (RCT) with two arms, one for the intervention, one for the control. The timing is as follows

- Total trial length: 5.5 years
- Recruitment period: approx. 21 months (planned for March 2023 to June 2024, later November 2024)
- Individual participant's involvement: approx. 2-3 months
- Long-term follow-up via medical records: up to 3 years

The trial will have an embedded evaluation and optimisation of the recruitment process (QuinteT). Participants will be recruited from hospitals across England.

Participants are expected to be involved in the study for approximately 2-3 months. They will be asked to attend hospital visits for screening, pre-operatively (on the day of surgery), and 30 days post-operatively. They will also remotely complete questionnaires at 1-3 days pre-operatively and have a semi-structured qualitative interview over the phone. The intervention will be delivered over the pre-operative period.

The study flow-chart can be seen in the appendices. At the end of follow-up the outcomes will be summarised and presented.

3.2 Randomisation and Blinding

See also the protocol section "Randomisation" and "Blinding and code-breaking"

Participants will be randomised by the local research team intended to conduct randomisation through a minimisation module on REDCap.

There are two arms, one for the intervention, one for the control.

Eligible participants will be individually randomised with a 1:1 allocation ratio to receive either the intervention or the control through minimisation with a 20% random element. The two stratified variables will be performance status ("0" vs "1-2") and age at diagnosis ("< 70 years" and "≥70 years") with the threshold of 70 chosen as the median age of diagnosis of colorectal cancer.

Allocation concealment is achieved as randomisation occurs after the baseline visit, the randomisation algorithm is unmodifiable and concealed from investigators and the local research teams, and the local research teams have no access to the total number of participants randomised to each group.

It is impossible to blind the participants and research nurses due to the nature of the intervention. Therefore, procedures for breaking the allocation code are not applicable. However, the assessors of the future primary outcome (research nurses conducting the post-operative follow-up visit) will be blinded. The central study team (chief investigator and members of the trial management committee) will be blinded to the adverse events and all post-operative data by trial group. The trial statistician is not blinded.

Full details of the randomisation are available in the latest RBP (Randomisation and Blinding Plan), which is stored in the confidential statistical section of the TMF.

3.3 Sample Size, hypothesis testing, and progression decision

See also the protocol section "Sample Size Determination"

With 72 patients (n=36 per arm), the trial will be 90% powered at one-sided 5% level based on the normal approximation approach to detect whether the proportions for the engagement, adherence, and retention criteria in the table 1 are truly above the upper limit of the red zone (>50% engagement, >35% adherence, >65% follow-up) based on an alternative being in the green zone. The collective power for the three criteria of engagement, adherence, and retention is 85% (93%*92%*99%)[4] at 5% level to detect "GO" signals, without multiple testing adjustment. Recalculating the sample size on a binomial approach (sensitivity analysis) provided almost identical estimates.[1]

We are testing against the upper limit of the red zone when the lower limit of the green zone is hypothesised to be true, therefore:
- Null hypothesis: True feasibility outcome is not greater than the upper limit of the red zone.
- Alternative hypothesis: True feasibility outcome is greater than the upper limit of the red zone.

3.4 Statistical Interim Analysis, Data Review and Stopping guidelines

See also the protocol sections "Decision Points", "Stopping Rules" and "Study Committees"

3.4.1 <u>Interim Analysis</u>
No interim analysis is planned.

3.4.2 <u>Stopping rules</u>
The TSC may formally recommend early termination if needed in line with the TSC charter.

3.4.3 <u>Trial Steering, Data Monitoring, and Ethics Committee (TSDMEC)</u>
As this is an unblinded trial (with blinded outcome assessment), a separate Data Monitoring and Ethics Committee (DMEC) is not required. The independent Trial Steering Committee (TSC) will instead assume the role of the Data Monitoring and Ethics Committee. The resultant TSDMEC will comprise of an independent Chair (academic colorectal surgeon), two independent academics, independent statistician, and a patient and public representative.

3.5 Timing of Final Analysis

See also the protocol section "Decision Points"

The final analysis will occur at the end of the trial and no interim analysis is planned.

3.6 Blinded analysis

See also the protocol section "Blinding And Code-Breaking"

The nature of the study precludes the majority of blinding, however the central study team (chief investigator and members of the trial management committee) will be blinded to the adverse events and all post-operative data by trial group. The study statistician, who will not be blinded to these data, will prepare relevant reports of these data that will be reviewed in the closed sessions of the Trial Steering, Data Monitoring, and Ethics Committee.

3.7 Statistical Analysis Outline

The statistical analysis of the study's three primary outcomes and other outcomes is detailed in the protocol section "Statistics And Analysis", a summary of which is given below:

The study is a feasibility study intended to derive data, not to conduct formal statistical tests on them. Consequently, the outcomes will be summarised and described, not subjected to tests.

Table(s) will present the baseline demographic and clinical characteristics. Continuous variables will be summarised using means, standard deviations, and 95% confidence intervals. Medians with interquartile ranges will be presented where appropriate. Categorical variables will be summarised using counts and percentages. Exploratory between-group comparisons will be reported where appropriate. Any specific statistical analysis of the data will be carried out using appropriate statistical software. Any other analysis done by the Trial Statistician will be done by that software deemed sufficient by the Trial Statistician.

The data fall into two broad categories: the *primary outcomes* (referred to as "trial progression criteria") and the *secondary and other outcomes*. They are treated as follows:

3.7.1 Primary outcomes (referred to as "trial progression criteria")

The trial progression criteria will be summarised descriptively for all participants [and by trial group, trial site, and neoadjuvant treatment (yes/no) as appropriate]. Uncertainty in the progression criteria will be expressed with 95% confidence intervals as well with two-sided 90% confidence intervals (given the one-sided 5% level in the sample size calculation). This uncertainty will be descriptive and will not be considered in the decision to progress to the definitive trial.

Table 1: Primary variables (referred to as "trial progression criteria")

**Sufficient levels of**		**Criterion**<br>**Decision**	**Green**<br>**Progress**	**Amber**<br>**Progress with changes**		**Red**<br>**Stop**
Recruitment	1a	Rate (n of patients per site per month)	≥0.75	0.46-0.74	Progress by adding sites.	≤0.45

Sufficient levels of		Criterion Decision	Green **Progress**	Amber **Progress with changes**		Red **Stop**
	1b	Number of sites open	≥6 sites	3-5		≤2
	1c	Total N participants recruited	72	44-71		≤43
Engagement	2	Proportion of phone calls answered	≥75%	51-74%	Progress if process evaluation can recommend improvements.	≤50%
Adherence	3	Proportion of intervention participants with ≥5% weight loss from baseline to the day of surgery[1]	≥60%	36-59%		≤35%
Retention	4	% at final follow-up	≥85%	66-84%		≤65%
Safety	5	Safety profile	Based on related adverse and related expected and related unexpected serious adverse events. Adjudicated by the Data Monitoring and Ethics Committee.			

[1] **Adherence:** Non-adherence will be defined as <2% weight loss from baseline to the day of surgery. Participants will also rate in their weekly phone call their adherence to the intervention on a 0-100 scale.

In accordance with the multi-criteria aim, the decision to proceed would take into account
- Signal on criterion 1 (recruitment)
- Signal on criterion 5 (safety)

And if 1 and 5 are satisfactory (i.e., at least “amber”), the decision to progress will be based on the worst signal:
➢ If signal = RED for criteria 2 or 3 or 4 -> overall signal is RED
➢ Else, if no signal is RED but signal = AMBER for criteria 2 or 3 or 4 -> overall signal is AMBER
➢ Else, if signal = GREEN for criteria 2 and 3 and 4 -> overall signal is GREEN

3.7.2 Secondary and other outcomes
All other outcomes will be summarised descriptively by trial group. Where appropriate, the effect size and 95% confidence intervals will be estimated with regression models adjusting for treatment group, baseline value (where applicable), and stratification variables. Further details regarding the secondary outcomes will be provided at a later date, (provisionally in a supplementary document) prior to database lock. Both absolute and relative effect sizes will be reported.

Complications will be summarised using:
- count/percentage of participants with any complication
- count/percentage of participants with any complication by grade
- count/percentage of participants with the highest grade of complication reported
- count/percentage of participants with any type of complication
- count of total complications.

4. STATISTICAL PRINCIPLES

4.1 Statistical Significance and Multiple Testing

See also the protocol section "The Level of Statistical Significance"

P-values will not be reported given the feasibility nature of the trial.[2] There will be no adjustment for multiple testing.[3] The 95% confidence intervals will be presented but regarded as nominal and descriptive.

4.2 Definition of Analysis Populations

See also the protocol section "Analysis populations"

All randomised and eligible participants that underwent surgery will be included in the main analysis on an intention-to-treat principle regardless of withdrawal or non-adherence. A per protocol analysis will include the subsample of intervention participants who achieved ≥5% weight loss from baseline to the day of surgery. The adverse event analysis will include the participants in the control group and the participants commencing the intervention in the intervention group. Participants who had their surgery less than 20 days since randomisation will be included in this analysis.

5. TRIAL POPULATION AND DESCRIPTIVE ANALYSES

This is a summary of flow of trial participants through the trial and baseline stratification, demographic and clinical characteristics of each group.

5.1 Representativeness of Study Sample and Patient Throughput

See also the protocol appendix "Study Flow Chart" and the appendices in this SAP.

The flow of participants through each stage of the trial, including numbers of participants randomly assigned, receiving intended treatment, completing the study protocol, and analysed for the primary outcome is provided following the appropriate guideline (e.g. CONSORT flow diagram). Protocol violations/deviations and information relating to the screening data including the number of ineligible patients randomised, together with reasons, information on number of participants screened, found to be ineligible (with reasons where available), refused to participate (with reasons where available) will be included as applicable.

The trial flowchart is given in the Appendices.

5.2 Withdrawal from treatment and/or follow-up

See also the protocol section "Informed Consent" and section "Early Discontinuation/Withdrawal of Participants"

Once enrolled and during the course of the study a participant may choose to withdraw early from the intervention at any time for any reason. This may happen for several reasons, including but not limited to:

- The occurrence of what the participant perceives as an intolerable AE.
- Inability to comply with study procedures
- Participant decision

The numbers (and percentages) of withdrawals will be reported by treatment group along with the reasons for these withdrawals. These will be summarised as per the table below.

Table 2. Details Of Withdrawals (And Reasons) Split By Treatment Group

Treatment	Patient ID	Date of withdrawal (DDMonYYYY)	Reasons for withdrawal
Intervention	ID 1		
	ID 2		
	…		
Control	ID 1		
	ID 2		
	…		

5.3 Baseline Characteristics

See also the protocol sections "Baseline Assessments" and the relevant tables in the SAP DDT

The baseline face-to-face assessment will be conducted during the screening visit at each site. Baseline characteristics are reported by treatment group, including the stratification/minimisation factors and any other variable deemed relevant.

Numbers (with percentages) for binary and categorical variables and mean (and standard deviation), or median (with lower and upper quartiles) for continuous variables will be presented; there will be no tests of statistical significance nor confidence intervals for differences between randomised groups on any baseline variable.

5.4 Unblinding

See also the protocol section "Blinding and code-breaking"

The nature of the study precludes blinding of participants and research nurses. Unblinding of the central research team and independent assessors of the post-operative complications (as per 3.2 and 3.6) may occur only following recommendations of the TSDMEC.

5.5 Treatment Adherence with Details of Intervention

See also the protocol sections "Protocol Deviations", "Study Monitoring" and "Serious Breaches"

The number of people in each arm receiving their randomised treatment will be reported. The adherence of the randomised participant to the intervention and control pre-surgery will be checked and reported as per the protocol. Adherence with the protocol and the SOPs and any post-surgery complications will be checked and reported as per the protocol.

5.6 Reliability

The REDCap database will incorporate range and logic checks. Manual checks may be carried on the data to ensure plausibility on the recognisance of the Trial Statistician.

6. ANALYSIS

6.1 Outcome Definitions

See also the protocol section "Synopsis"

The primary variables for this trial are referred to as "trial progression criteria" and a list of those primary outcomes is given below. The study also has secondary outcomes (morbidity, oncological outcomes, etc) which are not listed separately here as a comprehensive list can be found in the "synopsis" section of the protocol.

Table 3: a simple list of all primary variables and their measurement

Type	Objectives	Outcome Measures	Definition
Primary (Recruitment)	1a. Recruitment variable	Recruitment Rate	n of patients recruited per site per month
Primary (Recruitment)	1b. Recruitment variable	Number of sites open	Integer (0-n)
Primary (Recruitment)	1c. Recruitment variable	Total number of participants recruited	Integer (0-n)
Primary (Safety)	5. Safety profile	Based on related adverse events and expected related and unexpected related serious adverse events	Type, number of and percentage of each event category.
Primary (Engagement)	2. Engagement variable	Proportion of phone calls answered. This is defined as the "mean percentage of phone calls answered per participant"	Percentage (0-100%)
Primary (Adherence)	3. Adherence variable	Proportion of intervention participants with ≥5% weight loss from baseline to the day of surgery	Percentage (0-100%)
Primary (Retention)	4. Retention variable	% at final follow-up	Percentage (0-100%)

6.2 Analysis Methods

See section 3.7 "Statistical Analysis Outline"

6.3 Missing Data

See also the protocol section "Procedure For Accounting For Missing, Unused, And Spurious Data"

For the analysis of each of the progression criteria, the following will apply:

1. Recruitment: missing data are not applicable, so they will not be imputed.
2. Engagement: missing data are not applicable, as instances of expected consultations with which participants do not engage will be coded as "no engagement" rather than as missing.
3. Adherence: It is plausible that weight data may be missing. Missing data will be imputed using methodology deemed appropriate by the Trial Statistician given the magnitude of the missing data and any other factors deemed relevant at the time. This imputation is likely to be through baseline observation carried forward, because of (a) the short duration (~4 weeks) between the two time points (screening and admission) during which weight typically remains relatively stable, (b) we

anticipate a relatively small proportion of missing data as the follow-up visit happens on admission to hospital, and (c) the total size of the study is small.

4. Retention: missing data are not applicable, so they will not be imputed.

6.4 Sensitivity Analysis

No sensitivity analyses are planned. Should the data during the study begin to deviate from that expected (eg a non-nominal amount of missing data) then appropriate sensitivity analyses may be undertaken at the discretion of the Trial Statistician.

6.5 Pre-specified Subgroup Analysis

See also the protocol section "Description of the Statistical Methods"

No subgroup analyses are planned.

6.6 Supplementary/Additional Analyses and Outcomes

No supplementary analyses are planned.

6.7 Harms

Safety is an outcome variable and its analysis is detailed elsewhere in this document in sections such as the section "Statistical Analysis Outline" and others.

6.8 Health Economics and Cost Effectiveness (where applicable)

See also the protocol section "Health Economic analysis"

The health economics analysis plan will be reported separately.

7. VALIDATION OF THE PRIMARY ANALYSIS

See also the protocol section "Description Of The Statistical Methods"

To validate the primary outcomes a statistician not involved in the trial will independently repeat the analyses detailed in this SAP. The results will be compared and any unresolved discrepancies will be reported in the Statistical Report (See OCTRU SOP STATS-005 Statistical Report).

Table 4: list of outcome variables subject to checks by uninvolved statistician

Type	Subtype	Outcome variable (as defined in table 3)
Primary	2. Engagement variable	Proportion of phone calls answered
Primary	3. Adherence variable	Proportion of intervention participants with ≥5% weight loss from baseline to the day of surgery
Primary	4. Retention variable	Percentage at final follow-up
Primary	1a. Recruitment variable	Recruitment Rate

Primary	1b. Recruitment variable	Number of sites open
Primary	1c. Recruitment variable	Total number of participants recruited

8. SPECIFICATION OF STATISTICAL PACKAGES

Any specific statistical analysis will be carried out using appropriate validated statistical software such as STATA, SAS, SPLUS, or R. Any other analysis done by the Trial Statistician will be done by that software deemed appropriate by the Trial Statistician. The relevant package and version number of any software used will be recorded in the Statistical Report.

9. PUBLICATION

See also the protocol sections “Ethical And Regulatory Considerations”

This study will be conducted as part of the portfolio of trials in the Surgical Intervention Trials Unit (SITU), a Royal College of Surgeons of England specialist trials centre dedicated to evaluating surgical intervention at the University of Oxford. It will follow their Standard Operating Procedures ensuring compliance with the principles of Good Clinical Practice and the Declaration of Helsinki and any applicable regulatory requirements.

10. REFERENCES

1. Lewis M, Bromley K, Sutton CJ, et al. Determining sample size for progression criteria for pragmatic pilot RCTs: the hypothesis test strikes back! *Pilot Feasibility Stud* 2021;7(1):40. doi: 10.1186/s40814-021-00770-x [published Online First: 2021/02/05]
2. Eldridge SM, Chan CL, Campbell MJ, et al. CONSORT 2010 statement: extension to randomised pilot and feasibility trials. *Pilot Feasibility Stud* 2016;2:64. doi: 10.1186/s40814-016-0105-8
3. Li G, Taljaard M, Van den Heuvel ER, et al. An introduction to multiplicity issues in clinical trials: the what, why, when and how. *Int J Epidemiol* 2017;46(2):746-55. doi: 10.1093/ije/dyw320 [published Online First: 2016/12/28]
4. Lewis, M., Bromley, K., Sutton, C.J. et al. Determining sample size for progression criteria for pragmatic pilot RCTs: the hypothesis test strikes back!. Pilot Feasibility Stud 7, 40 (2021). , see link at https://pilotfeasibilitystudies.biomedcentral.com/articles/10.1186/s40814-021-00770-x, see also DOI at https://doi.org/10.1186/s40814-021-00770-x

APPENDIX: GLOSSARY OF ABBREVIATIONS

Table: Glossary of abbreviations

Abbr	Meaning
CARE	CARE feasibility randomised controlled trial
DMEC	Data Monitoring and Ethics Committee (DMEC)
RBP	Randomisation and Blinding Plan
RCT	Randomised Controlled Trial
RRAMP	Registration / Randomisation and Management of Product, a randomisation software for REDCap
SAP	Statistical Action Plan
SOP	Standard Operating Procedure
TMF	Trial Master File
TSC	The independent Trial Steering Committee (TSC)
TSDMEC	Trial Steering, Data Monitoring, and Ethics Committee (TSDMC)

APPENDIX: STUDY FLOWCHART

Figure 1: Trial Flowchart

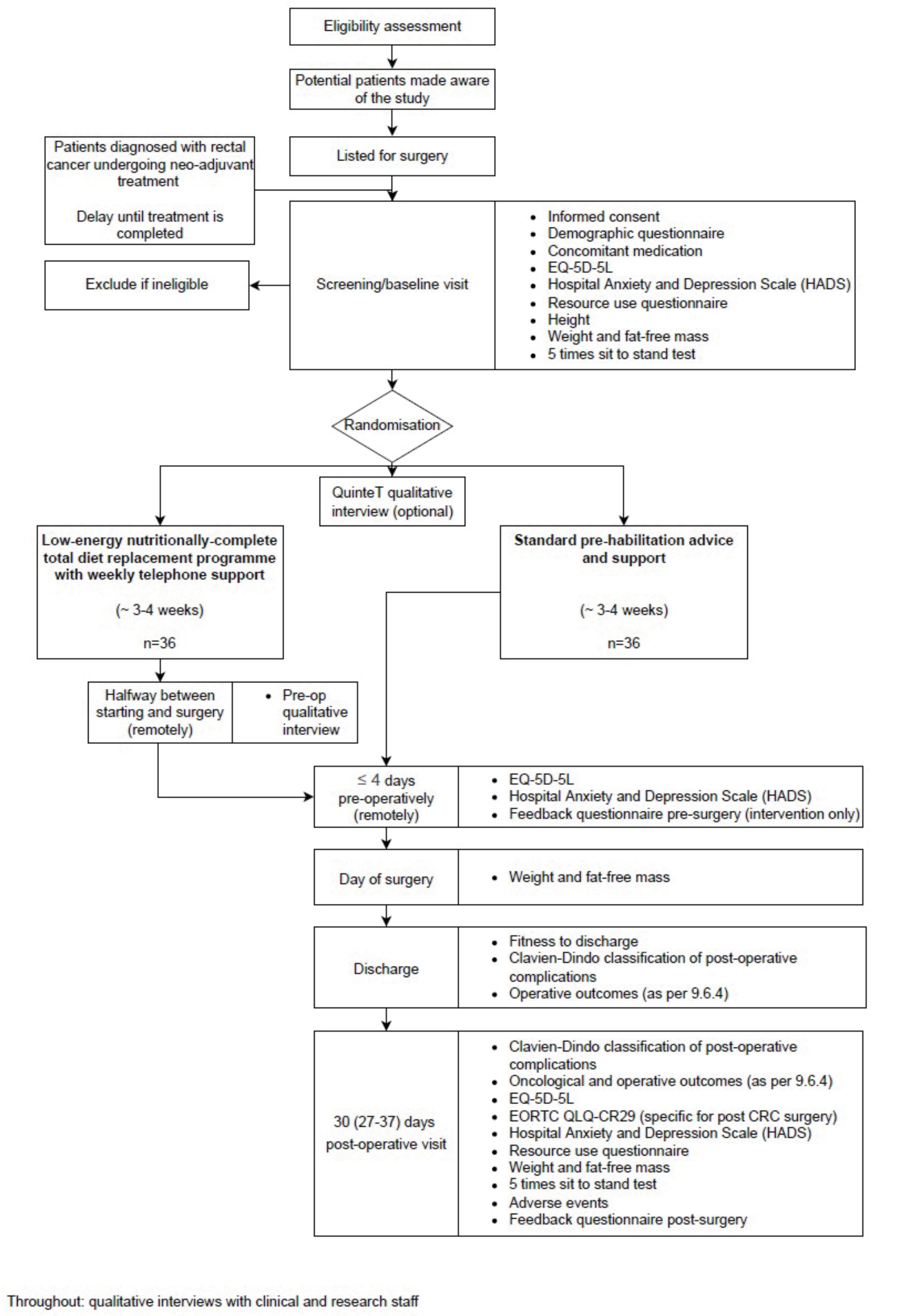

The trial flowchart is given in the Appendices. It was copied from the appendix "STUDY FLOW CHART" in the protocol and formed a standalone image called "Care Trial flowchart.jpg"

CARE

Pre-operative intentional weight loss to support post-operative recovery in patients with overweight and colorectal cancer: the CARE feasibility randomised controlled trial

(Short title: Could supported weight loss reduce bowel cancer surgery complications?)

Statistical Analysis Plan supplementary

Version v1.0 07Oct2024

Based on Protocol version v3.0 27Feb2023
Based on Statistical Analysis Plan (SAP) version v2.0 07Oct2024
Based on Statistical Analysis Plan - Data Definition and Tables (SAP-DD&T or SAP-DDT) version v1.0 30Sep2023

Trial registration: IRAS Project ID: 320173

Surgical Intervention Trials Unit (SITU)

CONTENTS

1. INTRODUCTION 4
2. OVERVIEW 4
3. DEFINITION OF POPULATION FOR ANALYSIS 4
4. USE OF MODELS 5
4.1 INPUT VARIABLES 5
4.2 OUTPUT VARIABLES 5
4.3 MODEL SELECTION FOR A GIVEN OUTCOME VARIABLE 6
5. LIST OF SECONDARY OUTCOMES 7
6. MORBIDITY 8
6.1 I. ANY MORBIDITY 8
6.2 II. MORBIDITY BY GRADE (I, II, IIIA, IIIB, IVA, IVB) 8
6.3 III. SURVIVAL (GRADE V) 9
6.4 IV MORBIDITY BY HIGHEST GRADE 9
6.5 V. NUMBER OF PEOPLE WITH NO MORBIDITY 9
6.6 VI. MORBIDITY BY TYPE (EG ARRYTHMIA, WOUND INFECTION, ETC) 9
7. ONCOLOGICAL OUTCOMES 9
7.1 IV. RESECTION MARGINS 9
7.2 V. RECURRENCE 9
7.3 VI. NEW PRIMARY/ SECONDARY CANCER 10
8. OPERATIVE OUTCOMES 10
8.1 VII. INTRAOPERATIVE BLOOD LOSS 10
8.2 VIII. OPERATIVE TIME 10
8.3 IX. CONVERSION TO OPEN SURGERY 10
8.4 X. SURGICAL SITE INFECTION 11
8.5 XI. STOMA RATES & COMPLICATIONS 11
8.6 XII. RADIOLOGICALLY-DEFINED ANASTOMOTIC LEAKS 11
8.7 XIII. TIME IN INTENSIVE CARE UNIT AND TIME IN HIGH DEPENDENCY UNIT 11
8.8 XIV. RE-OPERATION RATES 11
8.9 XV. RE-ADMISSION RATES 11
9. HOSPITAL STAY 11
9.1 XVI. LENGTH OF HOSPITAL STAY (FITNESS TO DISCHARGE) 12
9.2 XVII. DAYS ALIVE AND OUT OF HOSPITAL 12
10. ANTHROPOMETRY 12
10.1 XVIII. WEIGHT 12
10.2 XIX. FAT-FREE MASS 12
11. FITNESS 13
11.1 XX. TIME FOR SIT-TO-STAND TEST 13
12. HRQOL (HEALTH-RELATED QUALITY OF LIFE) 13
12.1 XXI. EQ-5D-5L 13
12.2 XXII. HADS (ANXIETY) 13
12.3 XXII. HADS (DEPRESSION) 14
12.4 XXIII. EORTC-QLQ-CR29 14
13. COSTS AND RESOURCE USE 14

14. ADVERSE EVENTS 14
14.1 XXVII. ADVERSE EVENTS 14
15. FURTHER CONSIDERATIONS ON THE ANALYTICAL APPROACH 15
15.1 MISSING DATA 15
15.2 OUTLIERS 15
15.3 SUBGROUP ANALYSES 15
15.3.1 HADS (anxiety) 15
15.3.2 HADS (depression) 16
15.4 SENSITIVITY ANALYSES 16
16. REFERENCES 16
17. APPENDIX: SCORING RULES 18
17.1 EQ-5D-5L: HRQOL USING PATIENT REPORTED OUTCOME MEASURE (PROMS) EQ-5D-5L 18
17.1.1 Summary index value 18
17.1.2 Visual Analogue Scale (VAS) 19
17.2 EORTC-QLQ-CR29 19
17.3 HADS 21
18. APPENDIX: SCREENSHOT OF EORTC-QLQ-CR29 QUESTIONNNAIRE 23
19. APPENDIX: SCREENSHOT OF HADS QUESTIONNAIRE 24
20. APPENDIX: LINEAR MIXED MODEL THEORY 25
20.1 LINEAR MIXED MODELS: LINKS 25
20.2 LINEAR MIXED MODELS: THE THEORY 25
20.3 LINEAR MIXED MODELS: WORKED EXAMPLE 25
21. APPENDIX: USE OF THE WORD "LINEAR" IN LINEAR MODELS 26
22. APPENDIX: LOOKUP TABLE 27
22.1 GLMS 27
22.2 NON-GLMS IF GLMS CANNOT COPE OR ARE INFERIOR 27
22.3 TIME-TO-EVENT MODELS 27
22.4 GREEN, JA (2021) 28
23. APPENDIX: CATEGORICAL, BINARY, CONTINUOUS 29
23.1 CONTINUOUS 29
23.2 CATEGORICAL AND BINARY 29
23.3 CATEGORICAL AND CONTINUOUS 29
23.4 BINARY 29
24. APPENDIX: ABSOLUTE VS RELATIVE 30
24.1 CONTINUOUS 30
24.2 CATEGORICAL AND BINARY 30
24.3 CATEGORICAL AND CONTINUOUS 30
24.4 BINARY 30
24.5 DISCRETE 30
25. APPENDIX: GENERALIZED LINEAR MODEL THEORY 31
26. APPENDIX: WORKED EXAMPLES OF MORBIDITY 31
26.1 WORKED EXAMPLE OF "ANY MORBIDITY" 31
26.2 WORKED EXAMPLE OF "MORBIDITY BY GRADE (I, II, IIIA, IIIB, IVA, IVB)" 32
26.3 WORKED EXAMPLE OF "MORBIDITY BY HIGHEST GRADE (I, II, IIIA, IIIB, IVA, IVB)" 32

1. INTRODUCTION

The CARE SAP includes this text

> *3.7.2 Secondary and other outcomes*
>
> *Further details regarding the secondary outcomes will be provided at a later date, (provisionally in a supplementary document) prior to database lock.*
>
> *Excerpt from CARE_SAP_v1.0_14Mar2023.docx*

This is that supplementary document.

2. OVERVIEW

The secondary outcomes will be summarised descriptively by trial group. If appropriate, the effect size and 95% confidence intervals will be estimated with regression models. Both absolute and relative effect sizes will be reported.

Complications will be summarised using one or more of the following:

- count/percentage of participants with any complication
- count/percentage of participants with any complication by grade
- count/percentage of participants with the highest grade of complication reported
- count/percentage of participants with any type of complication
- count of total complications.

3. DEFINITION OF POPULATION FOR ANALYSIS

Main analysis: All randomised participants that underwent surgery will be included in the main analysis on an intention-to-treat basis (ITT) regardless of withdrawal or non-adherence. An intention-to-treat basis assigns all randomised participants to their originally allocated group, whether they received their allocated randomised treatment arm approach or not, or dropped out or not. Participants who had their surgery less than 20 days since randomisation will be included in this analysis.

Per-protocol analysis: We will also conduct an analysis on a per-protocol basis. A per-protocol basis includes only those randomised participants who met all eligibility criteria, had no major deviations from the study procedures, and adhered to their assigned interventions. For the intervention group, this includes the participants who achieved ≥5% weight loss from baseline to the day of surgery. For participants in the usual care group, this excludes those who lost ≥5% weight loss from baseline to the day of surgery. Participants who had their surgery less than 20 days since randomisation will not be included in this analysis.

Adverse events: The adverse event analysis will include the participants in the control group and the participants commencing the intervention in the intervention group.

4. USE OF MODELS

4.1 Input variables

The SAP states that the mean and 95% confidence intervals for the secondary outcome variables will be estimated with adjusted regression models. Those adjustments will be our four input variables, specifically:

- treatment group. There are two treatment groups, the control and the intervention.
- baseline value (where applicable)
- time point (where applicable)
- stratification variables. The two stratification variables are performance status (0 vs 1-2) and age at diagnosis ((</≥70 years). These will be combined into one [1][2].

The extension from regression models to mixed models was not explicitly stated but is assumed. Such models will have fixed effects and may be further extended to include a random effect and/or an interaction. Details of those models include:

- The fixed effects are the adjustments
- The random effect is the participant/study ID
- The interaction is multiplicative. For example, an interaction between the treatment group and the time point (both of which are fixed) will be considered.

In models with interaction terms, the treatment effect will be the interaction term. In models without an interaction term, the treatment effect will be the treatment group.

4.2 Output variables

We expect to model all secondary outcome variables, except survival, recurrence, new primary/secondary cancer, re-admission rates, re-operation rates, costs and resource use, and adverse events. The final decision will depend on the available data. For example, if the counts for conversion to open surgery rates are very low, we will not model this outcome.

Therefore, we plan on analysing:

- Morbidity (five variables not including 2iii survival)
- Operative outcomes (seven variables not including re-operation and re-admission rates)
- Hospital stay (two variables)
- Anthropometry (two variables)
- Fitness (one variable)
- Health-related quality of life (HRQoL) (three variables)

That’s twenty variables equating to at least 20 models, as some of those variables will be further subdivided (eg HRQoL variable “EQ-5D-5L” has two subdivisions, index and VAS).

4.3 Model selection for a given outcome variable

See Appendix "Use Of The Word "Linear" In Linear Models" for an explanation of the use of the word "linear" even if the underlying curve is a higher polynomial

The output variables may be continuous or categorical and the input variables may be categorical or continuous. This would imply a logistic model (logistic regression or discriminant analysis) or a linear model (linear regression or ANOVA or ANCOVA or mixed) or a generalized model (GLM) approach. As GLMs are the most capable, we will use GLMs for our models where possible.

5. LIST OF SECONDARY OUTCOMES

Table: secondary outcomes

Objectives	Outcome Measures	Timepoint(s)	Type	Distribution
To report between-group differences in				
2. Morbidity	i. Any morbidity	i.-ii. Discharge and 30-days post-op	Binary	Binomial
	ii. Morbidity by grade (I, II, IIIa, IIIb, IVa, IVb)	ditto	Binary	Binomial
	iii. Survival (grade V)	iii: Discharge, 30-days post-op, 3 yrs	N/A: not model	
	iv Morbidity by highest grade	i.-ii. Discharge and 30-days post-op	Binary	Binomial
	v. Number of people with no morbidity	iii: Discharge, 30-days post-op, 3 yrs	Binary	Binomial
	vi. Morbidity by type (e.g., arrythmia, wound infection, etc)	i.-ii. Discharge and 30-days post-op	Binary	Binomial
3. Oncological outcomes	iv. Resection margins (involved Y/N)	iv. Discharge	Binary	Binomial
	v. Recurrence	v-vi: 3 years	N/A: not model	
	vi. New primary/ secondary cancer	ditto	N/A: not model	
4. Operative outcomes	vii. Intraoperative blood loss			
	Count/rate (transfused Y/N)	vii-ix: Discharge	Binary	Binomial
	units transfused (including untransfused)	ditto	Continuous	See lookup
	viii. Operative time	ditto	Continuous	See lookup
	ix. Conversion to open surgery	ditto	Binary	Binomial
	x. Surgical site infection	x-xiii: Discharge and 30-days post-op	Binary	Binomial
	xi. Stoma rates	ditto	Binary	Binomial
	xi. Stoma complications	ditto	Binary	Binomial
	xii. Radiologically -defined anastomotic leaks	ditto	Binary	Binomial
	xiii. Length of time in intensive care unit	ditto	Continuous	See lookup
	xiii. Length of time in high dependency unit	ditto	Continuous	See lookup
	xiv. Re-operation rates	xiv-xv: 30-days post-op and 3 years	N/A:not model	
	xv. Re-admission rates	ditto	N/A:not model	
5. Hospital stay	xvi. Length of hospital stay (fitness to discharge)	xvi: Discharge	Time-to-event	See lookup
	xvii. Days alive and out of hospital	xvii: 30-days post- operatively	Time-to-event	See lookup
6. Anthropometry	xviii. Weight	xviii-xix: Baseline, pre-operative assessment 4, and 30 days post-op	Continuous	See lookup
	xix. Fat-free mass (absolute kg)	ditto	Continuous	See lookup
	xix. Fat-free mass (relative %)	ditto	Continuous	See lookup
7. Fitness	xx. Time for sit-to-stand test	xx: Baseline, 30 days post-op	Continuous	See lookup
8. HRQoL	xxi. EQ-5D-5L (Index score)	xxi-xxii: Baseline, pre-operative assessment 3 and 30 days post-op	Continuous	See lookup
	xxi. EQ-5D-5L (VAS)	ditto	Continuous	See lookup
	xxii. HADS (anxiety) continuous	ditto	Continuous	See lookup
	xxii. HADS (depression) continuous	ditto	Continuous	See lookup
	xxiii. EORTC-QLQ-CR29			
	29 individual items	ditto	N/A: not model	
9. Costs & resource use	N/A - dealt with by health economics			
10. Adverse events	xxvii. Adverse events (excluding morbidity i-vi)	xxvii: Baseline, pre-operative assessment and 30 days post-op	N/A: not model	

Details of each variable are provided below.

6. MORBIDITY

Morbidity

#	Outcome Measures	Control	Intervention	Delta
2a	i. Any morbidity			
	At or prior to discharge	X	X	X
	Between discharge and 30 days	X	X	X
	Other	X	X	X
	Total	X	X	X
	At or prior to discharge<br>OR between discharge and 30 days	X	X	X
2b	ii. Morbidity by grade (I, II, IIIa, IIIb, IVa, IVb)			
	at least one Grade I	X	X	X
	.	X	X	X
	at least one Grade IVb	X	X	X
	Other	X	X	X
	Total	X	X	X
2c	iii. Survival (grade V)	X	X	X
2d	iv Morbidity by highest grade	X	X	X
2e	v. Number of people with no morbidity	X	X	X
2f	vi. Morbidity by type (eg arrythmia, wound infection, etc)			
	Type	X	X	X
	.	X	X	X
	Type	X	X	X
	Other	X	X	X
	Total	X	X	X

Note: the “other” categories are included for completeness and will be omitted if absent
Note: this is a list of patients, not complications. Because a patient can have more than one complication of different times/grades, an arm may not add to 100%. See appendices for worked examples

6.1 i. Any morbidity

This secondary outcome is reported regardless of timepoint. The information to calculate it is derived from the morbidity grade as specified on the CRFs Complications Grading 1 and Complications Grading 2. It is a binary variable and collectively a count/rate (0-n/%). The entry is defined as the number of patients with at least one complication divided by the number of patients.

For worked examples of this please see the appendices.

6.2 ii. Morbidity by grade (I, II, IIIa, IIIb, IVa, IVb)

This secondary outcome is reported by grade regardless of timepoint (i.e., a complication can occur at either discharge or 30-days follow-up). The information to calculate it is derived from the morbidity grade as specified on the CRFs Complications Grading 1 and Complications Grading 2. The entry for each grade X is is defined as the number of patients with at least one complication of grade X divided by the number of patients. It is a categorical variable. Each category is a count/rate (0-n/%) and will be modelled as a binary variable.

For worked examples of this please see the appendices.

6.3 iii. Survival (grade V)

This secondary outcome is reported for a single grade regardless of timepoint. The information to calculate it is derived from the morbidity grade as specified on the CRFs Complications Grading 1 and Complications Grading 2. It is a binary variable and collectively a count/rate (0-n/%). Given the expected low count, it is unlikely that this will be analysed statistically.

6.4 iv Morbidity by highest grade

As ii above. This secondary outcome is reported for a single grade regardless of timepoint. If a participant has one coded complication, the grade of that complication will count as the highest graded morbidity. If a participant has at least two complications, the highest grade will be counted for this variable. It is a categorical variable & each category is a count/rate (0-n/%). Each category will be modelled as a binary variable.

For worked examples of this please see the appendices.

6.5 v. Number of people with no morbidity

As i above.

6.6 vi. Morbidity by type (eg arrythmia, wound infection, etc)

This secondary outcome is reported regardless of timepoint. The information to calculate it is derived from the complication type as specified on the CRFs Complications Grading 1 and Complications Grading 2 (as derived from previous CRFs eg Hospital Stay and Discharge). It is a categorical variable & each category is a count/rate (0-n/%). Each category will be modelled as a binary variable.

7. ONCOLOGICAL OUTCOMES

Oncological Outcomes

#	Outcome Measures	Control	Intervention	Delta
3a	iv. Resection margins	X	X	X
3b	v. Recurrence	X	X	X
3c	vi. New primary/ secondary cancer	X	X	X

7.1 iv. Resection margins

This secondary outcome is reported at discharge. The information to calculate it is derived from the resection margins as recorded on the CRF Hospital Stay and Discharge. It is recorded as a continuous variable and a measurement of distance. It will be reduced to a binary measure (involved/not involved) and collectively a count/rate (0-n/%).

7.2 v. Recurrence

This secondary outcome is reported at 3 years. The information to calculate it is derived from the recurrence or metastatic type data on the CRF NBOCA. It is a binary variable and collectively a

count/rate (0-n/%). Due to low expected numbers, we do not plan an analysis for this. It will be depicted using survival curves.

7.3 vi. New primary/ secondary cancer

This secondary outcome will be collected at the 3-year follow-up. It is a binary variable and collectively a count/rate (0-n/%). Due to low expected numbers, we do not plan an analysis for this It will be depicted using survival curves.

8. OPERATIVE OUTCOMES

Operative Outcomes

#	Outcome Measures	Control	Intervention	Delta
4a	vii. Intraoperative blood loss			
	Count/rate (transfused Y/N)	X	X	X
	number of units transfused (including untransfused)	X	X	X
4b	viii. Operative time	X	X	X
4c	ix. Conversion to open surgery	X	X	X
4d	x. Surgical site infection	X	X	X
4e	xi. Stoma rates & complications	X	X	X
4f	xii. Radiologically-defined anastomotic leaks	X	X	X
4g	xiii. Time in intensive care unit	X	X	X
4g	xiii. Time in high dependency unit	X	X	X
4h	xiv. Re-operation rates	X	X	X
4i	xv. Re-admission rates	X	X	X

8.1 vii. Intraoperative blood loss

This outcome is reported using two variables: by the presence of intraoperative blood transfusion and by the number of units transfused (including the untransfused). The information to calculate it is derived from the fact of an intraoperative blood transfusion and the number of blood units used. These variables are recorded on the CRF Hospital Stay and Discharge.

The first variable is a binary variable and collectively a count/rate (0-n/%). The second variables are continuous variables and measurements of amount.

8.2 viii. Operative time

The information to calculate this outcome is derived from the total time taken for the procedure on the CRF Hospital Stay and Discharge. It is a continuous variable and a measurement of time.

8.3 ix. Conversion to open surgery

The information to calculate this outcome is derived from the operation performed as recorded on the CRF Hospital Stay and Discharge. It is a binary variable and collectively a count/rate (0-n/%).

8.4 x. Surgical site infection

The information to calculate this outcome is derived from the complications data on CRF Complications Grading 1 (as derived from previous CRFs eg Hospital Stay and Discharge). It is a binary variable and collectively a count/rate (0-n/%).

8.5 xi. Stoma rates & complications

The information to calculate each of the two subsets of this outcome is derived from the stoma/anastomosis data on the CRFs Hospital Stay and Discharge and Complications Grading 1. The existence of a stoma/anastomosis (Y/N) is derived from the "Stoma/anastomosis formed" CRF elements. The existence of complications (Y/N) is derived from the "Were there any stoma-specific complications?" CRF elements. Each one is a binary variable and collectively a count/rate (0-n/%).

8.6 xii. Radiologically-defined anastomotic leaks

The information to calculate this outcome is derived from the complications data on CRF Complications Grading 1 (as derived from previous CRFs eg Hospital Stay and Discharge). It is a binary variable and collectively a count/rate (0-n/%).

8.7 xiii. Time in intensive care unit and time in high dependency unit

The information to calculate these two lengths of time is derived from the ward and time details on the CRF Hospital Stay and Discharge. Each one is a continuous variable and a measurement of time.

8.8 xiv. Re-operation rates

The information to calculate this outcome is derived from the return to theatre data on the CRFs NBOCA data and Hospital Stay and Discharge. It is a binary variable and collectively a count/rate (0-n/%).

8.9 xv. Re-admission rates

The information to calculate this outcome is derived from the readmission data on the CRF Hospital Stay and Discharge. It is a binary variable and collectively a count/rate (0-n/%).

9. HOSPITAL STAY

#	Outcome Measures	Control	Intervention	Delta
5a	xvi. Length of hospital stay (fitness to discharge)	X	X	X
5b	xvii. Days alive and out of hospital	X	X	X

9.1 xvi. Length of hospital stay (fitness to discharge)

We will calculate the numbers of days of hospital stay using the date of admission and the date of discharge. The information to calculate it is derived from the admission and discharge data on the CRFs Admission and Hospital Stay and Discharge. It is a time-to-event variable and a measurement of time. Instructions for modelling time-to-event variables are given in the appendices.

9.2 xvii. Days alive and out of hospital

This secondary outcome is displayed by timepoint. We will calculate the number of days based on the dates of admission, discharge, and 30-day follow-up. The information to calculate it is derived from the discharge and other data on the CRFs Hospital Stay and Discharge and Anthropometry. It is a time-to-event variable and a measurement of time. Instructions for modelling time-to-event variables are given in the appendices.

The possibility of censorship must be noted here.

- If somebody has surgery on the 1st, discharged on the 10th and followed-up on the 30th, then on the 30th the state is known: at that date the days alive and out of hospital is known to be 20 days.
- But if somebody has surgery on the 1st, discharged on the 10th and followed-up on the 27th, then on the 30th the state is not known with certainty as they may have died in the meantime.

To prevent this censorship, we will seek confirmation from the local research teams that the person is still alive on the 30th.

10. ANTHROPOMETRY

#	Outcome Measures	Control	Intervention	Delta
6a	xviii. Weight	X	X	X
6b	xix. Fat-free mass	X	X	X

10.1 xviii. Weight

This secondary outcome is displayed by timepoint. The information to calculate it is derived from the weight data on the CRFs Anthropometry and Screening Visit. It is a continuous variable and a measurement of mass.

10.2 xix. Fat-free mass

This secondary outcome is displayed by timepoint. The information to calculate it is derived from the weight and body fat data on the CRFs Anthropometry and Screening Visit. It is a continuous variable and a measurement of mass. It is reported both as an absolute value (the fat-free mass in kg) and a relative value (the fat-free mass divided by the weight and depicted as a % of body mass).

11. FITNESS

#	Outcome Measures	Control	Intervention	Delta
7a	xx. Time for sit-to-stand test	X	X	X

11.1 xx. Time for sit-to-stand test

This secondary outcome is displayed by timepoint. The information to calculate it is derived from the sit-to-stand data on the CRF Sit To Stand Test. It is a continuous variable and a measurement of time.

12. HRQOL (HEALTH-RELATED QUALITY OF LIFE)

#	Outcome Measures	Control	Intervention	Delta
8a	xxi. EQ-5D-5L			
	Index score	X	X	X
	VAS	X	X	X
8b	xxii. HADS			
	• Anxiety (continuous)	X	X	X
	• Depression (continuous)	X	X	X
8c	xxiii. EORTC-QLQ-CR29			
	29 individual items	X	X	X

12.1 xxi. EQ-5D-5L

This secondary outcome is displayed by timepoint. The information to calculate it is derived from the CRF EQ-5D-5L. It is in two parts: a summary index value derived from the code, and a visual analogue scale score (VAS). Instructions for calculating these values are given in the appendices.

- The EQ-5D-5L summary index value ranges from less than 0 (indicating a health state worse than death) to 1 (indicating full health). Higher scores represent better health states.
- The EQ-5D-5L Visual Analogue Scale (VAS) ranges from 0 to 100. The maximum value is 100 and represents the best health you can imagine. The minimum value is 0 and represented the worst health you can imagine.

We will treat both variables as scores and continuous variables. The scoring rules are in the appendices.

12.2 xxii. HADS (anxiety)

This secondary outcome is displayed as both a continuous and categorical variable. The information to calculate it is derived from the CRF HADS and Pre-Surgery Questionnaire. It is a score. The scoring rules are in the appendices. The continuous version is detailed here. The other variants are detailed in the subgroup analysis.

- Continuous. The continuous version of this variable will be a number derived from that score. It will be modelled as a continuous variable

12.3 xxii. HADS (depression)

This secondary outcome is displayed as both a continuous and categorical variable . The information to calculate it is derived from the CRF HADS and Pre-Surgery Questionnaire. It is a score. The scoring rules are in the appendices. The continuous version is detailed here. The other variants are detailed in the subgroup analysis.

- Continuous. The continuous version of this variable will be a number derived from that score. It will be modelled as a continuous variable

12.4 xxiii. EORTC-QLQ-CR29

This secondary outcome is displayed by timepoint. The information to calculate it is derived from the CRF EORTC QLQ - CR29. It is a continuous variable and a score. The scoring rules are in the appendices. The score will be calculated for the 29 individual items. The 29 individual items will be descriptive only

13. COSTS AND RESOURCE USE

These outcomes will be calculated as part of the health economics section of the trial and are, therefore, not covered in this document.

14. ADVERSE EVENTS

#	Outcome Measures	Control	Intervention	Delta
10a	xxvii. Adverse events	X	X	X

14.1 xxvii. Adverse events

The information to calculate it is derived from CRF Adverse Events (any time). It is a categorical variable & each category is a count/rate (0-n/%).

#	Outcome Measures	Control	Intervention	Delta
10b	xxvii. Adverse events (by severity)			
	N of AEs in category Mild	X	X	X
	N of AEs in category Moderate	X	X	X
	N of AEs in category Serious	X	X	X
10c	xxvii. Adverse events (by seriousness)			
	N of AEs in category Serious	X	X	X
	N of AEs in category Non-serious	X	X	X
10d	xxvii. Adverse events (misc)			
	N of people with at least one AE	X	X	X
	N of people with at least one moderate AE	X	X	X
	N of people with at least one serious AE	X	X	X
	N of people with at least one SAE	X	X	X
	N of people with at least one moderate AE and/or SAE	X	X	X

15. FURTHER CONSIDERATIONS ON THE ANALYTICAL APPROACH

15.1 Missing data

Missing data will be checked and either corrected if wrong or left if legitimate. Missing data will not be imputed (but see sensitivity analysis). The amount of missing data will be quantified and analyses for variables with missing data will be noted as such.

Mixed effects models usually do not require imputation as they are usually robust to missing data (although this depends if missing data is missing at random or not).

15.2 Outliers

Outliers will be checked and either corrected if wrong or left if legitimate. Outliers will not be removed. Analyses for variables with outliers will be noted as such. There are various methods of identifying an outlier, including the "3SD's beyond the mean" heuristic. We will use the "1.5IQR rule": outliers will be those points below Q1 − 1.5IQR, or above Q3 + 1.5IQR [6].

15.3 Subgroup Analyses

As stated in the section named "Statistics and Analysis" in the protocol, no formal subgroup analyses are planned. However, subgroup analyses requested after the protocol was written are permissible on an exploratory and descriptive basis at the discretion of the Trial Statistician. These subgroup analyses are referred to as "informal". With the exception of the stratification factors (see sensitivity analysis below) those informal analyses are descriptive and without the need to calculate or report p-values.

15.3.1 <u>HADS (anxiety)</u>

#	Outcome Measures	Control	Intervention	Delta
8b	xxii. HADS			
	• Anxiety (is +ve change since baseline >= MCID, Y/N)	X*	X*	X†
	• Anxiety (is -ve change since baseline >= MCID, Y/N)	X*	X*	X†

† = difference between arms: ie compare the control change since baseline to the intervention change since baseline.
* = this is a count and percentage of all the patients in that arm whose change since baseline exceeds the MCID.

- Anxiety (is +ve change since baseline >= MCID, Y/N)
  - This is the proportion of participants with a positive change at the pre-op 3 assessment since baseline of at least 1.7 points as the minimum clinically important difference (Y/N). This is a binary variable.

- Anxiety (is -ve change since baseline >= MCID, Y/N)
  - This is the proportion of participants with a negative change at the pre-op 3 assessment since baseline of at least 1.7 points as the minimum clinically important difference (Y/N). This is a binary variable.

The minimum clinically important difference for HADS is 1.7 points, see Lemay KR, et al [8]

15.3.2 HADS (depression)

#	Outcome Measures	Control	Intervention	Delta
8b	xxii. HADS			
	• Depression (is +ve change since baseline >= MCID, Y/N)	X*	X*	X†
	• Depression (is -ve change since baseline >= MCID, Y/N)	X*	X*	X†

† = difference between arms: ie compare the control change since baseline to the intervention change since baseline.
* = this is a count and percentage of all the patients in that arm whose change since baseline exceeds the MCID.

As HADS (anxiety) above.

15.4 Sensitivity Analyses

Sensitivity analyses will be done on at least the following:

- **Per-protocol analysis.** The per-protocol analysis will repeat the above models using the per protocol population as defined in section 3.

- **Stratification factors.** The two stratification factors are performance status (“0” vs “1-2”) and age at diagnosis (“< 70 years” and “≥70 years”). For sensitivity analysis we will do the following:
  - Repeat the models with an interaction term between age at diagnosis (“< 70 years” and “≥70 years”) and treatment. This will be expanded to “…and treatment and time” if time is an input variable, see below.
  - Repeat the models with an interaction term between performance status (“0” vs “1-2”) and treatment. This will be expanded to “…and treatment and time” if time is an input variable, see below.
  - Models measuring the state at one timepoint (eg discharge) or the state change between two timepoints will not need a time variable. Models measuring the state at two or more timepoints (eg baseline vs admission vs 30-days post-op) will need a time variable. In such cases, the interaction above will include the time variable. The paper at [7] is an example of this.

- **Other.** Other sensitivity analyses may be conducted if deemed necessary by the Trial Statistician.

16. REFERENCES

- [1] Kahan. 2013. 'Adjusting for multiple prognostic factors in the analysis of randomised trials', BMC Med Res..” This is one of the papers mentioned by CI Koutoukidis, see https://bmcmedresmethodol.biomedcentral.com/articles/10.1186/1471-2288-13-99 and https://doi.org/10.1186/1471-2288-13-99
- [2] Paper 3: Kahan, B. C., and T. P. Morris. 2012. 'Improper analysis of trials randomised using stratified blocks or minimisation', Stat Med, 31: 328-40. This is another of the papers mentioned by CI Koutoukidis, see https://onlinelibrary.wiley.com/doi/epdf/10.1002/sim.4431
- [3] “Intention-To-Treat (ITT) vs. Per-Protocol (PP) analysis: what to choose?”, Clinfo.eu, see https://www.clinfo.eu/itt-vs-pp/

- [4] ICH Topic E9, "Statistical Principles for Clinical Trials, step 5: Note For Guidance On Statistical Principles For Clinical Trials (CPMP/ICH/363/96)", European Medicines Agency, September 1998, see https://www.ema.europa.eu/en/documents/scientific-guideline/ich-e-9-statistical-principles-clinical-trials-step-5_en.pdf
- [5] Green, J. A. (2021). Too many zeros and/or highly skewed? A tutorial on modelling health behaviour as count data with Poisson and negative binomial regression. Health Psychology and Behavioral Medicine, 9(1), 436–455. https://doi.org/10.1080/21642850.2021.1920416
- [6] Carnegie Mellon University, Statistical Computing, Fall 2013, instructor Prof. Cosma Shalizi, see https://www.stat.cmu.edu/~cshalizi/statcomp/13/labs/05/lab-05.pdf
- [7] Ardoin et al (2014), “Secondary analysis of APPLE study suggests atorvastatin may reduce atherosclerosis progression in pubertal lupus patients with higher C reactive protein”. Ann Rheum Dis. 2014 Mar;73(3):557-66. doi: 10.1136/annrheumdis-2012-202315. Epub 2013 Feb 22. PMID: 23436914; PMCID: PMC4104199. See https://www.ncbi.nlm.nih.gov/pmc/articles/PMC4104199/
- [8] Ref for MCID: Lemay KR, Tulloch HE, Pipe AL, Reed JL. “Establishing the Minimal Clinically Important Difference for the Hospital Anxiety and Depression Scale in Patients With Cardiovascular Disease.” J Cardiopulm Rehabil Prev. 2019 Nov;39(6):E6-E11. doi: 10.1097/HCR.0000000000000379. PMID: 30489438. See the link at https://pubmed.ncbi.nlm.nih.gov/30489438/

17. APPENDIX: SCORING RULES

Where available, scoring rules are given below. To reduce duplication, rules that are explicated in the tables above are not duplicated here.

17.1 EQ-5D-5L: HRQoL using Patient Reported Outcome Measure (PROMs) EQ-5D-5L

- *Sources: https://euroqol.org/wp-content/uploads/2021/01/EQ-5D-5LUserguide-08-0421.pdf*
- *Online calculator: https://euroqol.org/wp-content/uploads/2020/12/ENG_value-set_STATA.txt*

17.1.1 Summary index value

Each variable has a tick box, where each dimension has five response levels: 1=no problems, 2=slight problems, 3=moderate problems, 4=severe problems, 5=unable to /extreme problems.

The answers are concatenated into a code (eg “12213”) and converted to a utility code, either manually or via code such as STATA. The scoring rules to translate the code into a utility code can be gotten from the user guide “EQ-5D-5L User Guide” at the EQ-5D website at https://euroqol.org/wp-content/uploads/2021/01/EQ-5D-5LUserguide-08-0421.pdf

Conversely, the scoring rules for translating the code to an utility code can be deduced by looking at the STATA code available https://euroqol.org/wp-content/uploads/2020/12/ENG_value-set_STATA.txt. That dataset gives us this lookup table:

variable	value	weight
mobility	1	0
mobility	2	0.058
mobility	3	0.076
mobility	4	0.207
mobility	5	0.274
selfcare	1	0
selfcare	2	0.05
selfcare	3	0.08
selfcare	4	0.164
selfcare	5	0.203
activity	1	0
activity	2	0.05
activity	3	0.063
activity	4	0.162
activity	5	0.184
pain	1	0
pain	2	0.063
pain	3	0.084
pain	4	0.276

pain	5	0.335
anxiety	1	0
anxiety	2	0.078
anxiety	3	0.104
anxiety	4	0.285
anxiety	5	0.289

So a code of "12213" would translate as 1x0+2x0.05+2x0.05+1x0+3x0.104 = 0+0.1+0.1+0+0.312=0.512

The EQ-5D-5L summary index value ranges from less than 0 (indicating a health state worse than death) to 1 (indicating full health). Higher scores represent better health states.

17.1.2 Visual Analogue Scale (VAS)

The EQ-5D-5L Visual Analogue Scale (VAS) ranges from 0 to 100.

- Maximum Value: 100, representing the best health you can imagine.
- Minimum Value: 0, representing the worst health you can imagine.

17.2 EORTC-QLQ-CR29

- *Sources: https://drive.google.com/file/d/16j3pr9bEVYBRa2tMp6B_2QLnxJ85kaDP/view https://www.eortc.org/app/uploads/sites/2/2018/08/Specimen-CR29-English-2.1.pdf https://www.ncbi.nlm.nih.gov/pmc/articles/PMC6541702/*
- *Online calculator: https://score.awellhealth.com/calculations/eortc_qlq_cr29*

The EORTC QLQ-CR29 is a patient-reported outcome measure to evaluate health-related quality of life among colorectal cancer (CRC) patients in research and clinical practice. For the scoring rules see here: https://drive.google.com/file/d/16j3pr9bEVYBRa2tMp6B_2QLnxJ85kaDP/view .

The questionnaire includes 29 items, numbered 31 to 59. They are divided into two categories: items about symptoms (gastrointestinal, urinary, pain and others) and items about functions (sexual, body image and others) that are associated with CRC and its treatments. There are separate versions of items 49 to 54 for people with and without stomas, and item 55 is only for patients with a stoma. Items 56 and 57 are for men only, and items 58 and 59 are for women only.

All the QLQ-CR29 questions except one (item 48 requires a yes or no answer) have four possible answers. They are

- Not at all (1)
- A little (2)
- Quite a bit (3)
- Very much (4)

Those four levels are a Likert scale of four response categories and are linearly converted into a scale from 0 to 100 (ie 0,33,67,100 or 100,67,33,0). Patients are asked to indicate their symptoms during the past week except for the sexuality questions, which ask for the past four weeks.

Table: EORTC-QLQ-CR29 Functional Scales (0 is bad, 100 is good)

Scale	Scale name	Number of items	Item #	Question #	100 score at
Body_Image	BI	3	15,16,17	45,46,47	Not at all (1)
Anxiety	ANX	1	13	43	Not at all (1)
Weight	WEI	1	14	44	Not at all (1)
Sexual_interest_(men)	SEXM	1	26	56	Very much (4) [1]
Sexual_interest_(women)	SEXW	1	28	58	Very much (4) [1]

Table: EORTC-QLQ-CR29 Symptom scales (0 is good, 100 bad)

Scale	Scale name	Number of items	Item #	Question #	100 score at
Urinary_frequency	UF	2	1,2	31,32	Very much (4)
Blood_and_mucus_in_stool	BMS	2	8,9	38,39	Very much (4)
Stool_frequency*	SF	2	22,23	52,53	Very much (4)
Urinary_incontinence	UI	1	3	33	Very much (4)
Dysuria	DY	1	4	34	Very much (4)
Abdominal_pain	AP	1	5	35	Very much (4)
Buttock_pain	BP	1	6	36	Very much (4)
Bloating	BF	1	7	37	Very much (4)
Dry_mouth	DM	1	10	40	Very much (4)
Hair_loss	HL	1	11	41	Very much (4)
Taste	TA	1	12	42	Very much (4)
Flatulence*	FL	1	19	49	Very much (4)
Faecal_incontinence*	FI	1	20	50	Very much (4)
Sore_skin*	SS	1	21	51	Very much (4)
Embarrassment*	EMB	1	24	54	Very much (4)
Stoma_care_problems	STO	1	25	55	Very much (4)
Impotence	IMP	1	27	57	Very much (4)
Dyspareunia	DYS	1	29	59	Very much (4)

Table: EORTC-QLQ-CR29 Questions

#	#	Code	Question	Levels	Scale	Scale name	100 at	NB
31	1	EORTCQLQCR29_Q31	Did you urinate frequently during the day during the past week?	4	Symptom	Urinary frequency	Very much (4)	
32	2	EORTCQLQCR29_Q32	Did you urinate frequently during the night during the past week?	4	Symptom	Urinary frequency	Very much (4)	
33	3	EORTCQLQCR29_Q33	Have you had any unintentional release (leakage) of urine during the past week?	4	Symptom	Urinary incontinence	Very much (4)	
34	4	EORTCQLQCR29_Q34	Did you have pain when you urinated during the past week?	4	Symptom	Dysuria	Very much (4)	
35	5	EORTCQLQCR29_Q35	Did you have abdominal pain during the past week?	4	Symptom	Abdominal pain	Very much (4)	
36	6	EORTCQLQCR29_Q36	Did you have pain in your buttocks/anal area/rectum during the past week?	4	Symptom	Buttock pain	Very much (4)	
37	7	EORTCQLQCR29_Q37	Did you have a bloated feeling in your abdomen during the past week?	4	Symptom	Bloating	Very much (4)	
38	8	EORTCQLQCR29_Q38	Have you had blood in your stools during the past week??	4	Symptom	Blood and mucus in stool	Very much (4)	
39	9	EORTCQLQCR29_Q39	Have you had mucus in your stools during the past week??	4	Symptom	Blood and mucus in stool	Very much (4)	
40	10	EORTCQLQCR29_Q40	Did you have a dry mouth during the past week?	4	Symptom	Dry mouth	Very much (4)	
41	11	EORTCQLQCR29_Q41	Have you lost hair as a result of your treatment during the past week?	4	Symptom	Hair loss	Very much (4)	
42	12	EORTCQLQCR29_Q42	Have you had problems with your sense of taste during the past week?	4	Symptom	Taste	Very much (4)	
43	13	EORTCQLQCR29_Q43	Were you worried about your health in the future during the past week?	4	Functional	Anxiety	Not at all (1)	
44	14	EORTCQLQCR29_Q44	Have you worried about your weight during the past week?	4	Functional	Weight	Not at all (1)	

45	15	EORTCQLQCR29_Q45	Have you felt physically less attractive as a result of your disease or treatment during the past week?	4	Functional	Body Image	Not at all (1)	
46	16	EORTCQLQCR29_Q46	Have you been feeling less feminine/masculine as a result of your disease or treatment during the past week?	4	Functional	Body Image	Not at all (1)	
47	17	EORTCQLQCR29_Q47	Have you been dissatisfied with your body during the past week?	4	Functional	Body Image	Not at all (1)	
48	18	EORTCQLQCR29_Q48	Do you have a stoma bag (colostomy/ileostomy)?	2	n/a	n/a	Very much (4)	
49	19	EORTCQLQCR29_Q49_NO_STOMA	Have you had unintentional release of gas/flatulence from your back passage during the past week?	4	Symptom	Flatulence*	Very much (4)	[1]
49	19	EORTCQLQCR29_Q49_STOMA	Have you had unintentional release of gas/flatulence from your stoma bag during the past week?	4	Symptom	Flatulence*	Very much (4)	[2]
50	20	EORTCQLQCR29_Q50_NO_STOMA	Have you had leakage of stools from your back passage during the past week?	4	Symptom	Faecal incontinence*	Very much (4)	[1]
50	20	EORTCQLQCR29_Q50_STOMA	Have you had leakage of stools from your stoma bag during the past week?	4	Symptom	Faecal incontinence*	Very much (4)	[2]
51	21	EORTCQLQCR29_Q51_NO_STOMA	Have you had sore skin around your anal area during the past week?	4	Symptom	Sore skin*	Very much (4)	[1]
51	21	EORTCQLQCR29_Q51_STOMA	Have you had sore skin around your stoma during the past week?	4	Symptom	Sore skin*	Very much (4)	[2]
52	22	EORTCQLQCR29_Q52_NO_STOMA	Did frequent bowel movements occur during the day during the past week?	4	Symptom	Stool frequency*	Very much (4)	[1]
52	22	EORTCQLQCR29_Q52_STOMA	Did frequent bag changes occur during the day during the past week?	4	Symptom	Stool frequency*	Very much (4)	[2]
53	23	EORTCQLQCR29_Q53_NO_STOMA	Did frequent bowel movements occur during the night during the past week?	4	Symptom	Stool frequency*	Very much (4)	[1]
53	23	EORTCQLQCR29_Q53_STOMA	Did frequent bag changes occur during the night during the past week?	4	Symptom	Stool frequency*	Very much (4)	[2]
54	24	EORTCQLQCR29_Q54_NO_STOMA	Did you feel embarrassed because of your bowel movement during the past week?	4	Symptom	Embarrassment*	Very much (4)	[1]
54	24	EORTCQLQCR29_Q54_STOMA	Did you feel embarrassed because of your stoma during the past week?	4	Symptom	Embarrassment*	Very much (4)	[2].
55	25	EORTCQLQCR29_Q55_STOMA	Did you have problems caring for your stoma during the past week?	4	Symptom	Stoma care problems	Very much (4)	[2]
56	26	EORTCQLQCR29_Q56	To what extent were you interested in sex during the past four weeks?	4	Functional	Sexual interest (men)	Very much (4)	[3]
57	27	EORTCQLQCR29_Q57	Did you have difficulty getting or maintaining an erection during the past four weeks?	4	Symptom	Impotence	Very much (4)	[3]
58	28	EORTCQLQCR29_Q58	To what extent were you interested in sex during the past four weeks?	4	Functional	Sexual interest (women)	Very much (4)	[4]
59	29	EORTCQLQCR29_Q59	Did you have pain or discomfort during intercourse during the past four weeks?	4	Symptom	Dyspareunia	Very much (4)	[4]

[1] Answer this question only when you DO NOT have a stoma bag.
[2] Answer this question only when you have a stoma bag.
[3] For men only.
[4] For women only

17.3 HADS

- *Sources: https://www.ncbi.nlm.nih.gov/pmc/articles/PMC5700594, https://academic.oup.com/occmed/article/64/5/393/1436876, https://score.awellhealth.com/calculations/hads/documentation*
- *Online calculator: https://score.awellhealth.com/calculations/hads*
- *Note: HADS is a generated score in the SITU RedCap for this project.*

The HADS questionnaire measures hospital anxiety or depression level.

It has fourteen questions divided into two subscales. There are seven questions for one subscale (depression) and seven questions for the other (anxiety). Scoring for each item ranges from zero to

three, with three denoting highest anxiety or depression level. So depression ranges from 0 (good) to 21 (bad), and anxiety ranges from 0 (good) to 21 (bad). A score of more than 8 means considerable symptoms of anxiety or depression.

Table: items on the questionnaire that relate to anxiety

QUESTIONS	ANSWERS	SCORE
I feel tense or wound up	Most of the time; A lot of the time; From time to time, occasionally; Not at all	3-0
I get a sort of frightened feeling as if something awful is about to happen	Very definitely and quite badly; Yes, but not too badly; A little, but it doesn't worry me; Not at all	3-0
Worrying thoughts go through my mind	A great deal of the time; A lot of the time; From time to time, but not too often; Only occasionally	3-0
I can sit at ease and feel relaxed	Definitely; Usually; Not often; Not at all	0-3
I get a sort of frightened feeling like 'butterflies' in the stomach	Not at all; occasionally; Quite often; Very often	0-3
I feel restless as I have to be on the move	Very much indeed; Quite a lot; Not very much; Not at all	3-0
I get sudden feelings of panic	Very often indeed; Quite often; Not very often; Not at all	3-0

Table: items on the questionnaire that relate to depression

QUESTIONS	ANSWERS	SCORE
I still enjoy the things I used to enjoy	Definitely as much; Not quite so much; Only a little; Hardly at all	0-3
I can laugh and see the funny side of things	As much as I always could; Not quite so much now; Definitely not so much now; Not at all	0-3
I feel cheerful	Not at all; Not often; Sometimes; Most of the time	3-0
I feel as if I am slowed down	Nearly all the time; Very often; Sometimes; Not at all	3-0
I have lost interest in my appearance	Definitly; I don't take as much care as I should; I may not take quite as much care ; I take just as much care as ever	3-0
I look forward with enjoyment to things	As much as I ever did; Rather less than I used to; Definitely less than I used to; Hardly at all	0-3
I can enjoy a good book or radio or TV program	Often; Sometimes; Not often; Very seldom	0-3

18. APPENDIX: SCREENSHOT OF EORTC-QLQ-CR29 QUESTIONNNAIRE

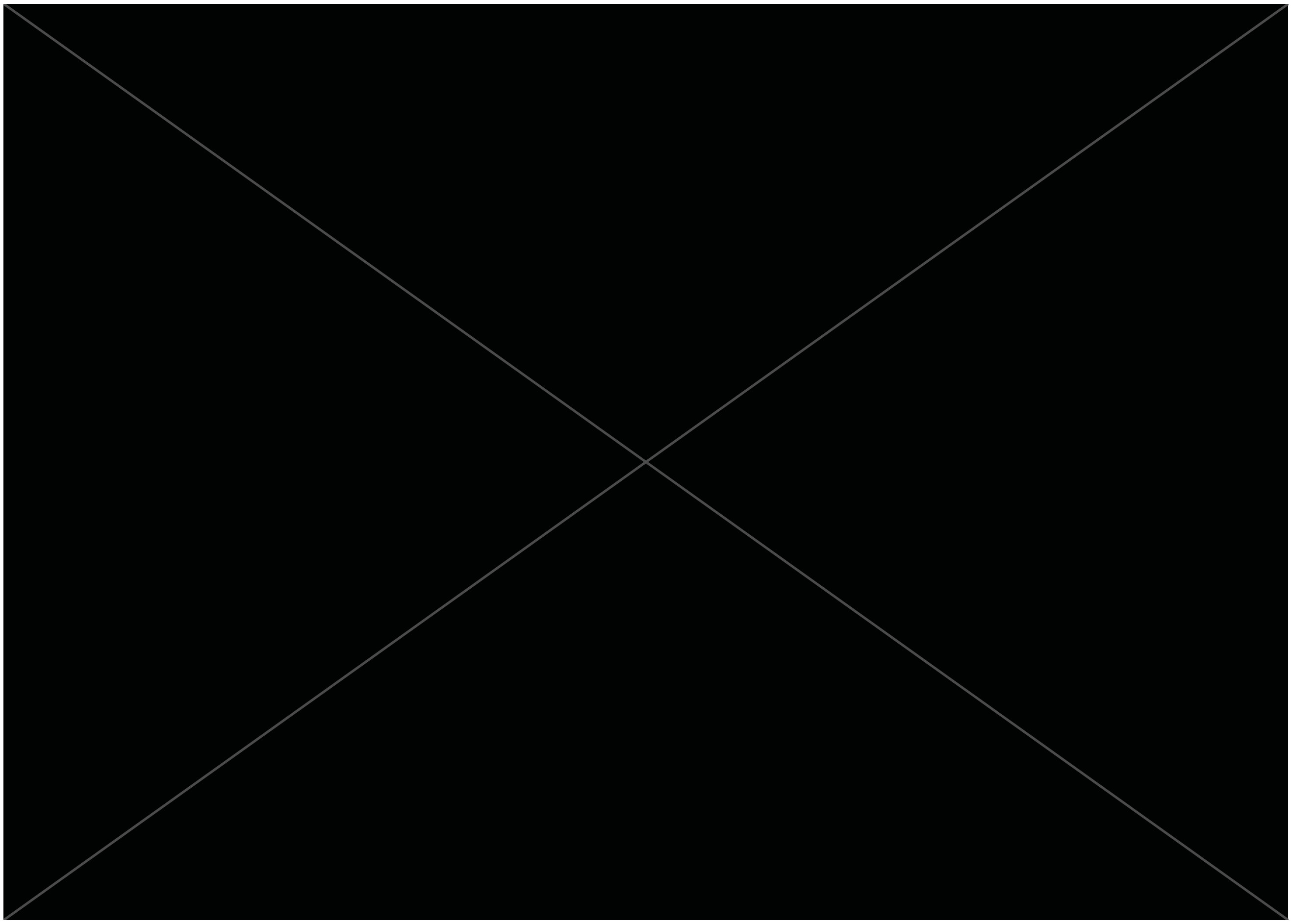

19. APPENDIX: SCREENSHOT OF HADS QUESTIONNAIRE

[redacted]

20. APPENDIX: LINEAR MIXED MODEL THEORY

20.1 Linear mixed models: links

See https://stats.oarc.ucla.edu/other/mult-pkg/introduction-to-linear-mixed-models/

20.2 Linear mixed models: the theory

The formula is:

y=xb+zu+e

where

Symbol	Description	Type
ny	number of values of the outcome variable	Scalar
np	number of predictor variables	Scalar
nj	number of groups	Scalar
nq	number of random intercepts per group	Scalar
y	outcome variable	ny x 1 vector
x	predictor variables aka fixed-effects variables,	ny x np matrix
b	fixed-effects regression coefficients (parameters)	np x 1 vector
z	random effects variables	ny x nj matrix
u	random-effects regression coefficients	nj x 1 vector
e	residuals (error)	ny x 1 vector

20.3 Linear mixed models: worked example

Consider this scenario:

Arm (J=2) indexed by the j subscript each see nj patients. So our grouping variable is the arm. Each arm sees 36 patients. The total number of patients is the sum of the patients seen by each arm. In our example, N=72, so 72 patients were seen by two arms. Our outcome is a continuous variable, output weight. Furthermore we have 3 fixed effects predictors, baseline value, timepoint, and stratification variable, plus a fixed intercept and one random intercept (q=1) for each of the J=2 arms. For simplicity, we are only going to consider random intercepts. We will let every other effect be fixed for now. The reason we want any random effects is because we expect that output weights within arms may be correlated.

So the formula is

y=xb+zu+e

That gives us this table below:

Symbol	Description	Type
ny	number of values of the outcome variable	72
np	number of predictor variables	4
nj	number of groups	2
nq	number of random intercepts per group	1
y	outcome variable	72 x 1 vector
x	Four predictor variables aka fixed-effects variables,<br>• Fixed intercept<br>• baseline value,<br>• timepoint,<br>• stratification variable	72 x 4 matrix
b	fixed-effects regression coefficients (parameters)	4 x 1 vector
z	One random effects variable<br>• Random intercept	72 x (1x2) matrix
u	random-effects regression coefficients	2 x 1 vector
e	residuals (error)	72 x 1 vector

21. APPENDIX: USE OF THE WORD “LINEAR” IN LINEAR MODELS

The word “linear” in “linear models” refers to the parameters, not the variables: it can cope with quadratic equations

- https://www.quora.com/Why-is-linear-regression-called-linear-if-we-can-use-quadratic-equations-in-our-model-which-gives-a-curved-line
- https://www.quora.com/Why-is-it-still-called-linear-regression-even-if-we-choose-a-polynomial-of-a-higher-degree-for-the-hypothesis

22. APPENDIX: LOOKUP TABLE

The type of a variable can be used to deduce the variable distribution - for example a variable measuring strength may have a Gamma distribution and a frequency variable may have a Poisson. For the avoidance of doubt we shall graph it as well.

When the variable type and distribution is known, the lookup tables below can be used to deduce which model is needed and which code can be used to do that. Stata code is shown for convenience, although SAS or R code would also suffice. See the Stata help documentation for more details

22.1 GLMs

#	Variable Type	Distribution	Link Fn	Example Stata Code†††
1	Continuous (Normal)	Gaussian	Identity	glm depvar indepvars, family(gaussian) link(identity)
2	Continuous (SPC) †	Inverse Gaussian	Power -2	glm depvar indepvars, family(igaussian) link(power -2)
3	Binary	Binomial (multiple) or Bernoulli (single)	Logit	glm depvar indepvars, family(binomial) link(logit)
4	Count/Rate (Poisson)	Poisson	Log	glm depvar indepvars, family(poisson) link(log)
5	Count/Rate (NB)	Negative binomial	Log	glm depvar indepvars, family(nbinomial) link(log)
6	Continuous (SPC) †	Gamma	Power -1	glm depvar indepvars, family(gamma) link(power -1)
6	Continuous (Time)	Gamma?	Power -1?	glm depvar indepvars, family(gamma) link(power -1)
99	Time-to-event	Exponential††	Log	glm depvar indepvars, family(exponential) link(log)

† SPC = Skewed positive continuous variable.
†† Not available in Stata. Code shown is synthetic. GLMs in this case would require another language
††† For a full list of families, link functions in Stata and their valid combinations, please see Stata help. Note that "inverse" is the same as "power -1", "inverse squared" is the same as "power -2", and so on

22.2 Non-GLMs if GLMs cannot cope or are inferior

Variable Type	Distribution	Link Function	Example Stata Code
Binary (Bernoulli)	Bernoulli	Logit (log-odds)	logit depvar indepvars, family(binomial) link(logit)
Binary (Probit)	Probit	Probit (CDF of standard normal)	probit depvar indepvars, family(binomial) link(probit)
Categorical (Nominal)	Multinomial	Logit	mlogit depvar indepvars, basecategory(1)
Categorical (Ordinal)	Categorical	Probit	ologit depvar indepvars
Categorical (Ordinal)	Cumulative logit	Logit (log-odds)	ologit depvar indepvars, family(ordinal) link(logit)
Categorical (Ordinal)	Proportional odds	Logit (log-odds)	ologit depvar indepvars, family(ordinal) link(logit)
Continuous (Normal)	Normal	Identity (linear)	reg depvar indepvars, family(gaussian) link(identity)
Count/Rate (NB)	Negative binomial	Log	nbreg depvar indepvars, family(nbinomial) link(log)
Count/Rate (Poisson)	Poisson	Log	poisson depvar indepvars, family(poisson) link(log)

22.3 Time-to-event models

Variable Type	Details	Example Stata Code
Time-to-event	Cox Proportional Hazards Model	stset depvar failure(eventvar==1)†<br>stcox indepvars
Time-to-event	Parametric survival models, named distribution	stset depvar failure(eventvar==1)†<br>streg indepvars, distribution(named distn) ††

† depvar would be the time-to-event variable, eventvar=0 if censored, 1 if not
†† named distributions currently supported by Stata are exponential, Weibull, Gompertz, lognormal, loglogistic, and ggmma (short for generalized gamma). "Streg" can handle frailty and accelerated-failure-time models as well as proportional hazards models. See Stata help on "streg" for details

22.4 Green, JA (2021)

The paper Green, JA (2021) (see[5] for more details) also gave a guide for which variable types would need which families. This guide can be used in tandem with the tables above

Variable Type	Family
Count variables	Poisson
Count variables	Negative Binomial
Count variables with excess zeros	Zero-Inflated Poisson
Count variables with excess zeros	Zero-Inflated Negative Binomial
Count variables with excess zeros	Hurdle Models
Overdispersed count variables	Quasi-Poisson
Overdispersed count variables	Quasi-Negative Binomial
Overdispersed count variables	Quasi-Binomial
Continuous skewed variables	Gamma

23. APPENDIX: CATEGORICAL, BINARY, CONTINUOUS

Consider the following synthetic example designed for illustration purposes

Table: synthetic example

ID	Weight	EORTC QLQ-CR29 Category	Not at all	A bit	Quite a bit	StomaBagY/N
yyyyyyy1	4.4	AbdominalPain	1	0	0	1
yyyyyyy2	4.4	AbdominalPain	1	0	0	0
yyyyyyy3	4.2	BloodInStools	0	1	0	0
yyyyyyy4	2.1	DryMouth	0	0	1	1
yyyyyyy5	4.2	DryMouth	0	0	1	1

23.1 Continuous

This can be considered as a continuous variable that can be modelled with one model

Continuous variable

Continuous variable	Mean Weight
Weight	3.86

23.2 Categorical and binary

Or as a categorical variable with a count/rate that can be modelled with three models

Categorical and binary: a variable with three categories, each with a count/rate

Category	Count/rate
AbdominalPain	2/5
BloodInStools	1/5
DryMouth	2/5

23.3 Categorical and continuous

Or as a categorical variable with three categories, each holding a continuous variable. This can be modelled with one model

Categorical and continuous: a variable with three categories, each with a continuous variable

Category	Mean Weight
AbdominalPain	4.4
BloodInStools	4.2
DryMouth	3.15

23.4 Binary

Or as a binary variable that can be modelled with one model

Binary variable

Binary variable	Count/rate
StomaBagY/N	3/5

24. APPENDIX: ABSOLUTE VS RELATIVE

The results of the study are depicted as absolute and relative values, although not all variables will be depicted as both. As for the meaning of "absolute" and "relative", we take our lead from the protocol. It says that "*Continuous variables will be summarised using means, standard deviations, and 95% confidence intervals. Medians with interquartile ranges will be presented where appropriate. Categorical variables will be summarised using counts and percentages.*"

For examples of the absolute vs relative distinction for effect size, please see "Appendix: Worked Examples Of Morbidity"

24.1 Continuous

As per protocol this is a mean and standard deviation (SD). Our synthetic example looks like this.

Continuous variable

Continuous variable	Mean Weight (SD)
Weight	3.86 (1.64)

24.2 Categorical and binary

As per protocol, this is a count and percentage. Our synthetic example looks like this.

Categorical and binary: a variable with three categories, each with a count/rate

Category	Count/rate
AbdominalPain	2 (40%)
BloodInStools	1 (10%)
DryMouth	2 (40%)
Total	5 (100%)

24.3 Categorical and continuous

As per protocol this is a mean and standard deviation (SD). Our synthetic example looks like this.

Categorical and continuous: a variable with three categories, each with a continuous variable

Category	Mean Weight (SD)
AbdominalPain	4.4 (1.6)
BloodInStools	4.2 (0)
DryMouth	3.15 (1.1)

24.4 Binary

As per protocol this is a mean and standard deviation (SD). Our synthetic example looks like this.

Binary variable

Binary variable	Count/rate
StomaBagY/N	3 (60%)
Total	5 (100%)

24.5 Discrete

As per protocol, this is a median and interquartile range. We do not have a synthetic example for this.

25. APPENDIX: GENERALIZED LINEAR MODEL THEORY

In statistics, a generalized linear model (GLM) is a flexible generalization of ordinary linear regression (LR).

A ordinary linear regression connects the input variables to the dependent variable via one function. But a GLM uses two functions: the variance function and the link function. This allows the linear model to be related to the response variable via a link function and allows the magnitude of the variance of each measurement to be a function of its predicted value.

Table: distributions and link functions for a GLM

Distribution	Variance Function	Typical Link Function
Normal	$V(\mu) = 1$	Identity: $g(\mu) = \mu$
Binomial	$V(\mu) = \mu(1-\mu)$	Logit: $g(\mu) = \log(\mu/(1-\mu))$
Poisson	$V(\mu) = \mu$	Log: $g(\mu) = \log(\mu)$
Gamma	$V(\mu) = \mu^2$	Inverse: $g(\mu) = 1/\mu$ or Log: $g(\mu) = \log(\mu)$
Inverse Gaussian	$V(\mu) = \mu^3$	Inverse squared: $g(\mu) = 1/\mu^2$
Negative Binomial	$V(\mu) = \mu + \alpha\mu^2$	Log: $g(\mu) = \log(\mu)$

26. APPENDIX: WORKED EXAMPLES OF MORBIDITY

The display of morbidity data can be difficult because problems arise when the morbidity has subdivisions or the data is displayed by patient not complications. To make things easier we provide worked examples below.

Firstly, let us build a synthetic example. This is an example designed for illustration purposes and it looks like this:

Synthetic example part 1: Control arm. three patients, four complications

Patient ID	Complication morbidity grade	Date of complication	Date of discharge	Date difference
xxxxxxx1	I	01Aug2024	15Aug2024	-14
xxxxxxx1	I	01Aug2024	15Aug2024	-14
xxxxxxx2	IIIa	01Aug2024	15Jul2024	17
xxxxxxx3	IV	01Aug2024	15Jun2024	47

Synthetic example part 2: Intervention arm. three patients, four complications

Patient ID	Complication morbidity grade	Date of complication	Date of discharge	Date difference
Xxxxxxx4	I	01Aug2024	15Jul2024	17
Xxxxxxx4	II	01Aug2024	15Jul2024	17
Xxxxxxx5	IIIa	01Aug2024	15Jul2024	17
Xxxxxxx6	IIIb	01Aug2024	15Jun2024	47

26.1 Worked example of “Any morbidity”

The synthetic example gives us this table. It is a list of patients, not complications

#	Outcome Measures	Control	Intervention	Delta
2a	i. Any morbidity			
	At or prior to discharge	1 (33%)	0 (0%)	-1 (-33%)
	Between discharge and 30 days	1 (33%)	2 (66%)	+1 (+33%)
	Other	1 (33%)	1 (33%)	-
	Total	3 (100%)	3 (100%)	-
	At or prior to discharge OR between discharge and 30 days	2 (66%)	2 (66%)	-

26.2 Worked example of "Morbidity by grade (I, II, IIIa, IIIb, IVa, IVb)"

The synthetic example gives us this table. It is a list of patients, not complications

#	Outcome Measures	Control	Intervention	Delta
2b	ii. Morbidity by grade (I, II, IIIa, IIIb, IVa, IVb)			
	At least one Grade I	1 (33%)	1 (33%)	-
	At least one Grade II	0 (0%)	1 (33%)	+1 (+33%)
	At least one Grade III	1 (33%)	2 (66%)	+1 (+33%)
	At least one Grade IV	1 (33%)	0 (0%)	-
	Other	0 (0%)	0 (0%)	-
	Total	3 (100%)	3 (100%)	-

Note: because a patient can have more than one complication, this example does not add to 100%

26.3 Worked example of "Morbidity by highest grade (I, II, IIIa, IIIb, IVa, IVb)"

The synthetic example gives us this table. It is a list of patients, not complications

#	Outcome Measures	Control	Intervention	Delta
2d	iv Morbidity by highest grade			
	Number of patients whose highest grade is Grade I	1 (33%)	0 (0%)	-1 (-33%)
	Number of patients whose highest grade is Grade II	0 (0%)	1 (33%)	+1 (+33%)
	Number of patients whose highest grade is Grade III	1 (33%)	2 (66%)	+1 (+33%)
	Number of patients whose highest grade is Grade IV	1 (33%)	0 (0%)	-
	Other	0 (0%)	0 (0%)	-
	Total	3 (100%)	3 (100%)	-

Note: because a patient can only have one highest grade, this example adds to 100%
