# Supplementary material for: Preoperative Weight Loss in Patients With Excess Weight and Colorectal Cancer: The CARE Feasibility Randomized Clinical Trial
Source: JAMA Netw Open. 2025 Dec 8;8(12):e2547126. doi: 10.1001/jamanetworkopen.2025.47126 (PMC12687096; doi:10.1001/jamanetworkopen.2025.47126)
Supplement: Supplement 2. — eAppendix 1. Changes to inclusion/exclusion criteria eFigure 1. Location of recruiting hospitals and annual case load per NHS Trust of elective major colorectal cancer resections without distant metastasis eTable 1. Pre-defined progression criteria to a definitive trial eAppendix 2. Allocation concealment, blinding, and statistical analysis eAppendix 3. Cost-utility modelling methods eTable 2. Justification of model parameters eFigure 2. Cost-effectiveness model of the intervention eAppendix 4. Recruitment eFigure 3. Planned vs actual recruitment rate (excluding participants who were withdrawn and replaced) (1 site closed early due to lack of recruitment) eTable 3. Demographic and operative characteristics eTable 4. Staging and clinical characteristics eAppendix 5. Reasons for intervention discontinuation eAppendix 6. Usual care group changes in relevant diet and physical activity behaviours eAppendix 7. Intervention evaluation eFigure 4. Intervention evaluation eTable 5. Oncologic and operative outcomes eTable 6. Complications by type (intention-to-treat) eTable 7. Weight, body composition, physical function, and quality of life eTable 8. Sensitivity analysis of the number and proportion of participants with minimally clinically important increase and reduction in anxiety and depression (by ≥1.7 points) between baseline and admission by group eAppendix 8. Adverse events eFigure 5. Complications by group based on the per protocol analysis eTable 9. Complications by type and group based in the per-protocol analysis eTable 10. Per-protocol analysis of anthropometric and quality of life outcomes eFigure 6. Interaction of quality of life (EQ-5D visual analogue scale), timepoint, and age eTable 11. P-values of interaction terms from sensitivity analysis stratified by age and performance status eTable 12. Subgroup analysis by colon (n=55) or rectum (n=16) in the intention-to-treat population eFigure 7. Incremental cost-effectiveness plane eFigure 8. Cost-effectiveness acc [file jamanetwopen-e2547126-s002.pdf]

Supplemental Online Content

Koutoukidis DA, Jebb SA, Reynolds S, et al. Preoperative weight loss in patients with excess weight and colorectal cancer: the CARE feasibility randomized clinical trial. *JAMA Netw Open*. 2025;8(12):e2547126. doi:10.1001/jamanetworkopen.2025.47126

eAppendix 1. Changes to inclusion/exclusion criteria
eFigure 1. Location of recruiting hospitals and annual case load per NHS Trust of elective major colorectal cancer resections without distant metastasis
eTable 1. Pre-defined progression criteria to a definitive trial
eAppendix 2. Allocation concealment, blinding, and statistical analysis
eAppendix 3. Cost-utility modelling methods
eTable 2. Justification of model parameters
eFigure 2. Cost-effectiveness model of the intervention
eAppendix 4. Recruitment
eFigure 3. Planned vs actual recruitment rate (excluding participants who were withdrawn and replaced) (1 site closed early due to lack of recruitment)
eTable 3. Demographic and operative characteristics
eTable 4. Staging and clinical characteristics
eAppendix 5. Reasons for intervention discontinuation
eAppendix 6. Usual care group changes in relevant diet and physical activity behaviours
eAppendix 7. Intervention evaluation
eFigure 4. Intervention evaluation
eTable 5. Oncologic and operative outcomes
eTable 6. Complications by type (intention-to-treat)
eTable 7. Weight, body composition, physical function, and quality of life
eTable 8. Sensitivity analysis of the number and proportion of participants with minimally clinically important increase and reduction in anxiety and depression (by ≥1.7 points) between baseline and admission by group
eAppendix 8. Adverse events
eFigure 5. Complications by group based on the per protocol analysis
eTable 9. Complications by type and group based in the per-protocol analysis
eTable 10. Per-protocol analysis of anthropometric and quality of life outcomes
eFigure 6. Interaction of quality of life (EQ-5D visual analogue scale), timepoint, and age
eTable 11. P-values of interaction terms from sensitivity analysis stratified by age and performance status

© 2025 Koutoukidis DA et al. *JAMA Network Open.*

eTable 12. Subgroup analysis by colon (n=55) or rectum (n=16) in the intention-to-treat population
eFigure 7. Incremental cost-effectiveness plane
eFigure 8. Cost-effectiveness acceptability curve for the reference case analysis
eTable 13. Incremental cost-effectiveness ratio (ICER) based on the reference case and sensitivity analyses
eReferences

This supplemental material has been provided by the authors to give readers additional information about their work.

© 2025 Koutoukidis DA et al. *JAMA Network Open.*

eAppendix 1. Changes to inclusion/exclusion criteria

We revised three exclusion criteria while recruitment was ongoing: (a) vegan meal replacement products became available so patients following a vegan diet became eligible, (b) we excluded those patients on insulin that also had a previous episode of diabetic ketoacidosis as a precaution due to the adjustment of insulin dose at the start of the intervention and (c) we excluded patients at risk of intestinal obstruction as a precaution because constipation, a side effect of the intervention, may increase this risk. No participant in the screening log was reported as excluded due to exclusion criterion (b) above. There were n=13 who were excluded due to risk of intestinal obstruction as per the CONSORT diagram.

eFigure 8. Location of recruiting hospitals and annual case load per NHS Trust of elective major colorectal cancer resections without distant metastasis

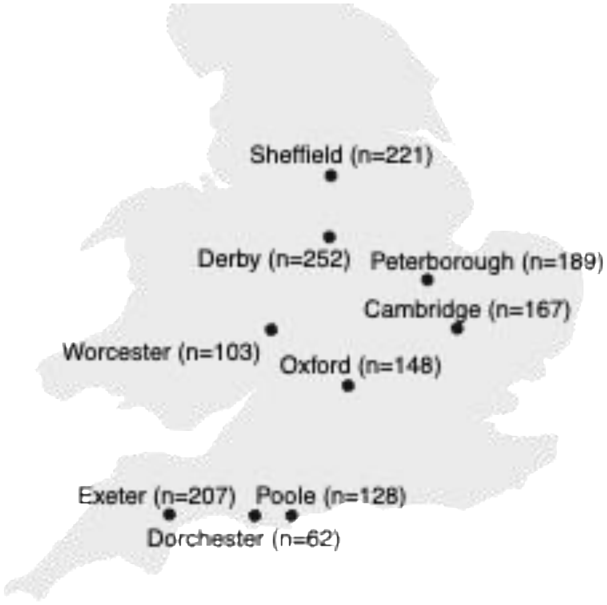

© 2025 Koutoukidis DA et al. *JAMA Network Open.*

eTable 14. Pre-defined progression criteria to a definitive trial

Sufficient levels of		Criterion Decision	**Green** <u>**Progress**</u>	**Amber** <u>**Progress with changes**</u>		**Red** <u>**Stop**</u>
Recruitment	1a	Rate (n of patients per site per month)[1]	≥0.75	0.46-0.74	Progress by adding sites.	≤0.45
	1b	Number of sites open	≥6 sites	3-5		≤2
	1c	Total N participants recruited	72	44-71		≤43
Engagement	2	Proportion of phone calls answered	≥75%	51-74%	Progress if process evaluation can recommend improvements.	≤50%
Adherence	3	Proportion of intervention participants with ≥5% weight loss from baseline to the day of surgery[2]	≥60%	36-59%		≤35%
Retention	4	% at final follow-up	≥85%	66-84%		≤65%
Safety	5	Safety profile	Based on related adverse events and on related expected and related unexpected serious adverse events. Adjudicated by the Data Monitoring and Ethics Committee.			

[1]Adjusted for centre size/participant pool based on the annual reported elective colorectal cancer surgeries without metastasis in the NBOCA data.

[2]Non-adherence was defined as <2% weight loss from baseline to the day of surgery.

© 2025 Koutoukidis DA et al. *JAMA Network Open.*

eAppendix 2. Allocation concealment, blinding, and statistical analysis

Allocation concealment

Allocation concealment was achieved as randomisation occurred after the baseline visit, the randomisation algorithm was unmodifiable and concealed from investigators and the local research teams, and the local research teams had no access to the existing number of participants randomised to each arm at the point they were randomising the next participant.

Blinding

Owing to the nature of the intervention, the participants could not be blinded. The surgeons who graded the postoperative complications were blinded to the group allocation, operating surgeons, weight and body composition changes, sit-to-stand tests, adverse events that were not classed as complications (e.g., adverse events prior to surgery), and questionnaires. Due to staffing and local pathways, it was not always possible to blind the operating surgeon or research staff to conduct the assessment of complications at follow-up.

Statistical analysis

A statistical analysis plan for the progression criteria (primary outcome) was published ahead of recruitment commencing and a supplementary statistical analysis plan for the secondary outcomes was published ahead of database lock on the ISRCTN trial registration page. Progression criteria are presented as mean ± standard deviations or percentages, as appropriate. Secondary outcomes with data at three timepoints were analysed using mixed effects models with participants as random effects and stratification factors, treatment arm, timepoint, and a multiplicative interaction between treatment and timepoint as fixed effects. The treatment effect was the interaction term. Secondary outcomes with data at two timepoints were analysed using regression models with the treatment arm, baseline value, and stratification factors as independent variables. The analysis followed the intention-to-treat approach, in which all eligible patients undergoing surgery were included regardless of treatment engagement or adherence. Sensitivity analyses included (a) per-protocol analysis including and excluding intervention and usual care participants, respectively, achieving ≥5% weight loss from baseline to the day of surgery and (b) analysis by levels of each of the two stratification factors. Due to mainly operating theatre availability, five participants (one in intervention and four in usual care) had their operation earlier than the 20-day window. Following the recommendations of the trial steering committee, these were included in the intention-to-treat but not the per-protocol analysis. As a post-hoc analysis, we looked at the

© 2025 Koutoukidis DA et al. *JAMA Network Open.*

proportion of participants in each group that had a positive or negative change in either anxiety or depression until surgery of at least the minimum clinically important difference of 1.7 points.[1] We also converted post-hoc the Clavien-Dindo grading to the Comprehensive Complication Index (CCI), which is a more sensitive endpoint for clinical trials,[2] and analysed using the approach described above with a Tweedie distribution that takes into account zero-inflated data. Following peer-review, we conducted subgroup analyses of complications, operative time, and length of stay by anatomical region (colon/rectum). Given the small numbers, it was not meaningful to analyse time spent in high dependency and intensive care units. Data were primarily analysed using STATA v15.1.

© 2025 Koutoukidis DA et al. *JAMA Network Open.*

eAppendix 3. Cost-utility modelling methods

Hypothetical subject and setting

We modelled a cohort of patients of an average age of 70 years undergoing curative elective colorectal cancer resection in a UK secondary or tertiary hospital. We did not specify further characteristics, such as tumour type, operation type, comorbidities, to allow for the model inputs to be applicable to existing literature and for the model estimates to be generalisable.

Intervention

We conducted an incremental cost-utility analysis of the intervention against standard pre-operative care. Based on the intention-to-treat trial data, we assumed that the intervention would lead to 6% weight loss between intervention start and surgery. This amount of pre-operative weight loss has been associated with a ~30% reduction in postoperative mortality after bariatric surgery.[3]

Prehabilitation interventions before colorectal cancer surgery focusing on increasing physical activity have been shown to reduce morbidity by ~20-40%.[4,5] We assumed that weight loss and physical activity share similar potential mechanisms through which they exert their effects to reduce morbidity (e.g., better glucose regulation and better physical fitness based on the improvements in physical fitness observed in the trial). Therefore, the estimates of morbidity reduction after physical activity could be applicable after intentional weight loss. We conservatively estimated a 20% reduction in postoperative complications.

Outcomes

The primary outcome of this analysis was the incremental cost-effectiveness ratio (ICER) between the two groups. We compared the ICER to the conventional willingness-to-pay threshold of £20,000 per QALY of the National Institute for Health and Care Excellence.[6] Additional outcomes included the expected total incremental costs in Pound sterling (£) and the incremental intervention effectiveness express in quality-adjusted life years (QALYs). Both costs and utilities were discounted at the standard 3.5% rate.[6]

Perspective and time horizon

We adopted a UK NHS and personal social services perspective, as this provides a measure of QALYs that is comparable across settings and meaningful for policy makers.[6] We chose a

© 2025 Koutoukidis DA et al. *JAMA Network Open.*

lifetime time horizon to capture the lifetime costs and utilities of each option. We used 30 yearly cycles with the average age at the time of surgery being 70 years based on the median age of diagnosis of colorectal cancer.

Model

We developed hybrid model with a decision-tree and Markov model components. The decision-tree model was for the first 30 days postoperatively, as most complications happen within the first month postoperatively. After that, patients transitioned between states based on a multivariable probabilistic Markov model as detailed in Figure 1. We assumed a 30-day mortality rate (omr) based on the latest national average.[7] After 30 days, people who did not have any complications could transition to developing longer-term complications (cr2) over the initial 5 years with no transition to a complication state after that. We based this estimate on complications that would lead to re-admission and therefore confer significant healthcare costs. Among those with complications, complication could resolve (cresolvedr) whereby patients transitioned to a state of having their complications resolved. From there, complication could re-occur (rcr) within the first 5 years and, as above, we estimated that these would lead to re-admission. We inflated standard UK mortality risk and yearly transition probabilities by published standardized mortality ratios to reflect excess mortality risk of non-metastatic colorectal cancer survivors compared with the general population.[8]

Model Validation

An experienced clinician provided input into the model structure and reviewed the parameter estimates that were sourced from the literature to establish face validity.

Measurement and valuation of transition probabilities, costs, and QALYs

Where possible, estimates were informed by (a) data from systematic reviews and meta-analyses in colorectal cancer, (b) data in a UK or, where not available, from Western high-income countries. We aimed to use average values across all colorectal cancer resections and surgical approaches to allow for a generalised estimate. We also aimed to minimise considering data from complications that do not require medical intervention (e.g., Clavien-Dindo grade 1 complications) and hence have smaller effect on QALYs and costs.

Data on the cost of complications were sourced from UK-specific analyses.[9] They were converted to pound sterling using the exchange rate at the time of reporting and inflated to 2023 based on latest NHS Cost inflation index. The cost of the intervention was based on

© 2025 Koutoukidis DA et al. *JAMA Network Open.*

micro-costing the CARE intervention using averages based on the actual length of the intervention, training, time needed for training, the number of dietetic consultations, recorded length of each consultation, administrative time, medical input, and approximate number of meal replacement products that participants had. The cost of initial operation was assumed to be the same between groups.

QALYs were estimated based on EQ-5D-5L values from a UK-based longitudinal study exploring the complications of patients undergoing major abdominal surgery. We conservatively assumed that the utility value of those with complications will be based on those with a Clavien-Dindo grade 2 complication and those returning to a state of resolved complications will have a utility value equivalent to those with a grade 1 complication, as there is typically remaining sequalae long-term.[10]

Probabilistic analyses

Transition probabilities and utility values followed a beta distribution in the probabilistic model. Costs followed a gamma distribution. We assumed parameter estimates were independent of each other and were sampled according to their respective distribution over 10,000 iterations. Beta and gamma distributions were derived using the method of moments. For the cost of complications, we assumed a 15% standard error as standard.[11] We used cost-effectiveness acceptability curves to estimate decision making uncertainty.[12]

Sensitivity analyses of the modelling

We conducted sensitivity analyses

Multivariable

a) By sex and age (60 and 80 years)

Univariable

b) Based on a 15% and 25% relative reduction in the initial postoperative complications
c) based on the expected intensity and costs of the intervention (£430) instead of the actual ones (£375).
d) based on utility value returning to equivalent of no complications when complications resolve.
e) based on the rate of complications re-occuring being 14% (instead of 19%).

© 2025 Koutoukidis DA et al. *JAMA Network Open.*

eTable 15. Justification of model parameters

Model parameter	Parameter value	Reference	Population description	Sample size	Comparator values
Operative mortality rate (omr)	2.4%	NBOCA[7]	England and Wales national average	35,779	2.4% died within 90 days postop
Complications rate in usual care (cr)	31%	CARE feasibility trial	Adults with BMI ≥28kg/m^2 planned for curative elective colorectal cancer resection.<br>Mean BMI: 35 kg/m^2, age: 64 years, 61% male<br>52% anterior resection, 55% laparoscopic procedure	71	31% had ≥1 grade 2 complication within 30 days postop
		Fung 2017[13]	Systematic review and meta-analysis of 12 studies examining laparoscopic colorectal cancer resection in obesity vs no obesity.	4,540	Presence of any morbidity as defined in each study.<br>Patients with obesity: 35%<br>Patients without obesity: 20%
		Yang 2015[14]	Systematic review and meta-analysis of 4 studies examining laparoscopic colorectal cancer resection in visceral obesity vs no obesity	659	Presence of any morbidity:<br>Patients with visceral obesity: 30%<br>Patients with no obesity: 18%
		Cakir 2015[15]	Systematic review and meta-analysis of 7 studies examining colorectal cancer resection without chemo/radiotherapy in CT-assessed visceral obesity vs no obesity	957	Presence of any morbidity:<br>Patients with visceral obesity: 34%<br>Patients with no obesity: 20%
		PREPARE-ABC trial collaborative[16]	Adults planned for curative elective colorectal cancer resection.<br>Randomised to physical activity or usual care pre-op.<br>Mean BMI: 28kg/m^2, age: 67 years, 70% male.<br>60% anterior resection, 85% laparoscopic procedure	181	43% had ≥1 grade 2 complication<br>52% of participants had ≥1 complication within 30 days postop
		Molenaar 2023[5]	Adults planned for curative elective colorectal cancer resection.<br>Randomised to physical activity or usual care pre-op.<br>Mean BMI: 27kg/m^2, age: 69 years, 55% male.<br>16% low anterior resection, 93% laparoscopic- or robotic-assisted surgery	128 in the usual care group	30% in the usual care group had ≥1 severe complication with CCI>20 (definition similar to ≥1 grade 2 complication)
		Leeds 2021[11]	Modelling of a 65-year-old patient with left-sided, stage II colon cancer eligible for surgical resection with ≥1 high-risk pre-operative comorbidity.	N/A	30.3% had ≥1 postoperative complication within 90 days postop that

© 2025 Koutoukidis DA et al. *JAMA Network Open.*

Model parameter	Parameter value	Reference	Population description	Sample size	Comparator values
			Estimate based on literature and expert opinion		would be likely to have meaningful impact on costs or quality of life
		ACS NSQIP Surgical risk calculator[17]	ACS NSQIP 44140 - Colectomy partial with anastomosis[1]	N/A	Range 13.1-25.7<br>Mean: 19.4%<br>Median: 19.0%
			ACS NSQIP 44143 - Colectomy, partial with end colostomy and closure of distal segment (Hartmann type procedure)[1]	N/A	Range 17.2-28.4<br>Mean: 22.7%<br>Median: 23.0%
			ACS NSQIP 45110 - Proctectomy, complete, combined abdominoperineal, with colostomy[1]	N/A	Range 22.9-33.8<br>Mean: 28.8%<br>Median: 28.9%
Complication rate with TDR (cr.tdr)	24.8% (20% relative reduction)	Molenaar 2023[5]	Adults planned for curative elective colorectal cancer resection.<br>Randomised to physical activity or usual care pre-op.<br>Mean BMI: 27kg/m^2, age: 69 years, 55% male.<br>16% low anterior resection, 93% laparoscopic- or robotic-assisted surgery	128 in the intervention group	17% in the intervention group vs 30% in the usual care group had ≥1 severe complication with CCI>20.<br>13% absolute reduction<br>43% relative reduction
		Daniels 2020	Systematic review and meta-analysis of 19 prehabilitation trials before elective abdominal cancer surgery among older adults	447 in exercise studies	43% in control groups vs 35% in exercise interventions had ≥1 complication<br>8% absolute reduction<br>18% relative reduction<br>21% in control groups vs 13% in exercise interventions has ≥1 pulmonary complication<br>8% absolute reduction<br>38% relative reduction
				605 in multimodal intervention studies	45% in control group vs 34% in multimodal interventions had ≥1 complication<br>11% absolute reduction<br>24% relative reduction

© 2025 Koutoukidis DA et al. *JAMA Network Open.*

Model parameter	Parameter value	Reference	Population description	Sample size	Comparator values
				549 in nutrition studies	54% in control groups vs 35% in nutrition interventions had ≥1 complication<br>19% absolute reduction<br>35% relative reduction
		Sun 2020[3]	Adults planned for bariatric surgery losing weight before surgery<br>Mean BMI: 45kg/m^2, age: 45 years, 20% male.	480,075	Relative reduction in 30-day mortality compared with no weight loss:<br>0-4.9% weight loss: 24%<br>5-9.9% weight loss: 31%<br>≥10% weight loss: 42%
		McKechnie 2024[18]	Adults with obesity undergoing colorectal surgery<br>Mean BMI: 34kg/m^2, age: 67 years, 54% male.	190	69% in control vs 38% in very low energy diet had ≥1 complication<br>31% absolute reduction<br>78% relative reduction<br>51% in control vs 30% in very low energy diet had ≥1 grade 2 complication<br>21% absolute reduction<br>70% relative reduction
		Mahmood 2025[19]	Adults undergoing colorectal resection<br>Systematic review and meta-analysis of multimodal prehabilitation	803	38% in control vs 31% in multimodal prehabilitation had ≥1 complication<br>7% absolute reduction<br>28% relative reduction
Rate of long-term complications (cr.2)	9%	Xu 2023[20]	Adults undergoing colorectal cancer resection<br>Age 71 years, 54% male	16,885	9% had unplanned re-admission between 31 and 90 days
		Wick 2011[21]	Adults undergoing colorectal cancer resection<br>18% with obesity, age: 53 years, 51% male.	10,882	12% had re-admission between 31 and 90 days
		Kim 2024[22]	Adults undergoing laparoscopic or robotic lateral pelvic node dissection after preoperative chemoradiotherapy for locally advanced rectal cancer<br>Age 59 years, 69% male	164	37% developed complications after 90 days and 21% were re-admitted due to these.

© 2025 Koutoukidis DA et al. *JAMA Network Open.*

Model parameter	Parameter value	Reference	Population description	Sample size	Comparator values
			53% low anterior resection, 27% laparoscopic		
		Braga 2005[23]	Adults undergoing laparoscopic or open colectomy for cancer.<br>Age 66 years, 60% male<br>7% with obesity, 49% laparoscopic	391	4-7%: Late hospital readmission occurred in 4% of patients of the laparoscopic group and in 7% of patients of the open group<br>11% (7% laparoscopy, 15% open) reported a long-term complication (between 30 days and 4 years postop)
		Beck 2020[24]	Adults undergoing curative colorectal cancer resection.<br>Age 65 years, 63% male<br>44% anterior resection<br>The patients were followed up for at least 5 years, every 3 months for the first 2 years and every 6 months thereafter, median 9 years	2,158	15% with ≥1 grade 2 complication<br>21.6% with ≥1 complication
		Liu 2010[25]	Adults who underwent curative colorectal cancer resection and survived for >5 years<br>59% male<br>22% with obesity<br>Complications recorded would require healthcare resource use.	669	20% among ostomy cases with a late complication over 11 years and 20% had second surgery >1 year after index surgery<br>9% among anastomosis cases with a late complication over 11 years and 7% had second surgery >1 year after index surgery
		Wan 2014[26]	Patients undergoing colorectal cancer resection<br>Age: 65 years, 50% male.	131,689	20% of those with anastomotic leak had a 30-day re-admission vs 12% of those without a leak.
		NBOCA[7]	England and Wales national average	35,779	10.9% unplanned 30-day readmission after colorectal cancer surgery. Therefore, long-term re-admission is likely to be slightly lower.
Rate of complications resolved among those with a	84%	Kim 2024[22]	Adults undergoing laparoscopic or robotic lateral pelvic node dissection after preoperative chemoradiotherapy for locally advanced rectal cancer<br>Age 59 years, 69% male<br>53% low anterior resection, 27% laparoscopic	164	16% experienced persistent chronic complications at 30-month follow-up.

© 2025 Koutoukidis DA et al. *JAMA Network Open.*

Model parameter	Parameter value	Reference	Population description	Sample size	Comparator values
complication (cresolvedr)					
Mortality rate after complications (cmr)	0.86%	Mualla 2021[27]	Systematic review and meta-analysis of 14 studies on the impact of postoperative complications on survival after curative colorectal cancer resection		Postoperative complications following non-metastatic CRC surgery predicted worse OS rates with hazard ratio of 1.36 à hence that's a 36% higher risk with an absolute of 2.4%, so 2.4*1.36=3.26% → additional absolute risk of 0.86%
Risk of complications re-occurring (rcr)	19%	Borstlap 2017[28]	Patients undergoing low anterior resection for rectal cancer<br>Median follow-up: 3.6 years<br>Age 40% 61-70years, 67% male<br>50% laparoscopic	200	Among 200 patients with anastomotic leak, 38 (19%) had an intervention for anastomotic leakage beyond the 30-day postop.<br>Among patients with anastomotic leak who were alive at 12 months (n=177), 48% had a non-healing anastomotic leak (chronic sinus).
		Petrosyan 2021[29]	Patients undergoing surgery	14,351	49% of those with surgical site infection had a re-admission within 1 year (vs. 19% of those without).
		Wan 2014[26]	Patients undergoing colorectal cancer resection<br>Age: 65 years, 50% male.	131,689	20% of those with anastomotic leak had a 30-day re-admission vs 12% of those without a leak.
		Knechtle 2015[30]	Adults undergoing colectomy<br>58 years, 53% male	390	20% re-admission within 30 days among those with complications in the index admission.
		Gan 2015[31]	Adults undergoing colectomy<br>63 years, ~50% male<br>31% laparoscopic	57,948	Open: 18% 30-day readmission among those with a complication (ileus) in the index admission (vs. 15% without ileus)<br>Laparoscopic: 13% 30-day readmission among those with ileus in the index admission (vs 9% without ileus)
		Wick 2011[32]	Adults undergoing colectomy, 56% colon cancer<br>Age 54 years, 53% male<br>18% laparoscopic, 10% had a surgical site infection	7,020	28% with a complication (surgical site infection) were re-admitted within 90 days (vs. 7% without SSI).

© 2025 Koutoukidis DA et al. *JAMA Network Open.*

Model parameter	Parameter value	Reference	Population description	Sample size	Comparator values
		Lawson 2013[33]	Patients >65 years old who underwent colectomy<br>76 years, 48% male<br>63% with obesity	13,644	28% were re-admitted within 30 days among those with complications in the index admission (vs 8% without).
		D'Souza 2023[34]	Patients undergoing colorectal cancer resection<br>Age 72 years, 55% male	701	23% of those with any complication had an unplanned re-admission at 30-days post index discharge (vs 10% without)
		Ali 2025[35]	Adults undergoing rectal cancer surgery<br>Age 58 years, 37% male<br>35% with obesity, 37% open procedure	403	25% of patients are re-admitted between 30 days and 5 years postop<br>Complications were significantly associated with the risk of re-admission.
		Caminsky 2024[36]	Adults undergoing emergency colectomy<br>67 years, 47% male<br>13% with obesity	141,481	34% were re-admitted within 6 months<br>8% had emergency readmission within 1-6 months for late postcolectomy complication
Cost of complications (cComp)	£6,900 (SE: £1,035)	Daniels 2021[37]	Adults undergoing open right hemicolectomies from 140 English NHS Trusts	9,812	£6,139 was the cost of the average extended surgical ward length of stay due to surgical site infection in 2017, adjusted to £6,900 for 2023 using the NHS Cost inflation index. SE based on 15% of the mean
Utility value of no complications (uSuccess)	0.827 (SD: 0.141)	Downey 2023[10]	Adults undergoing major abdominal surgery<br>Age 64 years, 58% male<br>75% cancer, 73% lower gastrointestinal	19,685	0.827: average utility value at 1 year postop of patients who have had no complications
Utility value of complications (uComp)	0.780 (SD: 0.141)				0.780: average utility value at 1-year postop of patients who have had grade 2-4 complications
Utility value of complications resolved (uResolved)	0.802 (SD: 0.134)				0.802: average utility value at 1-year postop of patients who have had grade 1 complications

© 2025 Koutoukidis DA et al. *JAMA Network Open.*

Model parameter	Parameter value	Reference	Population description	Sample size	Comparator values

[1] ACS values are for a 70-year old patient, varying their body mass index (32 or 40), sex, functional status, diabetes status, hypertension status, ASA status across 64 scenarios.

© 2025 Koutoukidis DA et al. *JAMA Network Open.*

eFigure 9. Cost-effectiveness model of the intervention

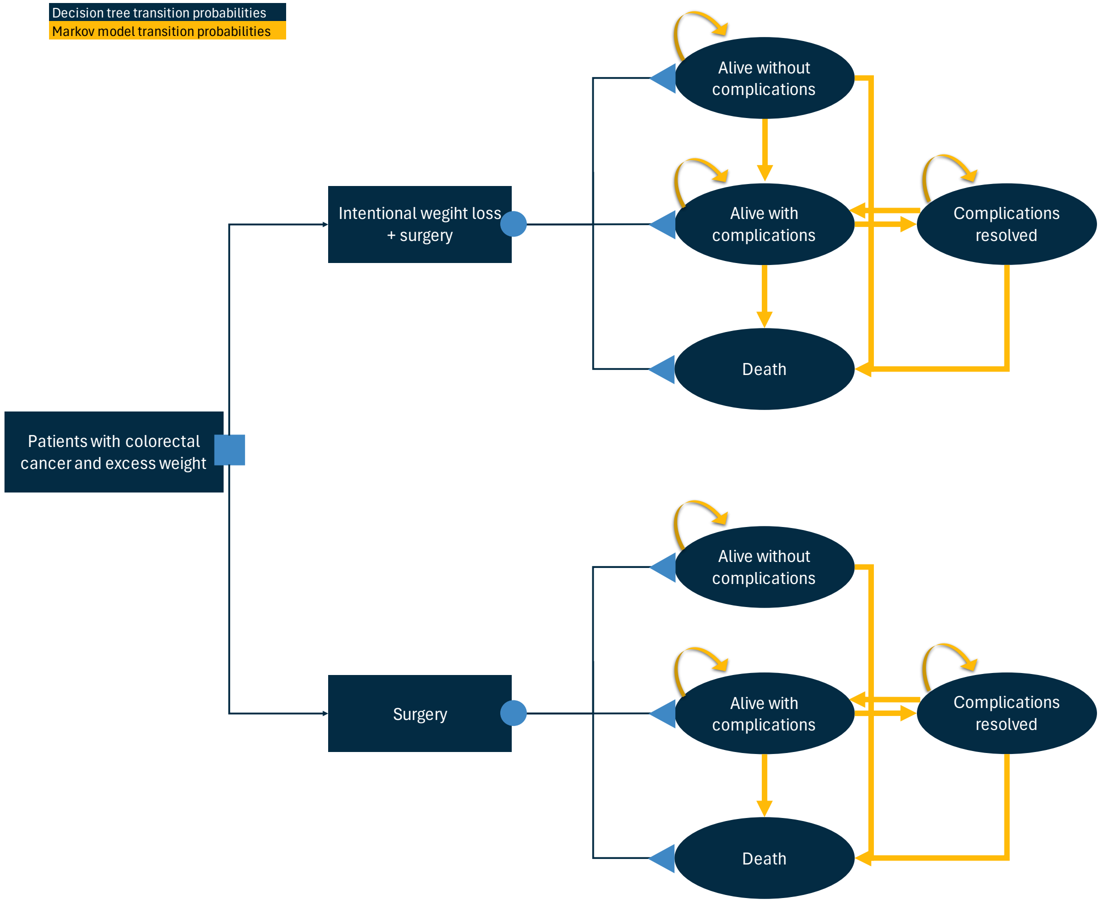

© 2025 Koutoukidis DA et al. *JAMA Network Open.*

eAppendix 4. Recruitment

Out of the 11 hospitals that initially expressed interest, nine were active within six months of receiving ethical approval. The median time for site activation from clinical trial unit approval was 32 days (IQR: 3-44 days). Eight sites recruited at least one participant to the study. Between 28th March 2023 and 13th August 2024 (16.3 months), 414 participants meeting the BMI criteria were assessed for eligibility. Of those, 248 (60%) were ineligible with the main reasons being insufficient time prior to surgery (46%) and significant comorbidity (21%). Of 166 potentially eligible participants, 150 (90%) were approached, of whom 75 (50%) agreed to participate in the study. The most common reasons for declining participation were inability to manage additional requirements before surgery (37%) and unwillingness to eat meal replacement products instead of usual food (28%). Decliners did not differ significantly in terms of sex (40% vs 39%) but were older [68.8 (9.7) vs 64.1 (8.7), p<0.01] and had a lower BMI [(33.4 (5.5) vs 35.4 (5.4), p=0.03] to those enrolled. Seventy-five participants were randomised and we aimed to replace participants who were ineligible after randomisation (n=2) or declined surgery (n=2); however we only replaced three of them as the fourth participant declined surgery after recruitment had been closed.

eFigure 10. Planned vs actual recruitment rate (excluding participants who were withdrawn and replaced) (1 site closed early due to lack of recruitment)

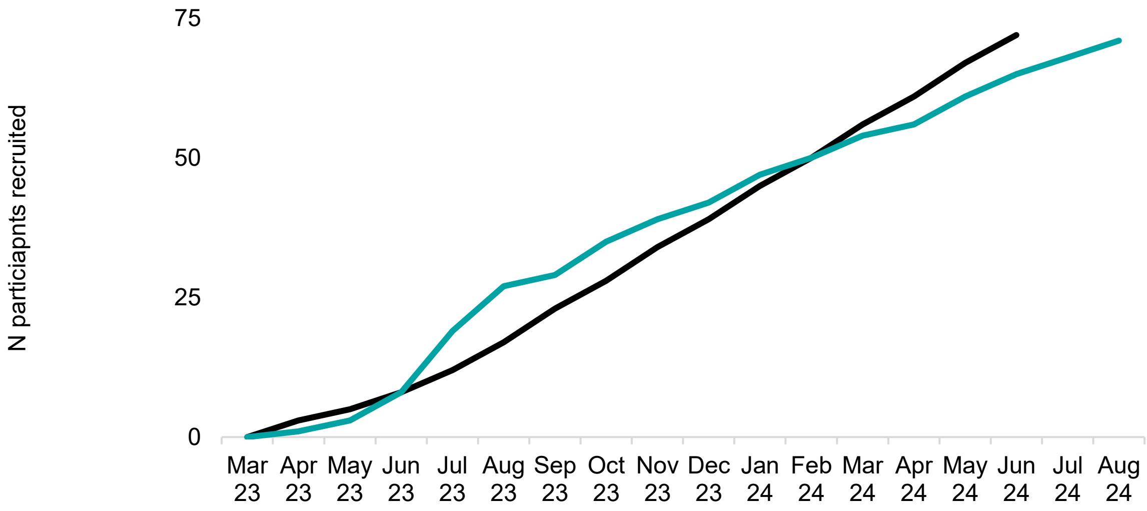

	Mar 23	Apr 23	May 23	Jun 23	Jul 23	Aug 23	Sep 23	Oct 23	Nov 23	Dec 23	Jan 24	Feb 24	Mar 24	Apr 24	May 24	Jun 24	Jul 24	Aug 24
Minimum total cumulative	0	3	5	8	12	17	23	28	34	39	45	50	56	61	67	72		
Actual total cumulative	0	1	3	8	19	27	29	35	39	42	47	50	54	56	61	65	68	71
Sites active	3	5	8	9	9	9	9	9	9	9	9	9	8	8	8	8	8	8

Minimum total cumulative   Actual total cumulative

© 2025 Koutoukidis DA et al. *JAMA Network Open.*

eTable 16. Demographic and operative characteristics

	Intervention (n=36)	Usual care (n=35)
Age, years	65.1 (6.8)	63.0 (10.4)
Sex		
Female	11 (31%)	17 (49%)
Male	25 (69%)	18 (51%)
BMI, kg/m^2	35.5 (5.5)	35.2 (5.3)
Ethnicity		
White	34 (94%)	34 (97%)
Asian	-	1 (3%)
Black	1 (3%)	-
Other	1 (3%)	-
Index of multiple deprivation		
1-3 (most deprived)	6 (17%)	5 (14%)
4-7	15 (42%)	16 (46%)
8-10 (least deprived)	15 (42%)	14 (40%)
On medication for type 2 diabetes	7 (19%)	7 (20%)
On medication for hypertension	18 (51%)	15 (43%)
WHO (ECOG) performance status		
0	26 (72%)	24 (69%)
1	10 (28%)	10 (29%)
2	0 (0%)	1 (3%)
ASA grade		
I	4 (11%)	5 (14%)
II	21 (58%)	20 (57%)
III	11 (31%)	10 (29%)
Time between randomisation and surgery, days		
Mean (SD)	37.4 (17.1)	40.4 (25.5)
Median (IQR)	32.5 (18.8)	34.0 (17.0)
Time between admission and discharge, days		
Mean (SD)	6.9 (6.4)	6.1 (3.7)
Median (IQR)	4.5 (3.5)	5 (3)
Initial operation approach		
Laparoscopic (incl. n = 4)	21 (58%)	22 (63%)
Laparoscopic (converted to open)	1 (3%)	2 (6%)
Open	4 (11%)	2 (6%)
Robotic	10 (28%)	9 (26%)
Operation type		
Right hemicolectomy	7 (19%)	9 (26%)
Extended right hemicolectomy	0 (0%)	2 (6%)
Left hemicolectomy	2 (6%)	3 (9%)
Anterior Resection	19 (53%)	18 (51%)
APER	6 (17%)	1 (3%)
Hartmann’s procedure	1 (3%)	2 (6%)
Pelvic exenteration	1 (3%)	0 (0%)

Data are presented as means ± SD or n (%) as appropriate, unless otherwise indicated.
APER: Abdominoperineal excision of the rectum, ASA: American Society of Anaesthesiologists, BMI: Body mass index, WHO: World Health Organisation

© 2025 Koutoukidis DA et al. *JAMA Network Open.*

eTable 17. Staging and clinical characteristics

		Intervention	Usual care
**Diagnostic staging**			
T stage	Tx	5 (14%)	4 (11%)
	T0	-	1 (3%)
	T1	1 (3%)	3 (9%)
	T2	13 (36%)	11 (31%)
	T3	15 (42%)	15 (43%)
	T4	2 (6%)	1 (3%)
N stage	Nx	-	3 (9%)
	N0	23 (64%)	19 (54%)
	N1	11 (31%)	12 (34%)
	N2	2 (6%)	1 (3%)
M stage	M0	36 (100%)	35 (100%)
**Final pathology staging**			
T stage	T0	-	-
	T1	5 (14%)	5 (14%)
	T2	7 (19%)	9 (26%)
	T3	16 (44%)	11 (31%)
	T4	5 (14%)	7 (20%)
	No cancer	3 (8%)	3 (9%)
N stage	Nx	-	-
	N0	16 (44%)	22 (63%)
	N1	12 (33%)	6 (17%)
	N2	5 (14%)	4 (11%)
	No cancer	3 (8%)	3 (9%)
M stage	Mx	2 (6%)	-
M stage	M0	30 (83%)	32 (91%)
	M1	1 (3%)	-
	No cancer	3 (8%)	3 (9%)
Days between diagnosis and first discussion at MDT			
	Mean (SD)	8.4 (9.3)	7.7 (7.0)
	Median (IQR)	6 (9.5)	7 (13)
Days between first discussion at MDT and randomisation			
	Mean (SD)	62.2 (83.2)	33.3 (42.7)
	Median (IQR)	17 (45.0)	19 (24.0)
Number of nodes examined			
	Mean (SD)	24.8 (11.5)	23.9 (8.4)
	Median (IQR)	22.5 (13)	23 (13.5)
Nodes positive			
	N (%) of patients with positive nodes	16 (44%)	10 (29%)

© 2025 Koutoukidis DA et al. *JAMA Network Open.*

	Intervention	Usual care
Mean (SD) of positive	1.5 (3.1)	1.2 (3.0)
Median (IQR) of positive	0 (1.3)	0 (1)
Lesion size (mm)		
Mean (SD)	35.4 (14.3)	35.1 (17.3)
Median (IQR)	35 (21)	33 (15)
Missing	1 (3%)	1 (3%)
Tumour type		
Adenocarcinoma	28 (85%)	31 (97%)
Mucinous	4 (12%)	1 (3%)
Signet ring	1 (3%)	-
No cancer	3 (8%)	3 (9%)
Differentiation by worst area		
GX	2 (6%)	2 (6%)
G1	1 (3%)	5 (14%)
G2	26 (72%)	21 (60%)
G3	4 (11%)	4 (11%)
Cancer vascular or lymphatic invasion		
No	14 (42%)	23 (72%)
Yes	19 (58%)	9 (28%)
Not applicable	3	3
Neo-adjuvant treatment [1]		
None	29 (81%)	32 (91%)
Chemotherapy only	-	1 (3%)
Short-course radiotherapy	2 (6%)	-
Long-course chemo/radio	5 (14%)	2 (6%)
Other	2 (6%)	-
Adjuvant treatment[1]		
None	20 (56%)	24 (69%)
Chemotherapy	16 (44%)	11 (31%)
Radiotherapy	-	1 (3%)
Stoma/anastomosis formed[2]		
Extracorporeal anastomosis	9 (25%)	9 (26%)
Intracorporeal anastomosis	18 (50%)	20 (57%)
Loop ileostomy	4 (11%)	3 (9%)
End colostomy	7 (19%)	5 (14%)
Discharge destination		
Home	35 (97%)	35 (100%)
Rehabilitation centre	1 (3%)	-

[1]Some participants received multiple types of treatment. [2]Some participants had more than one procedure

© 2025 Koutoukidis DA et al. *JAMA Network Open.*

eAppendix 5. Reasons for intervention discontinuation

Of the 4 participants discontinuing the intervention, one discontinued it after 20 days due to palatability (n=1) and two were due to adverse events: one discontinued it after 17 days due to hunger leading to low adherence and one after 17 days due to tremor and hunger but with no evidence of hypoglycaemia on self-monitoring. Another one discontinued the intervention after 44 days due to constipation, abdominal pain, and anxiety due to the complex and long pre-operative pathway. Three of the four participants had long pre-operative pathways, as at the time of discontinuation they had another 23, 39, 8, and 65 days until their surgery, respectively.

eAppendix 6. Usual care group changes in relevant diet and physical activity behaviours

Most participants (82%) reported changing their diet and or activity in some way before surgery: 64% of participants reported reducing their portion sizes, 45% made other changes to their diet to lose weight, and 36% reported increasing their physical activity. Only 6% (n=2) reported starting consuming meal replacement products to lose weight before surgery. However, nobody attended a formal weight loss programme and 18% reported no specific efforts to lose weight.

eAppendix 7. Intervention evaluation

Sixty-seven percent of the intervention participants liked or strongly liked the intervention. 89% considered that it was fair they were asked to follow it. 89% felt confident/very confident following it. 78% found it acceptable. Only 22% felt that the intervention interfered with their other priorities. Almost everyone (97%) reported that it was clear to them how the intervention helped them to lose weight. 78% reported that the intervention helped them feel better prepared for surgery. The intervention met or exceeded the expectations of 79% of the intervention participants providing feedback and 76% of them gave it a 4-star or 5-star rating (n=2 with missing data) (eFigure 4).

eFigure 11. Intervention evaluation

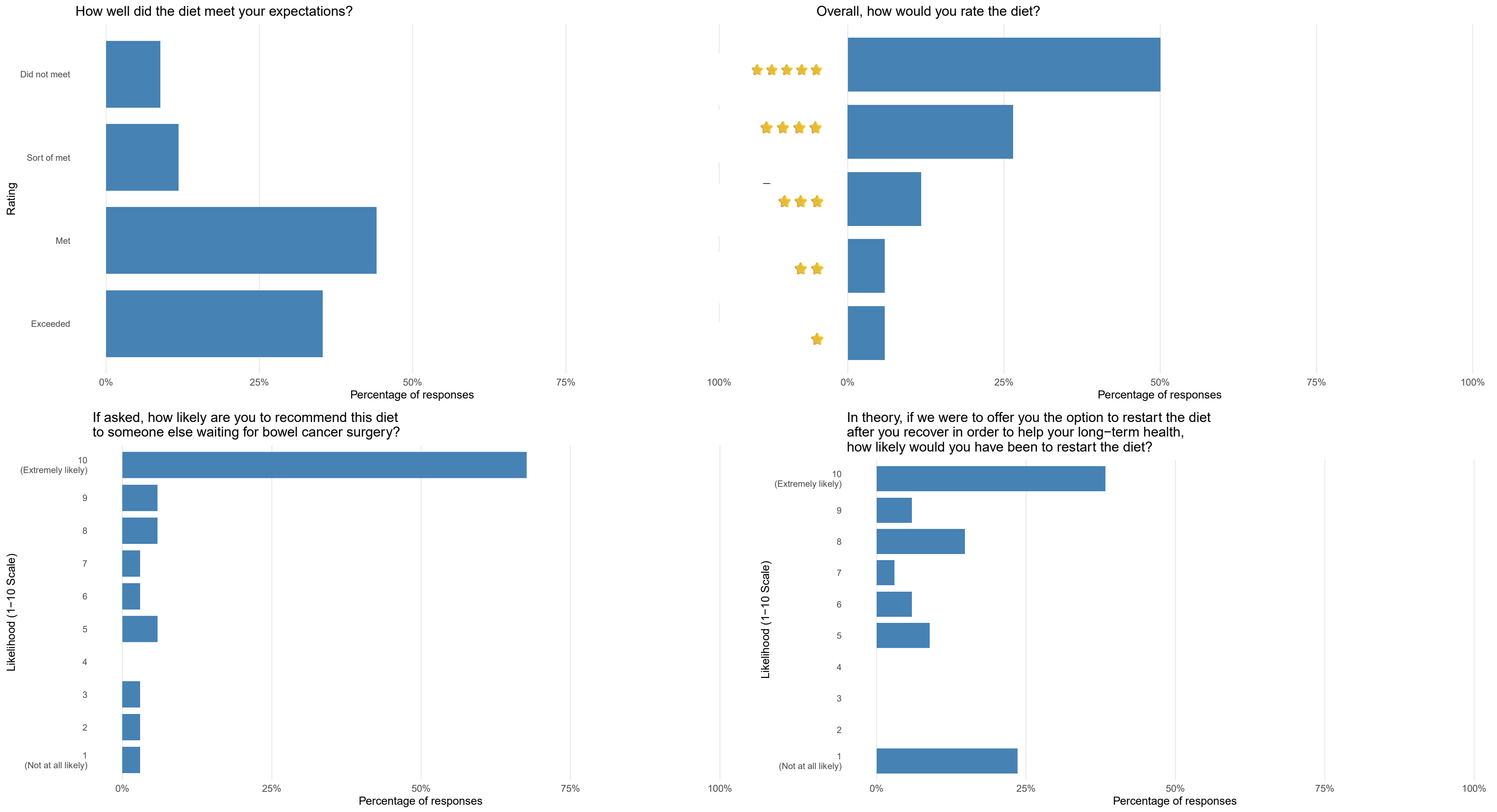

© 2025 Koutoukidis DA et al. *JAMA Network Open.*

eTable 18. Oncological and operative outcomes

	Intervention (n=36)	Usual care (n=35)	Adjusted Mean Difference or Odds Ratio (95% CI)
Resection margins, mm[1]	34.2 (29.2)	39.1 (41.3)	-2.17 (-25.4, 21.1)
Operative time, minutes[2]	299.6 (132.3)	247.7 (93.3)	8.4 (-36.2, 53.1)
Length of initial hospital stay, days	6.9 (6.4)	6.1 (3.7)	0.79 (-1.76 3.33)
N of days alive and out of hospital within 30 days postoperatively	22.9 (5.9)	23.7 (3.8)	-0.95 (-3.33 1.43)
Intraoperative blood loss	1 (3%)	1 (3%)	1.11 (0.06 20.46)
Conversion to open surgery	1 (3%)	2 (6%)	0.44 (0.04 5.28)
Reoperation during index hospital stay	0 (0%)	2 (6%)	-
30-day readmission	3 (8%)	1 (3%)	3.18 (0.31 33.04)
Reoperation between initial discharge and 30 days postoperatively	2 (6%)	0 (0%)	-

Data are presented as means ± SD or n (%) as appropriate, unless otherwise indicated. AMDs calculated using linear regression. Odds ratios calculated using logistic regression.

[1]Reported for n=19 and n=21 in intervention and usual care, respectively.

[2]Mean difference also adjusted for categories of operation type as per eTable 2.

© 2025 Koutoukidis DA et al. *JAMA Network Open.*

eTable 19. Complications by type (intention-to-treat)

	Patients with complications N (%)		OR (95% CIs)
	Intervention (n=36)	Care as usual (n=35)	
Cardiac	-	2 (6%)	-
Arrythmia	-	2 (6%)	-
Respiratory	-	1 (3%)	-
Pleural effusion	-	1 (3%)	-
Neurological	1 (3%)	2 (6%)	0.44 (0.04 5.28)
Postoperative pain	-	2 (6%)	-
Altered sensation	-	1 (3%)	-
Vasovagal episode	1 (3%)	-	-
Gastrointestinal	5 (14%)	7 (20%)	0.63 (0.18 2.23)
Postoperative ileus	3 (8%)	2 (6%)	1.51 (0.23 10.19)
Radiologically defined anastomotic leak	1 (3%)	1 (3%)	0.90 (0.05 15.47)
Intestinal fistula	1 (3%)	-	-
Bowel frequency change from pre-surgery	1 (3%)	1 (3%)	0.90 (0.05 15.47)
Diarrhoea	1 (3%)	2 (6%)	0.49 (0.04 5.85)
Other	1 (3%)	2 (6%)	0.44 (0.04 5.28)
Renal/Genitourinary	1 (3%)	2 (6%)	0.44 (0.03 5.94)
Urinary function - Alteration in pre-operative function, urinary retention, Incontinence, UTI	1 (3%)	2 (6%)	0.44 (0.03 5.94)
Wound	5 (14%)	6 (17%)	0.76 (0.20 2.92)
Surgical site infection (SSI)	4 (11%)	3 (9%)	1.36 (0.28 6.69)
Non-infectious wound discharge	1 (3%)	1 (3%)	0.95 (0.05 16.61)
Wound dehiscence	-	2 (6%)	-

© 2025 Koutoukidis DA et al. *JAMA Network Open.*

eTable 20. Weight, body composition, physical function, and quality of life

	Supported weight loss						Care as usual						**Between-groups adjusted mean difference (95% CI) from baseline to admission**	**Between-groups adjusted mean difference (95% CI) from baseline to 30 days postoperatively**
	N	Baseline mean (SD)	N	Change on admission	N	Change from baseline at 30 days postoperatively	N	Baseline mean (SD)	N	Change on admission	N	Change from baseline at 30 days postoperatively		
Weight, kg	36	104.4 (17.0)	36	-6.1 (3.0)	34	-8.1 (3.8)	35	101.1 (17.5)	35	-1.9 (1.9)	33	-3.7 (3.8)	-4.3 (-5.8, -2.7)	-4.3 (-5.9, -2.7)
Fat-free mass, kg	35	63.9 (13.6)	27	-1.6 (7.0)	31	-3.0 (10.9)	34	61.1 (14.0)	30	-0.4 (7.0)	31	-1.7 (7.3)	0.1 (-3.9, 4.0)	-1.3 (-5.1, 2.6)
Fat-free mass, %	35	61.6 (9.1)	27	2.8 (6.5)	31	3.1 (8.5)	34	60.7 (9.7)	30	1.1 (7.1)	31	0.8 (6.9)	2.3 (-1.1, 5.8)	2.0 (-1.4, 5.4)
Sit to stand time, secs	35	13.2 (6.1)			26	-0.7 (3.2)	35	12.0 (4.8)			31	1.0 (6.5)		-1.5 (-4.4, 1.4)
EQ-5D-5L index (0-1)	36	0.86 (0.16)	36	0.02 (0.13)	36	-0.04 (0.18)	35	0.88 (0.13)	34	0.01 (0.13)	35	-0.04 (0.18)	0.01 (-0.06, 0.09)	0.00 (-0.07 0.07)
EQ-5D-5L VAS (0-100)	36	71.2 (17.7)	35	4.8 (20.4)	35	1.5 (17.8)	35	72.6 (17.5)	30	-5.0 (16.6)	32	-0.2 (13.2)	5.1 (-2.9, 13.2)	0.9 (-7.1, 8.8)
HADS anxiety (0-21)	36	4.4 (3.4)	34	0.5 (2.4)	35	-0.5 (3.3)	35	5.5 (3.8)	30	-0.1 (3.4)	32	-1.3 (3.9)	0.2 (-1.4, 1.8)	0.9 (-0.7, 2.4)
HADS depression (0-21)	36	3.3 (3.5)	34	0.3 (2.0)	35	0.0 (3.5)	35	3.4 (3.2)	30	0.1 (2.6)	32	0.8 (3.9)	0.1 (-1.5, 1.6)	-0.7 (-2.2, 0.8)

© 2025 Koutoukidis DA et al. *JAMA Network Open.*

eTable 21. Sensitivity analysis of the number and proportion of participants with minimally clinically important increase and reduction in anxiety and depression (by ≥1.7 points) between baseline and admission by group

	Intervention, n (%) (n=36)	Care as usual, n (%) (n=35)	OR (95% CI)
Anxiety increased since baseline by ≥ 1.7 points	12 (33%)	9 (26%)	1.62 (0.54 4.85)
Anxiety reduced since baseline by ≥ 1.7 points	7 (19%)	7 (20%)	0.92 (0.28 3.05)
Depression increased since baseline by ≥ 1.7 points	7 (19%)	6 (17%)	1.30 (0.36 4.73)
Depression reduced since baseline by ≥ 1.7 points	4 (11%)	6 (17%)	0.61 (0.15 2.52)

* Note: the denominator of the percentages may include patients with missing data.

© 2025 Koutoukidis DA et al. *JAMA Network Open.*

eAppendix 8. Adverse events

Assessment

During study visits and, for intervention participants, at their weekly phone calls with the dietitian, participants were prompted to report if they had experienced adverse events. Additional adverse events were extracted from medical records. Adverse events were rated by participants as mild, moderate, or severe and also coded using the CTCAE system.

Serious adverse events

Excluding postoperative complications, there were 2 serious adverse events (1 anaphylaxis at anaesthesia pre-surgery in the usual care group and 1 admission for optimisation of bronchiectasis pre-surgery in the intervention group that was deemed unrelated to the intervention).

Non-serious adverse events

There were 2 non-serious AEs reported in the control group and 47 in the intervention group; all of which were classed by participants as either mild (n=37) or moderate (n=12). Between randomisation and surgery, there were n=22 and n=1 participants in the intervention and usual care groups, respectively, with at least 1 AE. Up to 30-days postoperatively, the respective numbers were n=1 and n=2. The most common AEs in the intervention group preoperatively were constipation (n=7 mild and n=3 moderate) and dizziness (n=8 mild and n=1 moderate) with all other AEs reported by ≤2 participants.

© 2025 Koutoukidis DA et al. *JAMA Network Open.*

eFigure 12. Complications by group based on the per protocol analysis

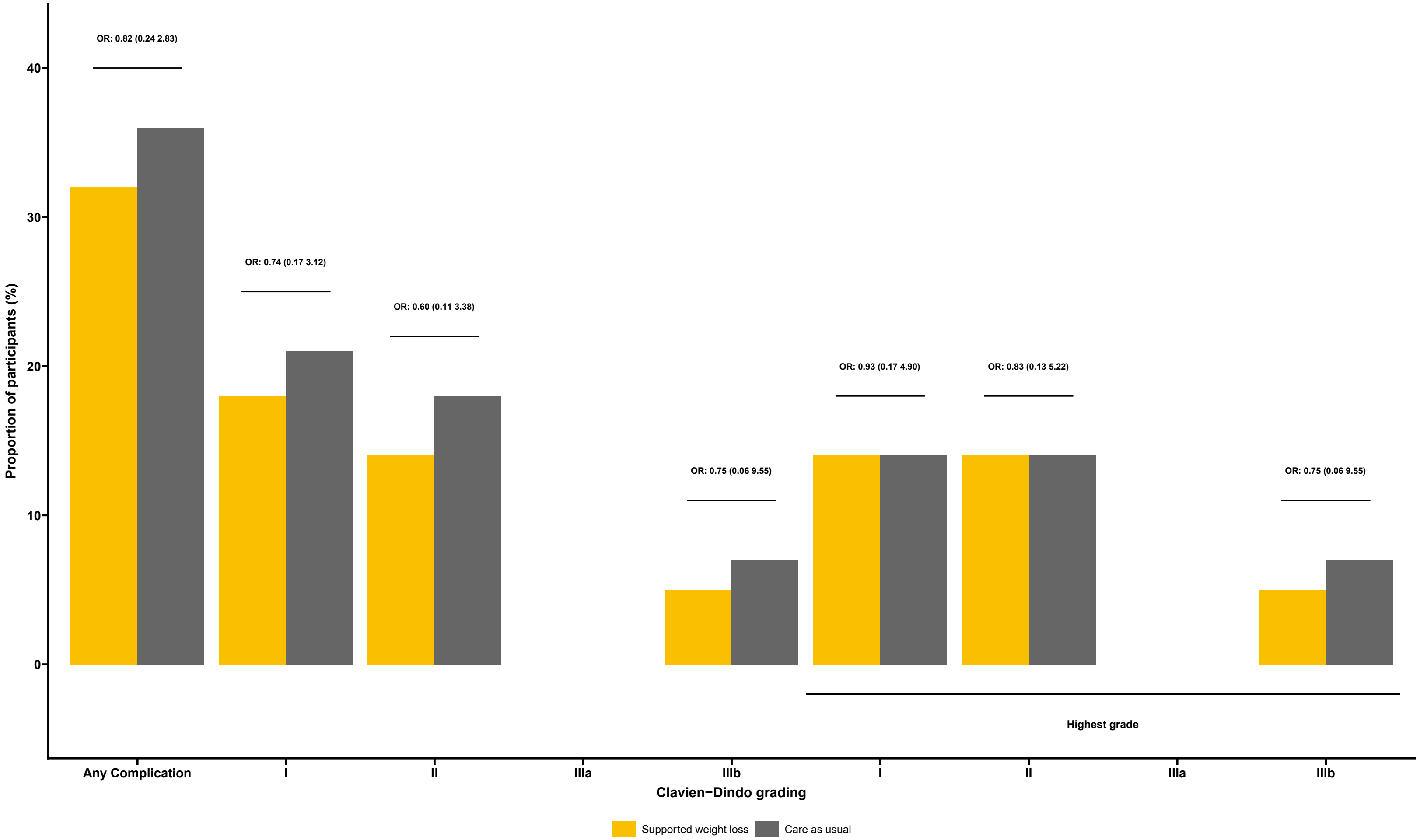

© 2025 Koutoukidis DA et al. *JAMA Network Open.*

eTable 22. Complications by type and group based in the per-protocol analysis

Complications by type	Patients with complications, n (%)		OR (95% CI)
	Intervention (n=22)	Care as usual (n=28)	
Any complication (at discharge or 30 days postoperatively)	7 (32%)	10 (36%)	0.82 (0.24 2.83)
I	4 (18%)	6 (21%)	0.74 (0.17 3.12)
II	3 (14%)	5 (18%)	0.60 (0.11 3.38)
IIIa	-	-	-
IIIb	1 (5%)	2 (7%)	0.75 (0.06 9.55)
Highest grade of complication reported			
I	3 (14%)	4 (14%)	0.93 (0.17 4.90)
II	3 (14%)	4 (14%)	0.83 (0.13 5.22)
IIIa	-	-	-
IIIb	1 (5%)	2 (7%)	0.75 (0.06 9.55)
Cardiac	-	1 (4%)	-
Arrythmia	-	1 (4%)	-
Respiratory	-	1 (4%)	-
Pleural effusion	-	1 (4%)	-
Neurological	1 (5%)	2 (7%)	0.51 (0.04 6.43)
Postoperative pain	-	2 (7%)	-
Altered sensation	-	1 (4%)	-
Vasovagal episode	1 (5%)	-	-
Gastrointestinal	3 (14%)	5 (18%)	0.68 (0.14 3.32)
Postoperative ileus	1 (5%)	2 (7%)	0.46 (0.03 6.41)
Diarrhoea	1 (5%)	2 (7%)	0.70 (0.05 9.14)
Radiologically defined anastomotic leak	1 (5%)	-	-
Other	1 (5%)	2 (7%)	0.51 (0.04 6.43)
Renal/Genitourinary	1 (5%)	1 (4%)	1.00 (0.05 20.07)
Urinary function - Alteration in pre-operative function, urinary retention, Incontinence, UTI	1 (5%)	1 (4%)	1.00 (0.05 20.07)
Wound	1 (5%)	4 (14%)	0.13 (0.01 2.14)
Surgical site infection (SSI)	1 (5%)	2 (7%)	0.43 (0.03 6.71)
Wound dehiscence	-	2 (7%)	-

© 2025 Koutoukidis DA et al. *JAMA Network Open.*

eTable 23. Per-protocol analysis of anthropometric and quality of life outcomes

	Supported weight loss						Care as usual						Between-groups adjusted mean difference (95% CI) from baseline to admission	Between-groups adjusted mean difference (95% CI) from baseline to 30 days postoperatively
	N	Baseline mean (SD)	N	Change on admission	N	Change from baseline at 30 days postoperatively	N	Baseline mean (SD)	N	Change on admission*	N	Change from baseline at 30 days postoperatively		
Weight, kg	22	103.0 (13.0)	22	-7.8 (2.2)	22	-9.1 (3.7)	28	101.7 (17.3)	28	-1.4 (1.7)	26	-4.0 (3.6)	-6.4 (-8.1 -4.7)	-5.0 (-6.7 -3.3)
Fat-free mass, kg	22	64.1 (12.7)	17	-1.6 (4.7)	21	-2.9 (4.0)	27	61.5 (13.4)	23	0.1 (7.9)	24	-2.0 (8.1)	-0.4 (-4.2 3.4)	-0.9 (-4.5 2.7)
Fat-free mass, %	22	62.0 (7.8)	17	3.7 (5.9)	21	3.4 (4.1)	27	60.9 (10.2)	23	1.1 (7.8)	24	0.6 (7.7)	3.3 (-0.4 7.0)	2.6 (-0.9 6.1)
Sit to stand time, secs	22	12.2 (3.7)			17	0.1 (1.8)	28	12.4 (5.3)			24	1.3 (7.3)		-1.3 (-5.3, 2.6)
EQ-5D-5L index (0-1)	22	0.89 (0.15)	22	-0.00 (0.09)	22	-0.03 (0.13)	28	0.88 (0.12)	27	0.02 (0.12)	28	-0.03 (0.15)	-0.02 (-0.10 0.05)	-0.00 (-0.07 0.07)
EQ-5D-5L VAS (0-100)	22	73.6 (16.0)	22	1.3 (19.7)	22	5.0 (16.5)	28	74.5 (16.4)	23	-6.4 (14.3)	26	0.5 (12.8)	6.8 (-2.1 15.6)	4.8 (-3.8 13.5)
HADS anxiety (0-21)	22	4.0 (2.8)	21	0.3 (2.3)	22	-1.3 (2.7)	28	5.1 (4.0)	23	0.0 (3.4)	26	-1.1 (4.0)	-0.1 (-1.9 1.7)	-0.3 (-2.0 1.5)
HADS depression (0-21)	22	2.6 (3.4)	21	0.6 (1.9)	22	-0.8 (3.4)	28	3.1 (3.0)	23	0.0 (2.2)	26	0.8 (4.2)	0.5 (-1.3 2.2)	-1.6 (-3.3 0.2)

© 2025 Koutoukidis DA et al. *JAMA Network Open.*

eFigure 13. Interaction of quality of life (EQ-5D visual analogue scale), timepoint, and age

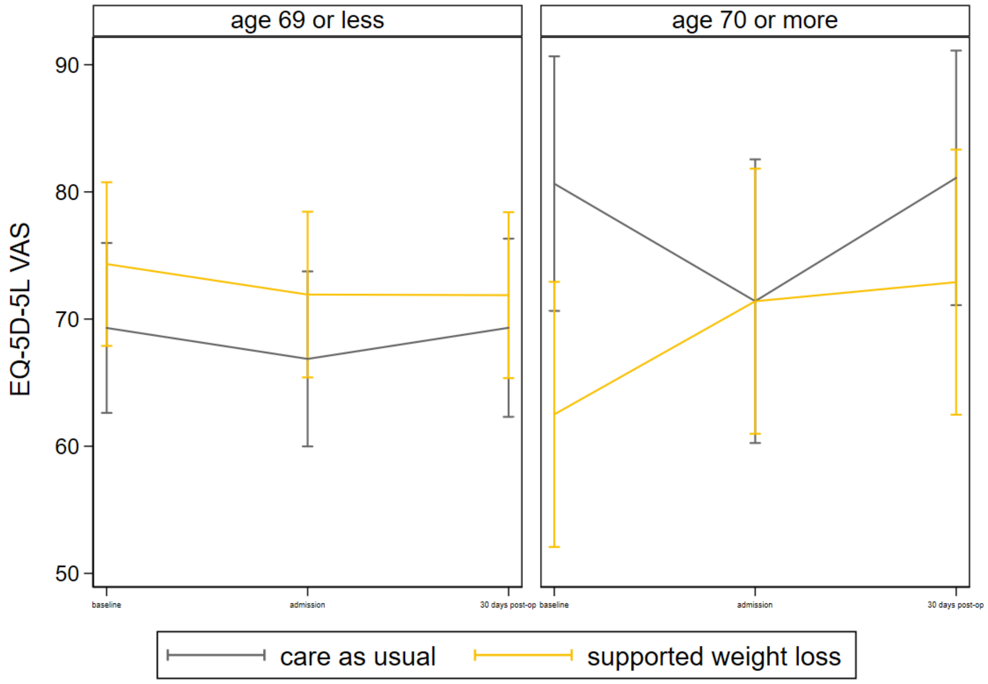

eTable 24. P-values of interaction terms from sensitivity analysis stratified by age and performance status

	P-value of interaction term with age	P-value of interaction term with performance status
Any complications	0.407	0.401
Any CD Grade I complications	0.689	0.739
Any CD Grade II complications	0.035	0.114
Length of stay	0.921	0.811
Operative time	0.846	0.326
Sit to stand time, secs	0.960	0.663
Weight (kg) *	0.501	0.508
Fat-free mass, kg*	0.536	0.097
Fat-free mass, %*	0.449	0.069
EQ-5D-5L index (0-1)*	0.199	0.958
EQ-5D-5L VAS (0-100)*	0.042	0.140
HADs anxiety (0-21)*	0.446	0.245
HADs depression (0-21)*	0.966	0.355

Other grades of complications are not shown due to insufficient data. *These variables vary over time so “timepoint” is included in the interaction term.

© 2025 Koutoukidis DA et al. *JAMA Network Open.*

eTable 25. Subgroup analysis by colon (n=55) or rectum (n=16) in the intention-to-treat population

		Intervention	Care as usual	Adjusted1 difference or odds ratio (95% CI)
Any complication	Colon (n=55)	11 (41%)	12 (43%)	0.89 (0.30, 2.67)
	Rectum (n=16)	3 (33%)	2 (29%)	N/A[2]
Grade 1 complications	Colon (n=55)	7 (26%)	7 (25%)	0.94 (0.27, 3.24)
	Rectum (n=16)	0 (0%)	1 (14%)	N/A[2]
Grade 2 complications	Colon (n=55)	5 (19%)	8 (29%)	0.50 (0.14, 1.85)
	Rectum (n=16)	3 (33%)	1 (14%)	N/A[2]
Operative time, mins	Colon (n=55)	266.0 (116.6)	239.6 (79.0)	5.9 (-42.8, 54.6)[3]
	Rectum (n=16)	400.2 (131.0)	280.0 (140.2)	16.8 (-170.9, 204.4)[3]
Length of hospital stay, days	Colon (n=55)	5.96 (6.79)	6.25 (4.10)	-0.30 (-3.46, 2.85)
	Rectum (n=16)	9.67 (4.50)	5.71 (1.70)	3.28 (-1.30, 7.87)

[s1]Adjusted for minimisation factors [2]Model did not converge likely due to small sample. [3]Also adjusted for type of operation.

© 2025 Koutoukidis DA et al. *JAMA Network Open.*

eFigure 14. Incremental cost-effectiveness plane

The red line indicates the £20,000 threshold.

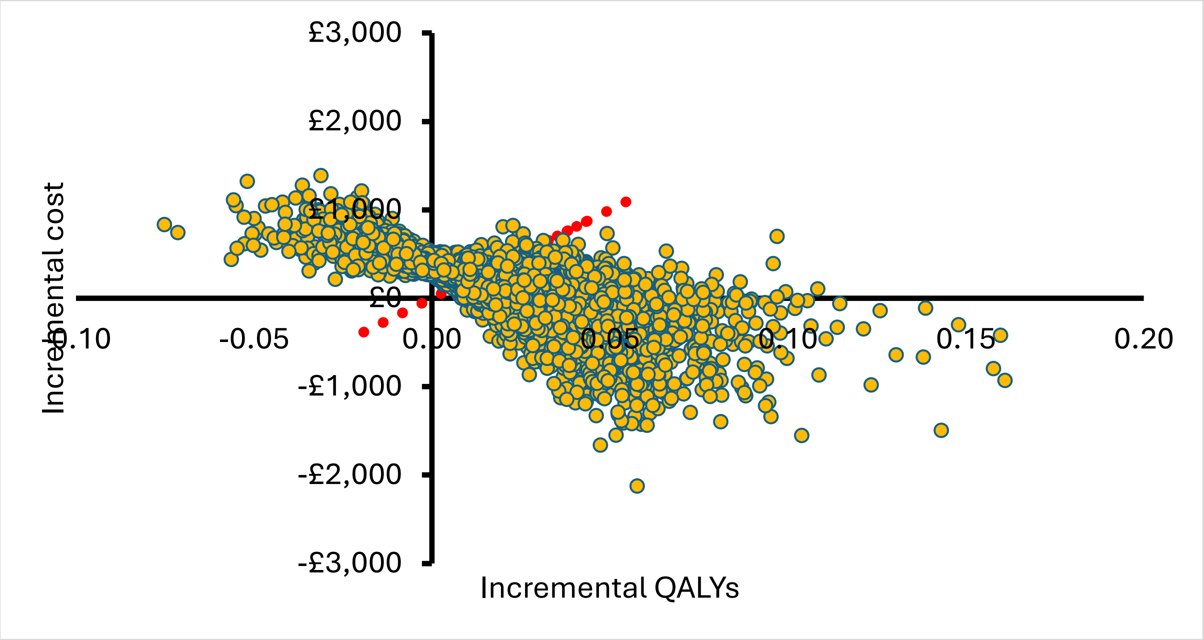

eFigure 15. Cost-effectiveness acceptability curve for the reference case analysis

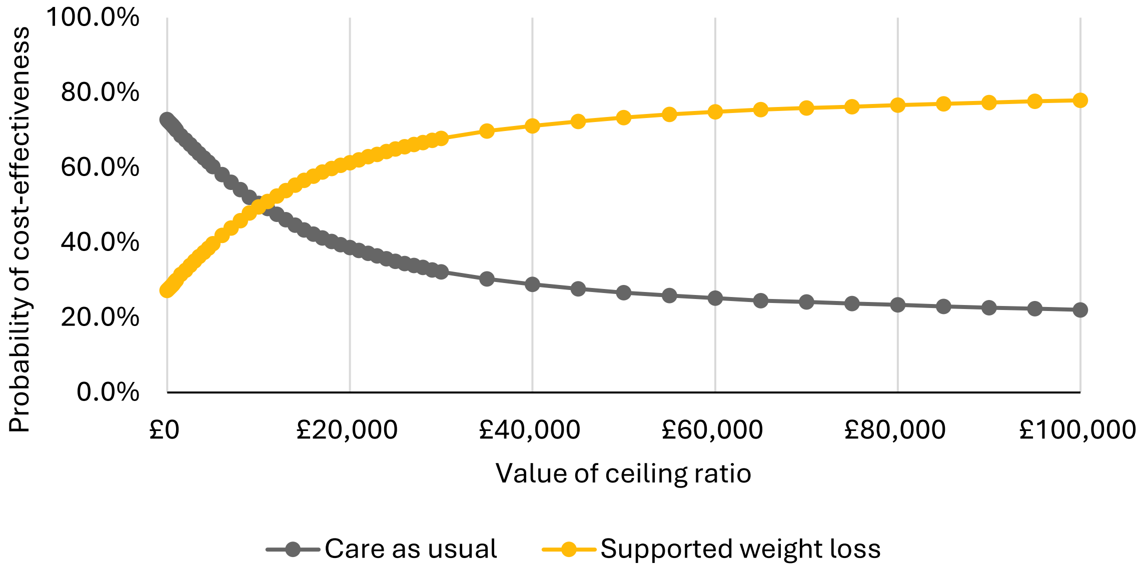

© 2025 Koutoukidis DA et al. *JAMA Network Open.*

eTable 26. Incremental cost-effectiveness ratio (ICER) based on the reference case and sensitivity analyses

	Deterministic ICER	Probabilistic ICER
Reference case	£7,623	£7,238
60y male	£1,665	£1,161
70y male	£9,216	£8,564
80y male	£34,978	£34,314
60y female	£202	(£172)
70y female	£5,696	£5,090
80y female	£24,884	£24,692
15% relative reduction in complications	£14,371	£13,675
25% relative reduction in complications	£3,574	£3,670
Based on expected costs	£10,592	£10,216
Utility value returning to baseline	£8,066	£8,320
RCR 14% instead of 19%	£14,390	£13,425

© 2025 Koutoukidis DA et al. *JAMA Network Open.*

eReferences

1. Lemay KR, Tulloch HE, Pipe AL, Reed JL. Establishing the Minimal Clinically Important Difference for the Hospital Anxiety and Depression Scale in Patients With Cardiovascular Disease. *J Cardiopulm Rehabil Prev*. Nov 2019;39(6):E6-E11. doi:10.1097/HCR.0000000000000379
2. Slankamenac K, Nederlof N, Pessaux P, et al. The comprehensive complication index: a novel and more sensitive endpoint for assessing outcome and reducing sample size in randomized controlled trials. *Annals of surgery*. Nov 2014;260(5):757-62; discussion 762-3. doi:10.1097/SLA.0000000000000948
3. Sun Y, Liu B, Smith JK, et al. Association of Preoperative Body Weight and Weight Loss With Risk of Death After Bariatric Surgery. *JAMA Netw Open*. May 1 2020;3(5):e204803. doi:10.1001/jamanetworkopen.2020.4803
4. Daniels SL, Lee MJ, George J, et al. Prehabilitation in elective abdominal cancer surgery in older patients: systematic review and meta-analysis. *BJS Open*. Sep 22 2020;4(6):1022-41. doi:10.1002/bjs5.50347
5. Molenaar CJL, Minnella EM, Coca-Martinez M, et al. Effect of Multimodal Prehabilitation on Reducing Postoperative Complications and Enhancing Functional Capacity Following Colorectal Cancer Surgery: The PREHAB Randomized Clinical Trial. *JAMA Surg*. Jun 1 2023;158(6):572-581. doi:10.1001/jamasurg.2023.0198
6. NICE. NICE health technology evaluations: the manual. National Institute for Health and Care Excellence. Accessed 07 April 2025, https://www.nice.org.uk/process/pmg36/resources/nice-health-technology-evaluations-the-manual-pdf-72286779244741
7. NBOCA. State of the Nation Report. Accessed 07 April 2025, https://www.nboca.org.uk/wp-content/uploads/2024/02/NBOCA-SotN.pdf
8. Afifi AM, Elmehrath AO, Ruhban IA, et al. Causes of Death Following Nonmetastatic Colorectal Cancer Diagnosis in the U.S.: A Population-Based Analysis. *The oncologist*. Sep 2021;26(9):733-739. doi:10.1002/onco.13854
9. Daniels I, Tuson R, Hargreaves J. What are the financial implications of an open right hemicolectomy to hospital trusts within NHS England? A cost analysis. *BMJ Open*. 2021;11(12)
10. Downey CL, Bainbridge J, Jayne DG, Meads DM. Impact of in-hospital postoperative complications on quality of life up to 12 months after major abdominal surgery. *Br J Surg*. Aug 11 2023;110(9):1206-1212. doi:10.1093/bjs/znad167
11. Leeds IL, Drabo EF, Lehmann LS, Safar B, Johnston FM. On All Accounts: Cost-Effectiveness Analysis of Limited Preoperative Optimization Efforts Before Colon Cancer Surgery. *Dis Colon Rectum*. Jun 1 2021;64(6):744-753. doi:10.1097/DCR.0000000000001926
12. Briggs AH, O'Brien BJ, Blackhouse G. Thinking outside the box: recent advances in the analysis and presentation of uncertainty in cost-effectiveness studies. *Annual review of public health*. 2002;23:377-401. doi:10.1146/annurev.publhealth.23.100901.140534
13. Fung A, Trabulsi N, Morris M, et al. Laparoscopic colorectal cancer resections in the obese: a systematic review. *Surg Endosc*. May 2017;31(5):2072-2088. doi:10.1007/s00464-016-5209-y
14. Yang T, Wei M, He Y, Deng X, Wang Z. Impact of visceral obesity on outcomes of laparoscopic colorectal surgery: a meta-analysis. *ANZ J Surg*. Jul-Aug 2015;85(7-8):507-13. doi:10.1111/ans.13132
15. Cakir H, Heus C, van der Ploeg TJ, Houdijk AP. Visceral obesity determined by CT scan and outcomes after colorectal surgery; a systematic review and meta-analysis. *Int J Colorectal Dis*. Jul 2015;30(7):875-82. doi:10.1007/s00384-015-2174-1
16. Collaborative P-AT. SupPoRtive Exercise Programmes for Accelerating REcovery after major ABdominal Cancer surgery trial (PREPARE-ABC): Pilot phase of a multicentre randomised controlled trial. *Colorectal Dis*. Aug 6 2021;doi:10.1111/codi.15856

© 2025 Koutoukidis DA et al. *JAMA Network Open.*

17. ACS. ACS NSQIP Surgical Risk Calculator. Accessed 07 April 2025, https://riskcalculator.facs.org/RiskCalculator/
18. McKechnie T, Kazi T, Shi V, et al. Preoperative very low-energy diets for obese patients undergoing intra-abdominal colorectal surgery: a retrospective cohort study (RetroPREPARE). *Tech Coloproctol*. Oct 1 2024;28(1):134. doi:10.1007/s10151-024-03015-0
19. Mahmood WU, Lubbad O, Shafique S, Mahmood U, Baig MK, Sajid MS. Effect of multimodal prehabilitation versus no prehabilitation in patients undergoing colorectal resections: a systematic review and meta-analysis. British Journal of Surgery; 2025:
20. Xu W, Wells CI, McGuinness M, et al. Characterising nationwide reasons for unplanned hospital readmission after colorectal cancer surgery. *Colorectal Dis*. May 2023;25(5):861-871. doi:10.1111/codi.16467
21. Wick EC, Shore AD, Hirose K, et al. Readmission rates and cost following colorectal surgery. *Dis Colon Rectum*. Dec 2011;54(12):1475-9. doi:10.1097/DCR.0b013e31822ff8f0
22. Kim HJ, Choi GS, Park JS, et al. Long-term Complications of Laparoscopic or Robotic Lateral Pelvic Node Dissection After Preoperative Chemoradiotherapy for Locally Advanced Rectal Cancer. *Dis Colon Rectum*. Apr 1 2024;67(4):505-513. doi:10.1097/DCR.0000000000003183
23. Braga M, Frasson M, Vignali A, Zuliani W, Civelli V, Di Carlo V. Laparoscopic vs. open colectomy in cancer patients: long-term complications, quality of life, and survival. *Dis Colon Rectum*. Dec 2005;48(12):2217-23. doi:10.1007/s10350-005-0185-7
24. Beck C, Weber K, Brunner M, et al. The influence of postoperative complications on long-term prognosis in patients with colorectal carcinoma. *Int J Colorectal Dis*. Jun 2020;35(6):1055-1066. doi:10.1007/s00384-020-03557-3
25. Liu L, Herrinton LJ, Hornbrook MC, Wendel CS, Grant M, Krouse RS. Early and late complications among long-term colorectal cancer survivors with ostomy or anastomosis. *Dis Colon Rectum*. Feb 2010;53(2):200-12. doi:10.1007/DCR.0b013e3181bdc408
26. Wan Y, Lim S, Riebman J, Jamous N, Gao X. Clinical and Economic Burden Associated with Anastomotic Leak After Colorectal Surgeries in the United Kingdom. *Value in Health*. 2014;17(3):A76-A77. doi:10.1016/j.jval.2014.03.447
27. Mualla NM, Hussain MR, Akrmah M, Malik P, Bashir S, Lin JJ. The Impact of Postoperative Complications on Long-Term Oncological Outcomes Following Curative Resection of Colorectal Cancer (Stage I-III): A Systematic Review and Meta-Analysis. *Cureus*. Jan 21 2021;13(1):e12837. doi:10.7759/cureus.12837
28. Borstlap WAA, Westerduin E, Aukema TS, Bemelman WA, Tanis PJ, Dutch Snapshot Research G. Anastomotic Leakage and Chronic Presacral Sinus Formation After Low Anterior Resection: Results From a Large Cross-sectional Study. *Annals of surgery*. Nov 2017;266(5):870-877. doi:10.1097/SLA.0000000000002429
29. Petrosyan Y, Thavorn K, Maclure M, et al. Long-term Health Outcomes and Health System Costs Associated With Surgical Site Infections: A Retrospective Cohort Study. *Annals of surgery*. May 1 2021;273(5):917-923. doi:10.1097/SLA.0000000000003285
30. Knechtle WS, Perez SD, Medbery RL, et al. The Association Between Hospital Finances and Complications After Complex Abdominal Surgery: Deficiencies in the Current Health Care Reimbursement System and Implications for the Future. *Annals of surgery*. Aug 2015;262(2):273-9. doi:10.1097/SLA.0000000000001042
31. Gan TJ, Robinson SB, Oderda GM, Scranton R, Pepin J, Ramamoorthy S. Impact of postsurgical opioid use and ileus on economic outcomes in gastrointestinal surgeries. *Curr Med Res Opin*. Apr 2015;31(4):677-86. doi:10.1185/03007995.2015.1005833
32. Wick EC, Hirose K, Shore AD, et al. Surgical site infections and cost in obese patients undergoing colorectal surgery. *Archives of surgery*. Sep 2011;146(9):1068-72. doi:10.1001/archsurg.2011.117
33. Lawson EH, Hall BL, Louie R, et al. Association between occurrence of a postoperative complication and readmission: implications for quality improvement and cost savings. *Annals of surgery*. Jul 2013;258(1):10-8. doi:10.1097/SLA.0b013e31828e3ac3

© 2025 Koutoukidis DA et al. *JAMA Network Open.*

34. D'Souza J, Eglinton T, Frizelle F. Readmission prediction after colorectal cancer surgery: A derivation and validation study. *PLoS One*. 2023;18(6):e0287811. doi:10.1371/journal.pone.0287811
35. Ali D, Syed M, Gamboa AC, et al. Risk Factors for Delayed (>30 Days) Readmission Following Rectal Cancer Surgery. *J Surg Res*. Feb 2025;306:397-406. doi:10.1016/j.jss.2024.12.037
36. Caminsky NG, Moon J, Marinescu D, Pang AJ, Vasilevsky CA, Boutros M. Timing of readmissions for complications following emergency colectomy: follow-up beyond post-operative day 30 matters. *Surg Endosc*. Apr 2024;38(4):2240-2251. doi:10.1007/s00464-024-10724-y
37. Daniels I, Tuson R, Hargreaves J. What are the financial implications of an open right hemicolectomy to hospital trusts within NHS England? A cost analysis. *BMJ Open*. 2021;11(12):e053187.

© 2025 Koutoukidis DA et al. *JAMA Network Open.*
