# Supplementary material for: Preoperative Weight Loss in Patients With Excess Weight and Colorectal Cancer: The CARE Feasibility Randomized Clinical Trial
Source: JAMA Netw Open. 2025 Dec 8;8(12):e2547126. doi: 10.1001/jamanetworkopen.2025.47126 (PMC12687096; doi:10.1001/jamanetworkopen.2025.47126)
Supplement: Supplement 3. — Data Sharing Statement [file jamanetwopen-e2547126-s003.pdf]

Data Sharing Statement

Koutoukidis. Preoperative Weight Loss in Patients With Excess Weight and Colorectal Cancer. *JAMA Netw Open*. Published December 08, 2025. doi:10.1001/jamanetworkopen.2025.47126

Data
Additional Information: https://www.isrctn.com/ISRCTN39207707
Data available: Yes
Data types: Deidentified participant data
How to access data: Deidentified individual participant data are available from the corresponding authors upon reasonable request. All proposals requesting data access will need to complete a data request form with details of the research question and the analysis plan.
When available: With publication

Supporting Documents
Document types: Statistical/analytic code
How to access documents: Statistical/analytic code available from the corresponding authors upon reasonable request.
When available: With publication

Additional Information
Who can access the data: researchers whose proposed use of the data has been approved
Types of analyses: any purpose
Mechanisms of data availability: signed data access agreement
